# Probing Weak Halogen Bonding in Aqueous Solution

**DOI:** 10.1021/jacs.6c04848

**Published:** 2026-04-28

**Authors:** Manuel A. Herbst, Leyun Wu, Yannik T. Woordes, Stefan Peintner, Jian Xin, Frank M. Boeckler, Zhijian Xu, Weiliang Zhu, Armando Navarro-Vázquez, Mate Erdelyi

**Affiliations:** † Department of Chemistry for Life Sciences, 8097 Uppsala University, Uppsala SE-751 23, Sweden; ‡ State Key Laboratory of Drug Research; Drug Discovery and Design Center, Shanghai Institute of Materia Medica, Chinese Academy of Sciences, Shanghai 201203, China; ± Department of Pharmacy and Biochemistry, 9188 Eberhard Karls University Tübingen, Tübingen 72076, Germany; Δ Departamento de Química Fundamental, Centro de Ciências Exatas e da Natureza, 28116 Universidade Federal de Pernambuco, Recife, Pernambuco 50740-560, Brazil; ⊥ Center of Excellence for the Chemical Mechanisms of Life, 8097 Uppsala University, SE-751 23 Uppsala, Sweden

## Abstract

Noncovalent interactions are as important for chemistry and biology as they are difficult to characterize. They determine the 3D structure of biopolymers, the binding mode and affinity of drug candidates, and the mechanisms and kinetics of enzymatic reactions and molecular recognition events, to name a few examples of processes that take place in aqueous solution. A single noncovalent force, with its inherent weakness, yields transient low-affinity complexes that are especially challenging to detect in dilute solutions, especially in polar and protic solvents that offer competing interactions, such as water. Using weak halogen bonding (<20 kJ mol^–1^) as an example, we showcase a strategy to characterize very weak interactions in dilute aqueous solution using NMR spectroscopy. We characterize the relative strength of a set of weak iodine and bromine-centered halogen bonds by describing their ability of stabilizing a β-hairpin motif, whose population was quantified using NOE- and *J*-coupling-based ensemble analysis, and describe the geometry of the halogen bond based on bond orientations with ^1^H-^13^C residual dipolar couplings. Density functional theory calculations corroborate the experimentally observed trends. Our work presents one of the very first detailed experimental characterizations of halogen bonding in aqueous solution and a methodology that allows systematic assessment of this interaction in the solvent most relevant for drug discovery. Due to the modularity of the presented model system, the strategy is easily transferable to the characterization of other single, weak noncovalent interactions, such as hydrogen, chalcogen, pnictogen, and tetrel bonds, Coulomb forces, or π interactions in dilute aqueous solution.

## Graphical abstract

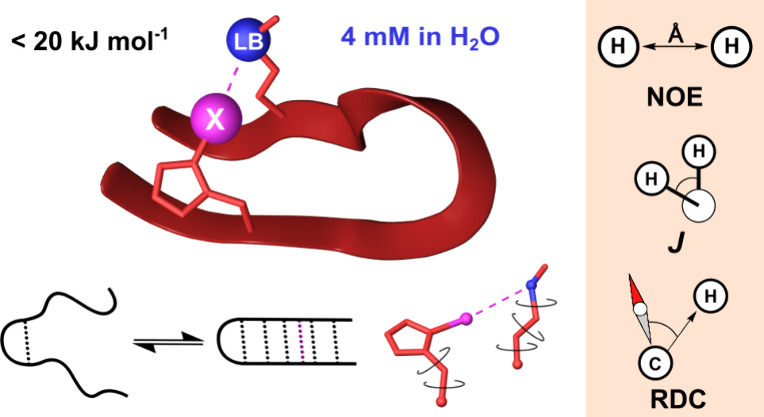

## Introduction

Noncovalent interactions are ubiquitous in nature and are pivotal for molecular structure and function.^1,2^ As they are weak, typically <20 kJ mol^–1^, their far-reaching impact is often attributed to synergistic effects. However, even a single weak noncovalent bond can have a large influence on the conformation and the activity of biomolecules.^3–5^ An improved understanding of such interactions can pave the way for improvements of, for instance, supramolecular chemistry,^6^ organocatalysis,^7^ structural biology^8^ and medicinal chemistry.^9^

Due to their weak nature, single noncovalent bonds are not just difficult to investigate experimentally, but even their detection poses a challenge in solutions at the low concentrations that are relevant for chemical and biological processes. Accordingly, we find examples of computational^10–15^ and crystallographic^4,16^ studies of single noncovalent bonds, whereas their experimental studies in solution^17,18^ or in the gaseous phase^19^ are rare. As noncovalent interactions often occur between a Lewis acid/base pair,^20–23^ they are especially cumbersome to characterize in polar and protic media,^18,24–27^ such as water, which easily competes with the interaction. A third challenge is posed by the low probability of the encounter of the interaction partners in dilute solutions. Studies of single, weak noncovalent forces in dilute aqueous solutions would nevertheless be the most relevant for improving our understanding of their role for the structure and function of biological systems as well as for the development of green chemical processes.^28,29^ Obtaining such experimental data is, however, by far not trivial.

In order to bridge this gap, herein, we present a strategy for the characterization of a single weak noncovalent interaction in a dilute aqueous solution. We demonstrate its scope by characterizing a single, weak halogen bond^20^ embedded into a protein-like environment. Most solution studies of halogen bonds so far have used (i) strong—often positively charged^30–34^ or perfluorinated^24,35^ —halogen bond donors, (ii) the Lewis base in huge excess to maximize the population of the halogen bonded complex,^36,37^ and (iii) noncompetitive aprotic solvents.^38^ Being a major challenge, studies of halogen bonding in dilute aqueous solutions remain scarce.^39–41^ To demonstrate the capabilities of the presented strategy, we address the halogen bond of a weak nonfluorinated halogen bond donor at a 1:1 donor:acceptor ratio in a dilute 4 mM aqueous solution.

## Results and Discussion

### Design

Detecting weak halogen bonds, for instance, that between a halo-imidazole and an ether or thioether via solution NMR, remains challenging at millimolar concentrations (Figure 1).^42^ This difficulty arises from low complex populations and minimal binding-induced changes in NMR observables, such as a chemical shift (δ). To overcome these limitations, we utilized a cooperatively folding β-hairpin system (Figure 2a), which provides the entropic advantage of investigating the noncovalent force intramolecularly.^17,43^ Halo-imidazoles and (thio)­ethers were chosen as interaction pairs for this investigation due to their close structural resemblance to the side chain functionalities of natural amino acids and to common elements of drug candidates^44^ and sustainable materials.^45^ Cooperative folding amplifies the structural consequence of the formation of the noncovalent bond of interest, which further facilitates its detection. The population change of the β-hairpin conformer, the folding of which is facilitated by the weak halogen bond, may be quantified by a large number of NMR parameters such as NOEs and scalar couplings. This offers an advantage over earlier approaches that often followed the chemical shift change of a single atom only, nearby the interaction site.^24,46–48^ Molecular balances,^49^ which exploit restricted bond rotation of simplified model systems to quantify the populations of “bound” and “free” conformers isolated by a high, Δ*G* > 65 kJ mol^–1^, rotation barrier, have been elegantly used to quantify weak noncovalent interactions by the integration of distinct NMR signals.^49^ This approach, which has been used to study halogen−π interactions,^50,51^ allows the determination of equilibrium constants within a ∼14.6 kJ mol^–1^ interaction window, by integration of distinct signals of slowly exchanging conformers, with an estimated accuracy of ±0.5 kJ mol^–1^. Our strategy extends the scope to systems in rapid exchange on the NMR time scale, to make population-averaged NMR parameters accessible through ensemble deconvolution.^52^ The characterization of the noncovalent bond of interest is expected to be enabled by the measurement of ^1^H,^13^C residual dipolar couplings (RDCs) that reflect the relative orientations of C–H bonds. In contrast to the local nature of binding-induced chemical shift changes, the magnitude of residual dipolar couplings is indirectly connected to the formation of the noncovalent bond itself, through the overall change of orientation of molecular fragments and functionalities.^53^

**Figure 1:**
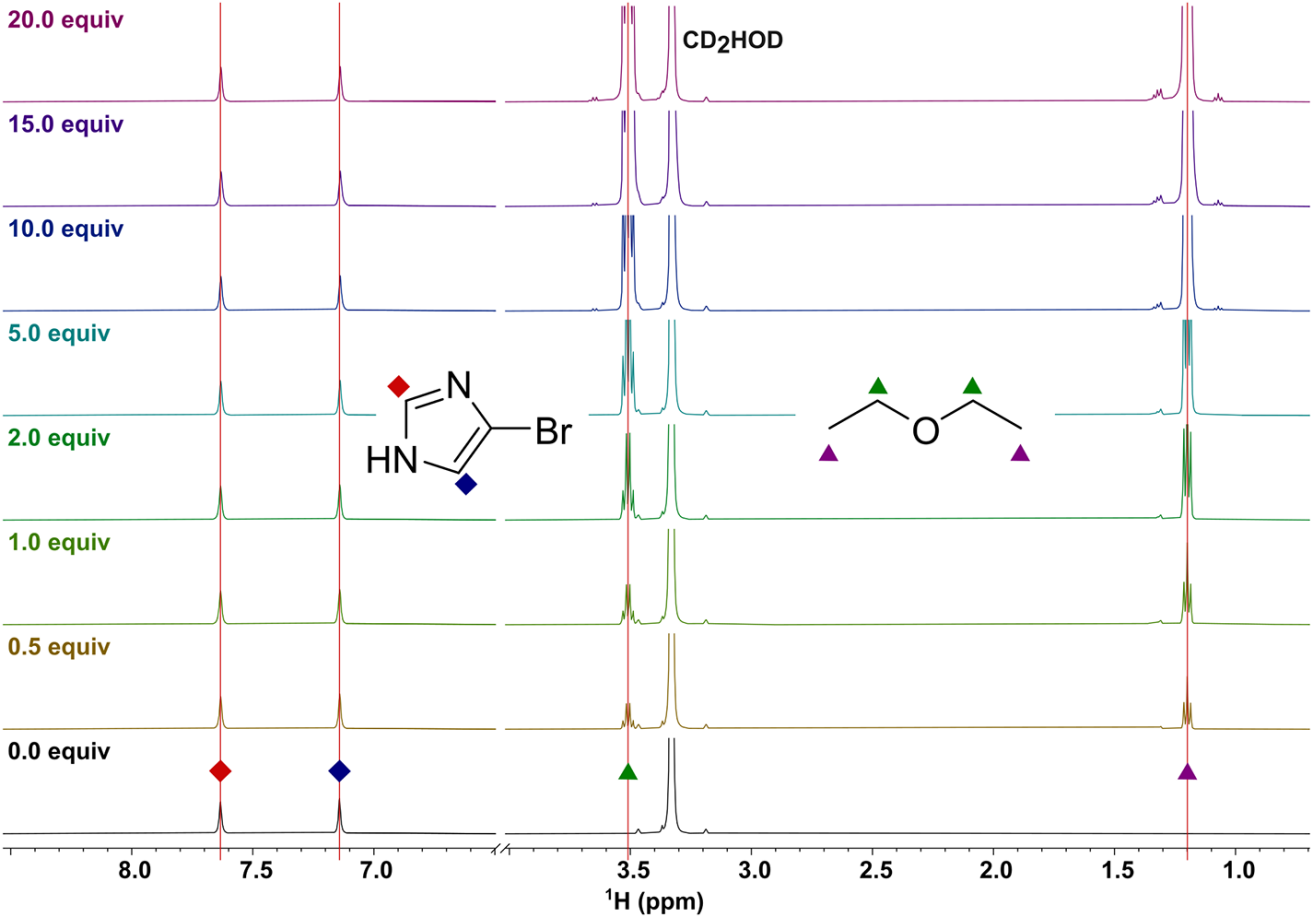
No significant chemical shift changes are observed during the ^1^H NMR titration of 4-bromo-1*H*-imidazole with diethyl ether (500 MHz, 4 mM in CD_3_OD). Upon the addition of 20 equiv diethyl ether, <0.003 ppm chemical shift change could be detected on the signals of 4-bromo-1*H*-imidazole.

**Figure 2:**
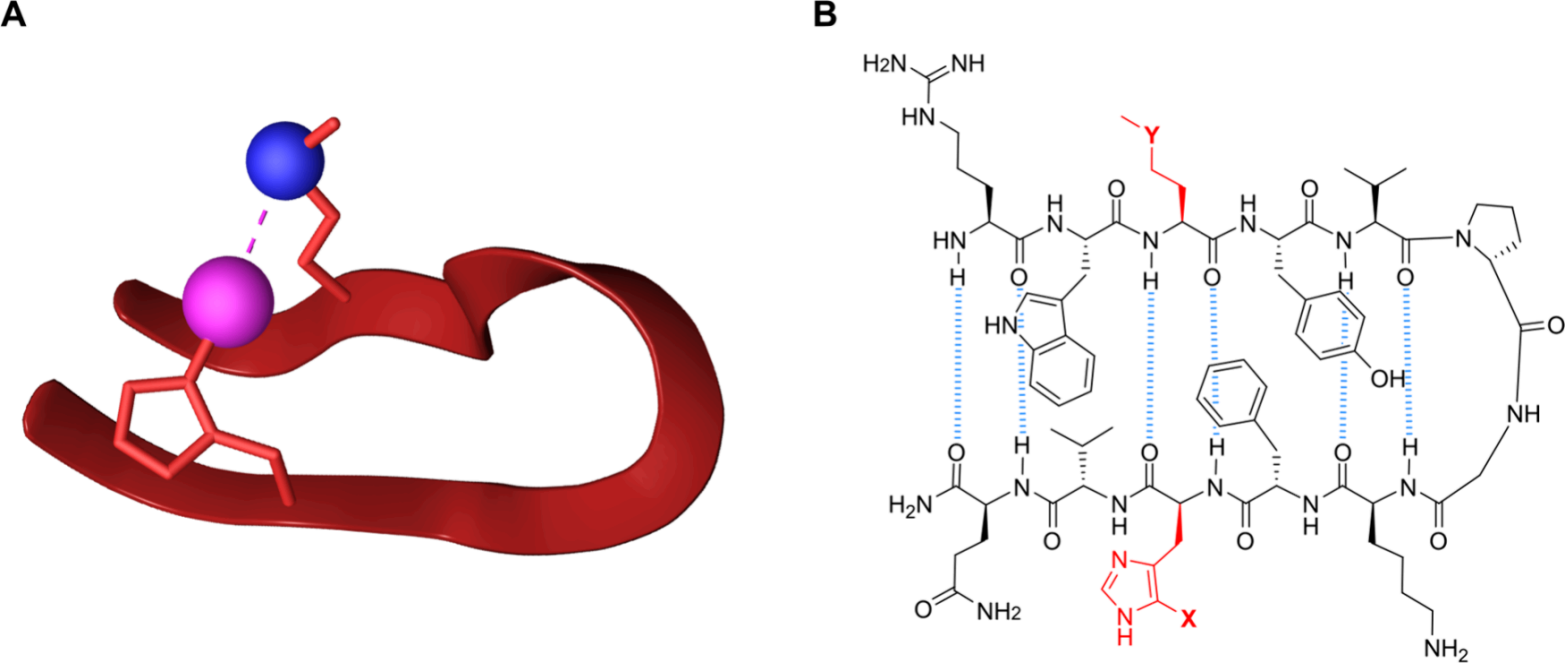
(a) A cooperatively folding β-hairpin model system designed to investigate a single weak interaction, here a halogen bond, in aqueous solution. The halogen bond donor and acceptor are shown as pink and blue spheres, respectively. (b) Structure of compounds **1–5**. Cross-strand hydrogen bonds are depicted as blue dotted lines, side chains that are designed to form a halogen bond are colored in red. For compound **1**, X = H and Y = CH_2_, for **2**, X = Br and Y = O, for **3**, X = Br and Y = S, for **4**, X = I and Y = O, and for **5**, X = I and Y = S. The syntheses of **1**–**5** are given in Section 1 of the Supporting Information.

Our model system was designed to be flexible, enabling the halogen bond donor and acceptor sites (Figure 2b, red) to freely adjust their orientation relative to one another. The system's linear backbone encompasses the type II’ β-turn inducer motif, d-proline-glycine,^54–56^ aligning the two flexible strands to promote cross-strand hydrogen bonds, polar contacts, and hydrophobic interactions. These forces drive β-hairpin folding without enforcing it. To endorse aqueous solubility, arginine, lysine, and glutamine residues possessing hydrophilic side chains were incorporated. The four side chains adjacent to the cross-strand halogen bond interaction site face to the opposite sides of the β-hairpin plane, which helps to avoid unwanted interactions, e.g., hydrogen bonds to the halogen bond acceptor. The flexibility of the linear sequence allows for folding/defolding and side chain rotations without large energy barriers. The model system sequence and the side chain structures constituting the halogen bond donor and acceptor sites were identified through systematic Monte Carlo conformational searches of alternative sequences, altering the position of the halogen bond donor/acceptor sites within the β-hairpin and varying the number of methylene groups to alter the length and orientation of the side chains. The selected overall structure was predicted to permit the formation of an interstrand halogen bond and to maintain approximately 50% overall folding at room temperature. This folding ratio corresponding to the inflection point of a sigmoid curve is expected to allow the detection of the largest changes of population-weighted spectroscopic observables for a two-state equilibrium.^17,57^

To detect the formation of the weak halogen bond in compounds **2**-**5** (Figure 2b), their NMR data is compared to those of reference **1**, the structure of which resembles **2**–**5** in all aspect but its inability to form an attractive cross-strand halogen bond due to C–X to C–H and an O/S to CH_2_ substitution (Figure 2b). Performing this single atom substitution is expected to influence β-hairpin folding to the extent that the single weak halogen bond of **2**–**5** impacts the stability of the folded conformation of the backbone. The strength of the halogen bond can then be characterized by measuring its influence on the folded populations of **2**–**5** compared to the folding of **1**. Here, compound **2** has a Br···O, **3** a Br···S, **4** an I···O, and **5** an I···S halogen bond (Figure 2b). Systematic variation of the halogen and Lewis base in **2–5** ought to reveal whether and to what extent these minor changes in interaction strength are detectable.

### Solution Ensemble Analyses

NMR spectra for **1**–**5** were acquired at 800 MHz on 4 mM aqueous solutions (H_2_O:D_2_O 9:1) with solvent suppression using excitation sculpting.^58^ Interproton distances were obtained by acquiring NOE buildups with 7 mixing times, between 100 and 700 ms, and calculating NOE buildup rates by internal referencing to the buildup rate of the NOE between methylene protons (1.78 Å). ^3^ *J*_NH‑Hα_ coupling constants were read from the amide proton signals in the ^1^H NMR spectra. Conformational searches to generate input ensembles for ensemble fitting with Mnova Stereofitter (MestreLab Research)^59^ were performed with Monte Carlo Multiple Minimum torsional sampling, as implemented in the software package Schrödinger, using the force fields OPLS4, AMBER*, and MMFF94 along implicit solvation models for water and chloroform, each combining the six independent conformational pools and removing duplicates by redundant conformation elimination. This extensive conformational search ensures that the input ensemble fed to Stereofitter covers the entire conformational space^52^ of the backbone of **1**–**5** (Figure 2b). Conformers were classified as folded β-hairpins, when the dihedral angles within the β-turn region complied with those of a type II’ β-turn,^60^ the opposing strands were within an average C_α_-to-C_α_ distance of 6 Å to each other, and at least four of the six interstrand hydrogen bonds shown in Figure 2b were formed. The present conformational searches are unable to provide all possible combinations of side chain orientations for all amino acids of a dodecapeptide within a reasonable time. Therefore, only NOEs and *J*-couplings of backbone CH_α_ and NH atoms were used to estimate the β-hairpin population, without attempting to determine the orientations of each and every side chain with high accuracy. Further details of the NMR data acquisition and processing are given in Sections 2 and 3 of the Supporting Information.

The molar fractions of the folded and the unfolded states of compounds **1**–**5** in aqueous solution were determined by analysis of the NMR ensemble based on the population-averaged experimental NOEs and *J*-couplings. These observables^61^ were deconvoluted using the software Mnova StereoFitter (Mestrelab Research).^59^ This approach^52^ has previously been successfully applied to determine the solution ensemble of flexible molecules of similar size, such as macrocycles,^43,62,63^ peptides,^17^ and a variety of drug candidates.^64,65^ In short, the NOE-derived interproton distances and the ^3^ *J*_NH‑Hα_ coupling constant-derived backbone dihedral angles^66^ describe the population averaged orientation of the backbones of **1**–**5**. The non-negative linear least-squares deconvolution algorithm of StereoFitter identifies the solution conformers and their populations by varying the populations of all theoretically possible conformers and comparing these calculated population-averaged NMR observables to the corresponding experimentally observed ensemble-averaged data. The deconvolution is independent from computed energies, which are not necessarily reliable,^67^ and the fitting is solely driven by experimental data.

### Noncovalent Bonding Characterized by Quantifying Folding

Our strategy was to address a noncovalent force that is too weak to be directly detectable, by incorporating it into the antiparallel strands of a cooperatively folding β-hairpin and quantifying its impact on hairpin folding. An attractive interstrand interaction is expected to stabilize the folded conformation of **2**–**5** to an extent that is correlated with the interaction strength. In line with our expectations, the introduction of an interstrand halogen bond increases β-hairpin folding (Figure 3) as compared to the folding of **1**, the reference compound that lacks the ability of attractive interstrand interaction at the corresponding positions. Our NOE and *J*-based ensemble determination revealed that **1** has 22% folded β-hairpin conformation, whereas compounds **2** and **3** that are capable of forming a Br···O or a Br···S halogen bond were 44 and 45% folded, respectively. Compounds **4** and **5** that contain iodine as a halogen bond donor as well as oxygen and sulfur as a halogen bond acceptor were found to possess 40 and 65% β-hairpin populations, respectively. The extent of folding of **1**–**5** is in agreement with iodine-centered halogen bonds being stronger than bromine-centered ones^68,69^ and sulfur being a stronger halogen bond acceptor than oxygen.^70^ Compound **5** that possesses the strongest halogen bond, I···S, is thus the most folded of the studied compounds. It shows a 3-fold increase in folding as compared to reference **1** and a nearly 50% increase in folding in relation to analogue **3** that is stabilized by a weaker Br···S halogen bond. For **4** and **5** stabilized by I···O and I···S halogen bonds, respectively, the extent of folding reflects that sulfur is a stronger halogen bond acceptor (65% folded) compared to oxygen (40% folded). The corresponding trend is observable but less extensive for the systems possessing the weaker halogen bond donor bromine, with **2** and **3** being 44 and 45% folded, respectively. The lower population of folded conformers for compound **4** (I···O, 40%) compared to **2** (Br···O, 44%) is unexpected. The population difference might be due to the different size of iodine and bromine, requiring different geometries for forming a halogen bond, and is comparable to the accuracy of the method for determining populations (up to 5%).^52^ Notably, the population-based estimation of interaction energies is most sensitive to population changes within the 40–60% range.

**Figure 3:**
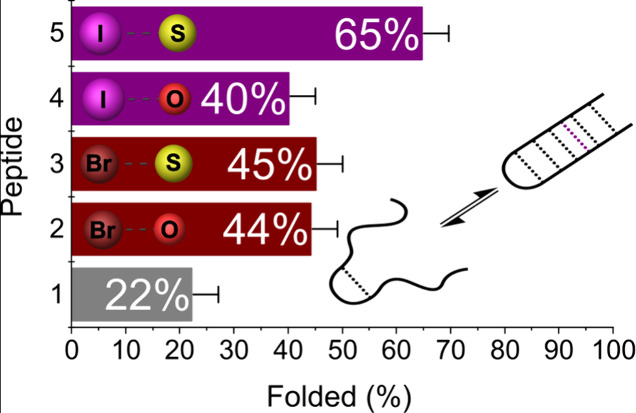
Folded β-hairpin populations for compounds **1**–**5**, as determined by NOE- and *J-*coupling-based ensemble analysis using the software StereoFitter. The ensemble fitting has been estimated to provide up to 5% accuracy.^52^ The increased folding of the β-hairpin conformers is consistent with the increasing strength of the interchain halogen bond.

The energy preferences for the β-hairpin motif derived from the above obtained populations compared to reference **1** (Figure 3) correspond to 1–2 kJ mol^–1^ for the weak Br···O, Br···S, and I···O halogen bonds (compounds **2**-**4**), and up to 3 kJ mol^–1^ for the I···S halogen bond (**5**), when estimated by the Boltzmann population distribution. These energies are in line with the expected strength of weak (nonfluorinated, nonionic) halogen bonds in a polar and protic solvent.^17,43,71^ It should be highlighted that ^1^H NMR is usually seen to be limited to the detection of interactions ≥5 kJ mol^–1^ in energy.^72^ Competition experiments have been shown to push this limit to Δ*G*° ≥ 1.7 kJ mol^–1^;^73^ however, there are only a few association constants in the range of 1–2 M^–1^ reported experimentally so far and their accuracy has been disputed. Hence, the detection of interactions with strengths of 1–3 kJ mol^–1^ at 4 mM concentration, with the order of strengths corresponding to that expected based on theory, indicates the usefulness of the presented strategy. This is also one of the very first examples of the experimental detection and characterization of halogen bonds in an aqueous solution.^41^

### Halogen Bond Geometry

The detection of the relative orientation of the interacting functional groups in solution remains a major challenge. The geometry of such interactions is currently typically characterized by X-ray diffraction or predicted by computations. We describe herein the relative orientation of the halogen bond donor and acceptor functionalities of **2**–**5** in aqueous solution based on the orientation information gained from residual dipolar couplings (RDCs),^53,74,75^ which were detected in F1-coupled ^1^H,^13^C CLIP-HSQC spectra.^76^ Partial alignment was achieved by compressing a poly­(4-acryloylmorpholine-*co*-acrylonitrile) gel to 70% of its original length.^77^ RDCs were determined by calculating the difference between the coupling constants detected on the sample with (*T*_strong_) and those without (*T*_weak_) compression. They ranged between −13 and +6 Hz, which were smaller than those measured for the rigid brucine *N*-oxide in the same alignment medium (−14 to +30 Hz, see Section 1.3.3 of the Supporting Information).^78^ This downscaling of the RDCs is in agreement with conformational averaging due to the flexibility of compounds **2**–**5** compared to brucine *N*-oxide.

Ensemble fitting was performed by simultaneously optimizing the alignment tensor components, via the singular value decomposition (SVD), and conformer populations as implemented into the software StereoFitter,^79^ making use of the ^1^H,^13^C one-bond RDCs of the halogen bond donor and acceptor side chains as well as of those corresponding to the α position of each amino acid of the model system (Figure 4). For a reliable determination of the alignment tensor, >5 independent RDCs are required,^81,82^ which requirement is fulfilled when using overall 15 RDCs detected for the C–H bonds of the side chains involved in the interstrand halogen bond and for the C_α_–H_α_ bonds of **2**–**5** (Section 5, Supporting Information). The input conformational pool, covering all theoretically available orientations of the halogen bond donor and acceptor functionalities of interest, was generated by resampling the orientation of the side chains that possess the halogen bond donor and acceptor functionalities for each conformer of the solution ensemble identified using NOEs and *J*-couplings (vide supra). This preselection of valid backbone conformations drastically reduced the size of the conformational space, streamlining the sampling of side chain orientations. Monte Carlo multiple minimum sampling was performed with 10,000 Monte Carlo and 5000 molecular mechanics minimization steps, and redundant conformers were eliminated based on the heavy atom positions (1.5 Å RMSD cutoff). As current force fields implemented in Schrödinger are not parametrized for halogen bonding, the ensemble was enriched with additional conformers with constrained halogen bond geometries (for details, see Section 3 of the Supporting Information). Simultaneous singular value decomposition (SVD) analyses of the experimental RDCs and optimization of conformer populations of each theoretically possible conformer were used to determine the alignment tensor as well as to identify the conformers present in solution along with their population (molar fraction) by minimizing the difference between experimental and back-calculated RDCs. The experimental versus back-calculated RDCs are shown in Figure 4, and further details of the analyses are given in Section 5 in the Supporting Information. The low Cornilescu quality factors^83^ of the correlation fits suggest that the singular value decomposition analyses sufficiently well identified the halogen-bonded conformers and their populations. This suggests that the halogen bonded conformers added to the input conformational pool of the fitting encompassed good enough models of the conformers present in the solution, and we expect that the future availability of force fields updated with parameters for halogen bonding will corroborate this conclusion.

**Figure 4:**
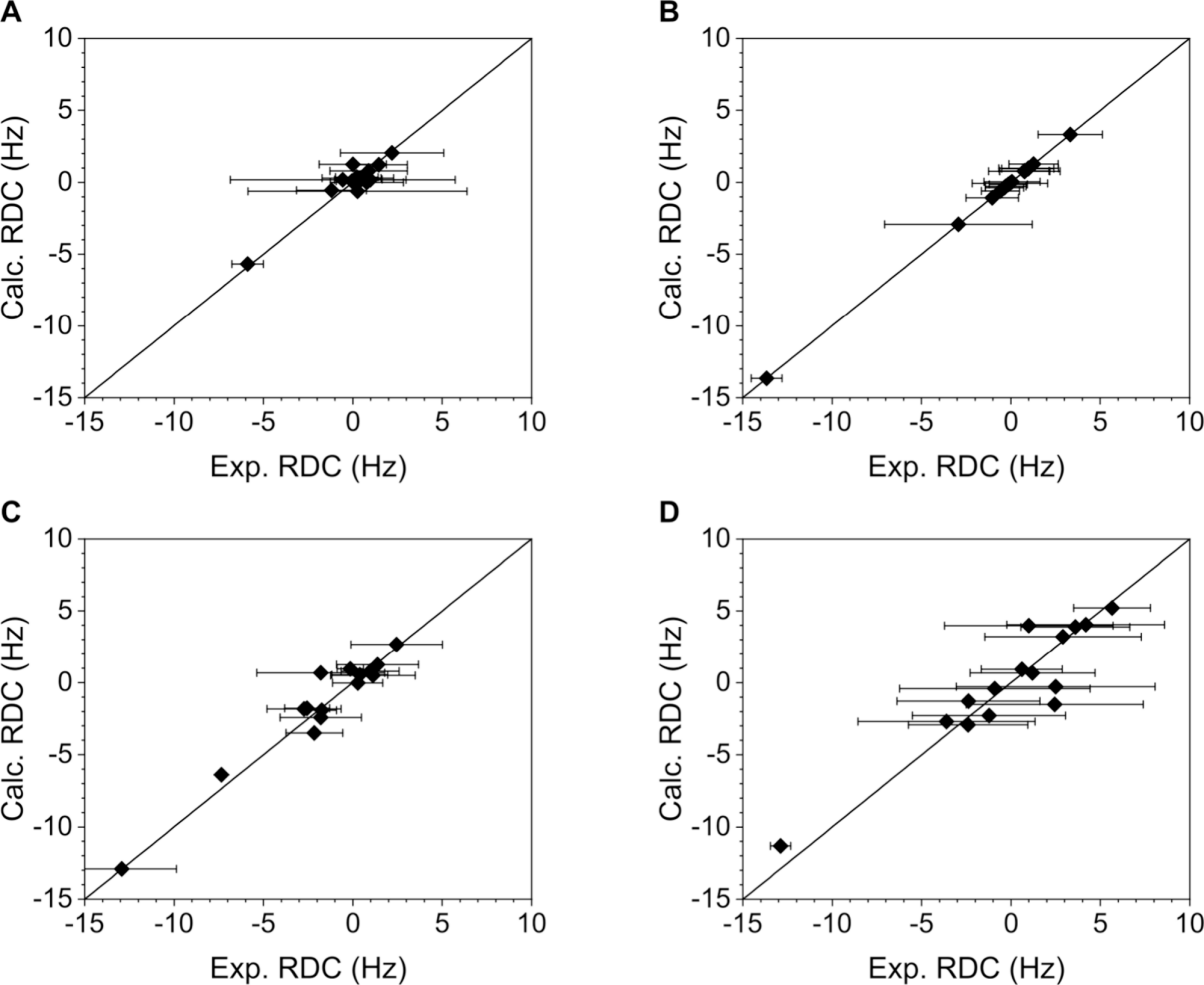
Correlation of experimental and back-calculated RDCs, determined by singular value decomposition using the software MSpin for (a) compound **2** (*Q* = 0.322, CN = 16.7), (b) compound **3** (Q = 0.005, CN = 12.7), (c) compound **4** (*Q* = 0.219, CN = 9.2), and (d) compound **5** (*Q* = 0.376, CN = 5.5). Here, *Q* denotes the Cornilescu quality factor^83^ that is a commonly accepted measure of the goodness-of-fit of the theoretical model (structure) to the experimental data, with *Q* < 0.3 indicating a good and *Q* < 0.4 an acceptable fit. The condition number, CN, reflects the reliability of the determination of the alignment tensor, with an SVD < 30 reflecting a robust fit.^82^ Experimental errors were calculated from the signal half-line width of the NMR signals and their signal per noise ratio (see Section 2.3 of the Supporting Information) and are portrayed as horizontal error bars.

The populated conformers were classified as halogen-bonded when the halogen bond donor–acceptor distance is equal or shorter than the sum of van der Waals radii of the participating atoms (I/Br and O/S) and the C–I/Br···O/S angle is >120°.^8^ The populations of the halogen-bonded conformers are listed in Figure 5. The gradual population increase of halogen-bonded conformers from compound **2** to **5** is in line with the expectations as iodine is a better halogen bond donor than bromine, and sulfur a better acceptor than oxygen.^35^ This finding confirms the direct correlation between the expected halogen bond strength and the observed probability of halogen bond formation. It further agrees with the hypothesis that the population of the folded state, as determined by the prior backbone analysis, reflects the strength of the stabilizing interstrand noncovalent interaction. Here, we wish to emphasize that the NOE- and *J*-based backbone ensemble analysis (Figure 3) reports on the overall backbone folding only, without giving direct information on the halogen bonding of the corresponding side chains. The RDC analysis builds on the above and provides direct information on the halogen bond of the side chains, including their geometry and population. These two pieces of information are interconnected but are not identical. Fitting NOE, *J*, and RDC data simultaneously is possible with the software Stereofitter. However, fitting all data simultaneously would be counterproductive for highly flexible molecules, such as **1**–**5**, because it is difficult, if not impossible, to describe the flexibility of both the backbone and the amino acid side chains simultaneously with the currently available computational algorithms. It is more straightforward to determine the backbone conformations first, and solve the geometry of the side chains in a subsequent step.

**Figure 5:**
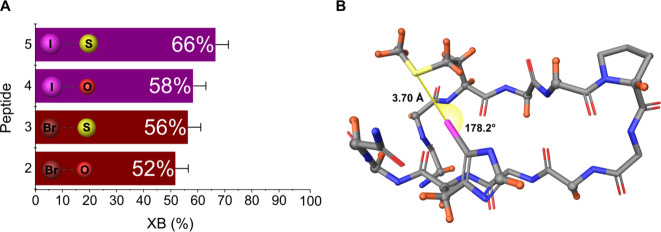
(a) The populations of halogen-bonded conformers (XB (%)) for compounds **2**–**5** as determined by singular value decomposition as implemented in the software StereoFitter, fitting C–H residual dipolar couplings. (b) The highest populated conformer (30%) with a halogen bond formed between the halogen bond donor iodine and acceptor sulfur for **5**. Residual dipolar couplings were obtained for C–H bonds, highlighted in orange.

The dihedral angles and interatomic distances in the halogen-bonded β-hairpin conformers of **2**–**5**, identified by deconvolution using StereoFitter, are in good agreement with that expected based on the previous literature.^35^ Hence, the geometry of halogen bonds can be assessed by RDC-based ensemble analysis, in a protic and polar solution, for interactions so weak that they are difficult to detect even just with standard NMR techniques (Figure 1). Nevertheless, the presented strategy also has inherent limitations. For instance, due to the limited temperature range permitted by the melting and boiling points of water, a detailed thermodynamic analysis of the folding/defolding process via detection of a chemical shift ‘melting curve’^17^ was not feasible. Using a H_2_O:D_2_O (9:1) mixture as solvent allows detection of NOEs of amide protons, which facilitates the characterization of the solution ensembles; however, the need for solvent suppression in NOESY experiments increases the experimental error of distance determination. Detection of these NOEs without solvent suppression would not be possible due to the limitations of the receiver’s dynamic range.

### Quantum Chemical Computations

Density functional theory (DFT) calculations were performed to address the bond geometries and energies of the halogen bonds of compounds **2**–**5**. The comprehensive investigation of the low energy conformations of the entire molecules with DFT would have been infeasible, and therefore, the halogen bonds were analyzed using simplified models (see Figure 6 and Section 6 of the Supporting Information for further details).

**Figure 6:**
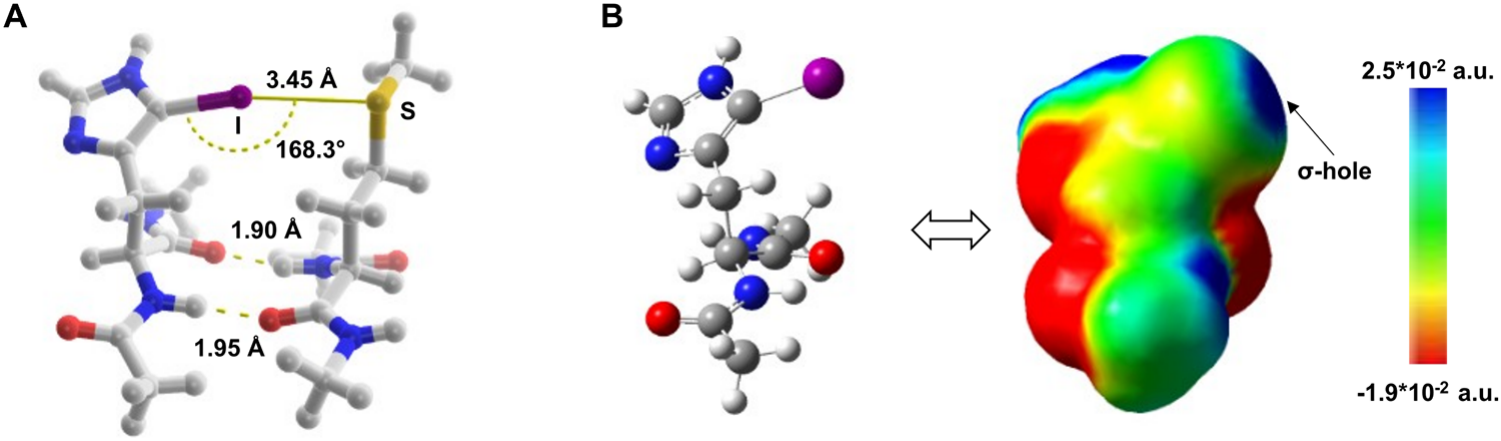
(a) The equilibrium geometry for the iodohistidine-methionine dimer 5* corresponding to the halogen bonded fragment of **5**. (b) The computed electrostatic potential surface map of iodohistidine, defined by 0.0004 au electron density isosurfaces.

Hence, five dimeric pairs, 1*–5*, each connected by two ‘backbone’ hydrogen bonds and four of them offering the possibility of halogen bonding corresponding to those of **2**–**5** (I/Br – S/O) as well as a reference corresponding to **1** were optimized with DFT (for details, see the Supporting Information). The computed halogen bond lengths and bond angles for the model systems 2*–5* indicate feasible bond geometries with bond distances shorter than the sum of van der Waals radii of the participating atoms (*d*_Br–O_ = 3.02 Å (90% of the ∑vdW) and C–Br···O angle 165.8° for 2*; *d*_Br–S_ 3.42 Å (94% of the ∑vdW) and C–Br···S angle 169.2° for 3*; *d*_I–O_ = 3.05 Å (87% of the ∑vdW) and C–I···O angle 166.4° for 4*; d_I–S_ 3.45 Å (91% of the ∑vdW) and C–I···S angle 168.3° for 5*, see Figure 6a and Figure S23 of the Supporting Information), where ∑vdW is the sum of the van der Waals radii of the atoms participating in the interaction.^84^ The corresponding computed molecular electrostatic potential surfaces indicate σ-hole (electropositive region) formation on the halogens (Figure 6b and Figure S24 of the Supporting Information), suggesting that formation of a halogen bond is viable for peptides **2**–**5.**

To estimate the energy associated with the halogen bonding of **2**–**5**, we first calculated the interaction energy of the reference dimer 1* (His­(H)···Nle­(CH_2_), where Nle denotes norleucine) lacking halogen bond donor and acceptor sites, with the backbone geometry being kept frozen. Its interaction energy of −43.9 kJ mol^–1^ is dominated by the two hydrogen bonds of the dimer. The contribution of halogen bonds was estimated by subtracting the energy of 1* from the energies calculated for the halogen bonded dimers 2*–5* (Table 1). These DFT-computed absolute energies are certainly crude estimates that suffer from large uncertainities;^85^ however, the calculated energies are in agreement with the literature,^9^ and the observed trends correlate with the population of halogen-bonded conformers as estimated using RDCs (Figure 5). They thus corroborate that the origin of the increased stabilization of the β-hairpin conformation of **2**–**5** as compared to **1** is interstrand halogen bonding.

**Table 1:** Calculated Interaction Energies of Optimized Structures for Model Dimers 1*–5*

	1* (H···H)	2* (Br···O)	3* (Br···S)	4* (I···O)	5* (I···S)
Δ*E* (kJ mol^–1^)	–43.9	–54.6	–55.2	–59.8	–61.5
ΔΔ*E* (kJ mol^–1^)	0.00	–10.7	–11.3	–15.9	–17.6

## Conclusions

Herein, we presented a strategy to study single, weak noncovalent forces in dilute aqueous solution and demonstrated its scope by describing halogen bonds that are otherwise undetectable by standard NMR techniques, such as chemical shift titrations. To achieve this, we incorporated the halogen bond into a modular and cooperatively folding model system. The modular nature allows the easy adjustment of the compound’s polarity, facilitating studies in different solvent environments.^17,43^ To gain an entropic advantage, we studied the halogen bonds intramolecularly. Cooperativity with other noncovalent interactions makes the weak halogen bond detectable by observation of its influence on folding, that is, on the population of the folded conformers. We quantified the folded population using nuclear Overhauser effect- and scalar coupling-driven ensemble analysis and identified the population of the halogen-bonded conformers using residual dipolar couplings. Using the presented strategy, a 1 kJ mol^–1^ change in interaction strength corresponds to ∼15% change in folding. The obtained halogen bond geometries and trends of interaction energies were in agreement with that expected based on the literature and predicted by density functional theory calculations (Figure 7). The correlation of the computationally estimated relative interaction energies ΔΔ*E* with the experimental halogen-bonded populations is clear yet is not linear. This is explained by a combination of the experimental error (Figure 4), the known inaccuracy of energies estimated by density functional theory (>5 kJ mol^–1^),^85,86^ the vastly simplified model (two isolated amino acids) of **1**–**5** used for the computational work, the difficulty in modeling the different solvation properties of iodine vs bromine,^87^ and the different van der Waals radii of iodine and bromine as well as oxygen and sulfur that may influence the efficiency of stabilization of the folded β-hairpin conformers via the corresponding halogen bonds. Using the presented strategy, we disclose one of the first systematic investigations of weak halogen bonds in aqueous solution.

**Figure 7:**
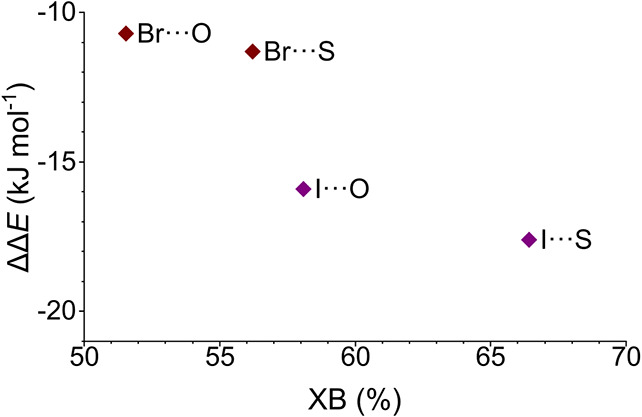
Relative interaction energies ΔΔ*E* (kJ mol^–1^) of the halogen bonds in compounds **2**–**5**, as estimated by DFT, correlate with the experimentally determined populations of their halogen-bonded conformers, XB (%).

As the NMR observables used for the description of folding and the orientation of the interacting functional groups are interaction force-independent, the presented strategy ought to be easily utilized for the systematic investigation of any type of noncovalent forces, such as hydrogen, chalcogen, pnictogen, and tetrel bonds, and in any solvent of interest, including the most difficult one, water.

Molecular recognition processes are driven by noncovalent interactions. Their rational applications in drug development, supramolecular chemistry, and catalysis depend on the understanding of the energies and geometries of the weak forces involved. A strategy capable of providing experimental data on noncovalent forces at concentrations and in solvents that have previously not been possible to assess is expected to initiate a myriad of applications. The data provided herein on halogen bonding in water may be helpful for drug development as well as for the parametrization of halogen bonding in common force fields.

## Supplementary Material



## Notes

NMR and computational data, detailed information on the synthesis; original NMR FIDs, NMReDATA files, Stereofitter and MSpin input and output files including structure files (sdf), and DFT-optimized geometries are freely available online at Zenodo with doi:10.5281/zenodo.16900969 (PDF)
