# Supplementary material for: Probing Weak Halogen Bonding in Aqueous Solution
Source: J Am Chem Soc. 2026 Apr 28;148(18):19331–40. doi: 10.1021/jacs.6c04848 (PMC13184976; doi:10.1021/jacs.6c04848)
Supplement: Supplementary file 1 [file ja6c04848_si_001.pdf]

Supporting information

Probing Weak Halogen Bonding in Aqueous Solution

Manuel A. Herbst,[†] *Leyun Wu,*[†,‡] *Yannik T. Woordes,*[†] *Stefan Peintner,*[†] *Jian Xin,*[±] *Frank M. Boeckler,*[±] *Zhijian Xu,*[‡] *Weiliang Zhu,*[‡] *Armando Navarro-Vázquez,*[Δ] *Mate Erdelyi*[†,‖,*]

[†] Department of Chemistry for Life Sciences, Uppsala University, Uppsala SE-751 23, Sweden

[‡] State Key Laboratory of Drug Research; Drug Discovery and Design Center, Shanghai Institute of Materia Medica, Chinese Academy of Sciences, Shanghai, 201203, China

[±] Department of Pharmacy and Biochemistry, Eberhard Karls University Tübingen, Tübingen 72076, Germany

[Δ] Departamento de Química Fundamental, Centro de Ciências Exatas e da Natureza, Universidade Federal de Pernambuco, Recife, Pernambuco, 50740-560, Brazil

[‖] Center of Excellence for the Chemical Mechanisms of Life, Uppsala University,Uppsala SE-751 23, Sweden

Table of Contents

1 Experimental section **3**

1.1 Synthesis of Fmoc-protected halogenated histidines 3

1.1.1 Synthetic procedures 4

1.2 Peptide synthesis 7

1.3 Alignment medium 8

1.3.1 Synthesis of alignment media 8

1.3.2 Swelling and compression 9

1.3.3 Residual dipolar couplings (RDCs) of brucine *N*-oxide 11

2 NMR spectroscopy **12**

2.1 Chemical shift assignments 12

2.2 Interproton distances derived from NOE build-ups 16

2.3 Residual dipolar couplings (RDCs) 21

2.4 Amide temperature coefficients 24

2.5 Titration of diethyl ether to 4-bromo-1*H*-imidazole 26

3 Computational conformational sampling **26**

3.1 Conformational sampling for backbone analysis 26

3.2 Conformational sampling for side chain analysis 29

4 Backbone analysis using NOEs and *J*-couplings **30**

5 Side chain analysis using RDCs and NOEs **49**

6 DFT calculations **56**

7 NMR spectra **58**

8 HRMS spectra **84**

9 References **87**

Original NMR FIDs, NMReDATA files, Stereofitter input and output files including structure files (sdf) are freely available online at Zenodo with doi:10.5281/zenodo.16900969.

1 Experimental section

1.1 Synthesis of Fmoc-protected halogenated histidines

Chemicals and reagents were obtained from commercial sources and used as received. Reactions were monitored by thin-layer chromatography (TLC) carried out on Merck Kieselgel 60 F254 plates (Merck, Darmstadt, Germany) and visualized under UV light (254 nm and 366 nm) or by analytical high-performance liquid chromatography (HPLC). HPLC was performed on an UltiMate 3000 HPLC system (Thermo Fisher Scientific Inc. Waltham, MA, USA) equipped with a ReproSil-XR 120 C18 column (4.6 × 150 mm, 5 µm, 120 Å, Dr. Maisch GmbH, Tübingen, Germany).

Chromatographic purifications were carried out on an Interchim PuriFlash 4250 or PuriFlash XS520Plus system (Interchim, Montluçon, France) using normal-phase column chromatography (Silica gel 60, particle size: 0.025–0.04 mm and 0.04–0.063 mm, Machery-Nagel, Düren, Germany) or equipped with a C18 column (puriFlash® C18-HP 15µm) using reversed-phase column chromatography. Absorption was detected at 218 nm, 254 nm, 280 nm, and 360 nm.

Mass spectrometry (MS) was performed on AmaZon SL (Bruker Corporation, Billerica, MA, USA) using positive or negative electrospray ionization (ESI) or MSD 5977 (Agilent Technologies Inc., Santa Clara, CA, USA) using electron ionization (EI) and high-resolution mass spectrometry was recorded on a maXis 4G (Bruker Corporation, Billerica, MA, USA) using positive electrospray ionization (HR-ESI-TOF) coupled with an UltiMate 3000 HPLC system (Thermo Fisher Scientific Inc. Waltham, MA, USA).

NMR spectra were acquired using Bruker Avance III HDX 400 MHz and Bruker Avance III HDX 600 MHz spectrometers (Bruker Corporation, Billerica, MA, USA). ^{1}H, ^{13}C, and ^{19}F NMR chemical shifts (δ) were reported in parts per million (ppm) relative to tetramethylsilane (TMS) and calibrated using the residual peak of the solvent used. Coupling constants (J) were reported in hertz (Hz). The following abbreviations were used to describe signal multiplicities: s (singlet), d (doublet), q (quartet), m (multiplet), and bs (broad singlet).The purity of all final synthesized compounds was >95%, as determined by HPLC analysis

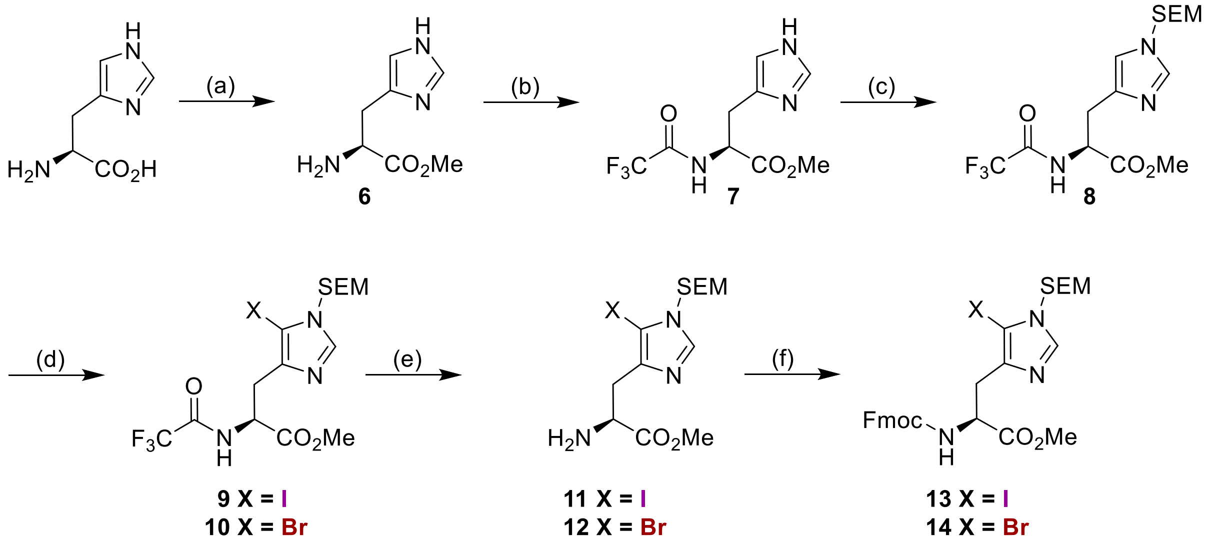

Scheme S1. Synthesis of Fmoc-protected halogenated histidine. Reagents and conditions: (a) thionyl chloride, MeOH; (b) TEA, ethyl trifluoroacetate; (c) 1equiv NaH, 1.06 equiv SEM-Cl, 0 °C - rt; (d) iodine, $NaHCO_3$ or NBS; (e) NaOH aqueous solution; (f) Na_2CO_3, Fmoc-Cl.

The side-chain halogenated histidine derivatives **13** and **14** (Scheme S1), were synthesized directly from L-histidine.

Initially, L-histidine was dissolved in dry methanol, and thionyl chloride was added dropwise via dropping funnel in ice-bath conditions. The mixture was then stirred at room temperature over night to afford L-histidine methyl ester (**6**).[1-4] After removal of the solvent, triethylamine was added at low temperature, followed by slow addition of ethyl trifluoroacetate. Stirring at room temperature for 3.5 hours yielded **7.**

After protecting both the amino and carboxyl groups, a SEM (2-(trimethylsilyl) - ethoxymethyl) protecting group was introduced onto the imidazole ring to obtain **8**, thereby improving solubility in organic solvents and facilitating subsequent peptide synthesis.

Subsequently, halogenation was performed under mild conditions to selectively introduce halogen atoms at the C5 position, controlling the ratio of mono- versus dihalogenated products and obtaining mono-halogenated derivatives **9** and **10**. Treatment with 2 equiv of 1 M NaOH aqueous solution under ice-bath conditions, followed by stirring at room temperature for 18 h, enabled deprotection of the methyl ester to yield amino acids **11** and **12**. Finally, Fmoc protection was carried out under sodium carbonate aqueous conditions to afford Fmoc-protected amino acids **13** and **14**.

1.1.1 Synthetic procedures

Methyl L-histidinate (6). To a suspension of L-histidine (6 g, 38.7 mmol) in 200 ml anhydrous MeOH, 1.2 equiv thionyl chloride (3.37 ml, 46.4 mmol) was added dropwise at 0 °C. The reaction was then stirred at room temperature over night. After the reaction was complete, the solvent was evaporated and **6** (6 g, 93 %) obtained as a white solid. ^{1}H NMR (400 MHz, DMSO - d_6) δ (ppm): 9.06 (d, *J* = 1.4 Hz, 1H), 7.52 (d, *J* = 1.3 Hz, 1H), 4.48 (t, *J* = 7.0 Hz, 1H), 3.72 (s, 3H), 3.33 (d, *J* = 7.0 Hz, 2H); ^{13}C NMR (100 MHz, DMSO - d_6) δ (ppm): 168.58, 134.04, 126.78, 118.06, 53.06, 51,06, 25.11. ESI-MS (m/z): $[M+H]^+$ = 170.05.

Methyl (2,2,2-trifluoroacetyl)-L-histidinate (7). 6 g of methyl L-histidinate (**6**) were dissolved in 200 mL methanol. 3 equiv triethylamine (15 ml, 106 mmol) at 0 °C were added and stirred at room temperature for 15 minutes. Subsequently, 1.1 equiv ethyl trifluoroacetate (4.6 ml, 38 mmol) was added dropwise at 0 °C, then stirred at room temperature for 3.5 hours. After completion of the reaction, the solvent was removed to get the crude product, which was purified by silica gel column chromatography (eluent: DCM / MeOH 90:10) to provide **7** (8.5 g, 91 %) as a brown solid. ^{1}H NMR (400 MHz, DMSO - d_6) δ (ppm): 9.93 (d, J = 7.7 Hz, 1H), 7.64 (d, J = 1.2 Hz, 1H), 6.87 (d, J = 1.2 Hz, 1H), 4.65 (td, J = 8.1, 5.3 Hz, 1H), 3.67 (s, 3H), 3.13 – 2.99 (m, 2H); ^{13}C NMR (100 MHz, DMSO - d_6) δ (ppm): 170.86, 156,56 (q, *J*cf = 37.23), 135.60, 133.81, 116.40, 114.74(q, *J*cf = 285.84), 53.27, 52.82, 28.38. ^{19}F NMR (376 MHz, DMSO - *d6*) δ (ppm): 74.44. ESI - MS (m/z): $[M+H]^+$ = 266.03.

Methyl *N*$^{\alpha}$-(2,2,2-trifluoroacetyl)-*N*$^{\tau}$-((2-(trimethylsilyl)ethoxy)methyl)-L-histidinate (8). 8.5 g of methyl (2,2,2-trifluoroacetyl)histidinate (**7**) was dissolved in 100ml anhydrous THF, then 1.1 equiv NaH (0.85 g, 35 mmol) was added portionwise under 0 °C. The mixture was stirred then for 1 h at room temperature. 1 equiv SEM-Cl (6.2 ml, 35 mmol) was added at 0 °C drop wise and the reaction stirred over night at room temperature. The reaction was then quenched with 20 ml water and the solvent removed by an evaporator. The mixture was diluted with H_2O (50 mL) and extracted with EtOAc (3×50ml). Combined organic layers were then concentrated. The solvent was removed in vacuo to obtain the crude product, which was then purified by silica gel column chromatography (eluent: EtOAc / n-Hexane 50:50) to provide **8** (6 g, 50%) as colorless oil. ^{1}H NMR (400 MHz, DMSO - d_6) δ (ppm): 8.92 – 8.84 (m, 1H), 7.57 (d, *J* = 1.3 Hz, 1H), 6.84 (t, *J* = 1.0 Hz, 1H), 5.21 (s, 2H), 4.82 (dt, *J* = 7.7, 4.8 Hz, 1H),

3.68 (s, 3H), 3.48 – 3.39 (m, 2H), 3.22 (dd, *J* = 15.0, 4.9 Hz, 1H), 3.08 (ddd, *J* = 15.0, 4.7, 1.0 Hz, 1H), 0.92 – 0.83 (m, 2H), 0.03 (s,9H). ^{13}C NMR (100 MHz, DMSO - d_6) δ (ppm): 170.19, 157,27(q, *J*cf = 37.23), 137.32, 137.26, 116.80, 115.97(q, *J*cf = 285.84), 76.23, 66.68, 52.75, 52.64, 28.55, 17.79, -1.37. ^{19}F NMR (376 MHz, DMSO - d_6) δ (ppm): -76.02. ESI-MS (m/z): $[M+H]^+$ = 396.24.

Methyl (*S*)-3-(5-iodo-1-((2-(trimethylsilyl)ethoxy)methyl)-1*H*-imidazol-4-yl)-2-(2,2,2-trifluoroacet-amido) propanoate (9). Compound **8** (6g, 15.2mmol) was dissolved in 20ml *N,N*-dimethylformamide (DMF), 3 equiv $NaHCO_3$ (3.8 g, 45.6 mmol) and 3 equiv iodine (11.6 g, 45.6 mmol) added, and the mixture was then stirred overnight at 35 °C. When the reaction was complete, 2 ml sodium thiosulfate were added to quench the reaction and the mixture was diluted with 50 ml EtOAc and washed with 50 ml water 3 times. The combined organic layers were then concentrated. Crude compound was purified by silica gel column chromatography (EtOAc / n-Hexane 0:100 - 30:70) to provide **9** (5 g, 64 %) as yellow oil.^{1}H NMR (400 MHz, $CDCl_3$) δ (ppm): 8.61 (d, *J* = 7.8 Hz, 1H), 7.87 (s, 1H), 5.25 (s, 2H), 4.87 (dt, *J* = 7.9, 4.8 Hz, 1H), 3.73 (s, 3H), 3.55 – 3.46 (m, 2H), 3.27 (dd, *J* = 15.1, 5.1 Hz, 1H), 3.09 (dd, *J* = 15.2, 4.7 Hz, 1H), 0.96 – 0.85 (m, 2H), 0.01 (s,9H). ^{13}C NMR (100 MHz, $CDCl_3$) δ (ppm): 169.92, 157,29 (q, *J*cf=37.23) 141.71, 140.39, 120.20, 114.48 (q, *J*cf = 285.84), 76.99, 71.37, 66.87, 52.96, 52.00, 28.92, 17.81, -1.31. ^{19}F NMR (376 MHz, $CDCl_3$) δ (ppm): -75.89. ESI-MS(m/z): $[M+H]^+$ = 522.21.

Methyl (*S*)-3-(5-bromo-1-((2-(trimethylsilyl)ethoxy)methyl)-1*H*-imidazol-4-yl)-2-(2,2,2-trifluoro-acetamido) propanoate (10). **8** (6 g, 15.2 mmol) was dissolved in 50 ml acetonitrile. Then 1.1 equiv *N*-bromosuccinimide (2.97 g, 16.7 mmol) were added portionwise under 0 °C, then the mixture was stirred for 40 min at room temperature. When the reaction was complete, 2 ml sodium thiosulfate was added to quench the reaction. Acetonitrile was remove by an evaporator. The mixture was diluted with 50 mL water and extracted 3 times with 50ml EtOAc. The organic layers were combined and concentrated. Crude compound was purified by silica gel column chromatography (EtOAc / n-Hexane 0:100 - 30:70) to provide **10** (3 g, 43 %) as yellow oil. ^{1}H NMR (400 MHz, $CDCl_3$) δ (ppm): 8.49 (d, *J* = 7.8 Hz, 1H), 7.78 (s, 1H), 5.27 (d, *J* = 0.9 Hz, 2H), 4.87 (dt, *J* = 7.8, 4.8 Hz, 1H), 3.73 (d, *J* = 0.6 Hz, 3H), 3.58 – 3.47 (m, 2H), 3.27 (ddd, *J* = 15.1, 5.2, 1.1 Hz, 1H), 3.07 (ddd, *J* = 15.1, 4.5, 1.3 Hz, 1H), 0.95 – 0.85 (m, 2H), -0.02(s,9H). ^{13}C NMR (100 MHz, $CDCl_3$) δ (ppm): 169.93, 157.27(q, *J*cf = 37.62), 138.14, 135.43, 115.89(q, *J*cf = 285.84), 102.70, 75.34, 66.98, 52.95, 51.91, 27.76, 17.80, -1.34. ^{19}F NMR (376 MHz, $CDCl_3$) δ (ppm): -75.93. ESI-MS (m/z): $[M+H]^+$ = 474.2.

(*S*)-2-amino-3-(5-iodo-1-((2-(trimethylsilyl)ethoxy)methyl)-1*H*-imidazol-4-yl) propanoic acid (11). To a solution of **9** (5g, 9.6 mmol) in MeOH (30 mL) was added aq. 1 M NaOH (19.2 mL). The reaction mixture was stirred at room temperature for 18 h. MeOH was removed in vacuo and crude compound purified by silica gel column chromatography (DCM / MeOH 80:20) to provide **11** (3.5 g, 89 %) as white solid. ^{1}H NMR (400 MHz, $CDCl_3$) δ (ppm): 8.65 (s, 1H), 5.35 (s, 2H), 4.24 (s, 1H), 3.63 – 3.54 (m, 2H), 3.41 (d, *J* = 13.6 Hz, 1H), 3.27 (d, *J* = 16.0 Hz, 1H), 0.98 – 0.89 (m, 2H), 0.01 (s,9H). ^{13}C NMR (100 MHz, $CDCl_3$) δ (ppm): 173.39, 163.51, 163.15, 139.89, 137.50, 116.31, 80.37, 76.41, 69.36, 54.26, 28.69, 19.22, 0.00. ESI-MS (m/z): $[M+H]^+$ =412.04 .

(*S*)-2-amino-3-(5-bromo-1-((2-(trimethylsilyl)ethoxy)methyl)-1*H*-imidazol-4-yl)propanoic acid (12). To a solution of **10** (3 g, 6.3 mmol) in MeOH (30 mL) was added aq. 1 M NaOH (12.6 mL). The reaction mixture was stirred at room temperature for 18 h. MeOH was removed in vacuo, crude compound purified by silica gel column chromatography (DCM / MeOH 80:20) to provide **12** (2 g, 87 %) as white solid. ^{1}H NMR (400 MHz, $CDCl_3$) δ (ppm): 8.57 (s, 1H), 5.39 (s, 2H), 4.25 (t, *J* = 7.0 Hz, 1H), 3.65 – 3.56 (m, 2H), 3.41 (dd, *J* = 15.5, 4.7 Hz, 1H), 3.28 (dd, *J* = 15.8, 8.2 Hz, 1H), 0.99 – 0.89 (m, 2H), 0.01(s,9H). ^{13}C NMR (100 MHz, $CDCl_3$) δ (ppm):173.16, 163.94, 163.58, 163.22, 162.85, 138.20, 132.02, 119.18, 116.29, 107.34, 69.60, 53.96, 27.31, 19.26, 0.00. ESI-MS(m/z): $[M+H]^+$ =364.20 .

(*S*)-2-(((9*H*-fluoren-9-yl)methoxy)carbonyl)amino)-3-(5-iodo-1-((2-(trimethylsilyl)ethoxy)methyl)-1*H*-imidazol-4-yl)propanoic acid (13). To a solution of **11** (3.5 g, 8.5 mmol) in 1,4-dioxane (15 mL) was added at 0 °C a 20% Na_2CO_3 solution (6.7 mL, 25.5 mmol, 3 equiv), followed by 1.1 equiv Fmoc-Cl (2.4 g, 9.4 mmol). The reaction mixture was stirred for 2 h at room temperature. H_2O was added and the mixture was washed with Et_2O, then EtOAc was added to the aq. layer and the pH was adjusted at 0 °C with conc. HCl to pH = 1–2. The biphasic mixture was extracted three times with EtOAc, then solvent was removed in vacuo. The crude product was purified by reversed-phase chromatography (C18, eluent: H_2O and ACN with 0.1% TFA) to provide **13** (3 g, 55 %) as a white solid. ^{1}H NMR (400 MHz, $CDCl_3$) δ (ppm): 9.40 (s, 1H), 8.60 (s, 1H), 7.75 (d, *J* = 7.4 Hz, 2H), 7.63 – 7.55 (m, 2H), 7.43 – 7.25 (m, 5H), 6.03 (d, *J* = 7.0 Hz, 1H), 5.33 (d, *J* = 2.6 Hz, 2H), 4.67 – 4.59 (m, 1H), 4.46 (dd, *J* = 10.6, 6.9 Hz, 1H), 4.37 (dd, *J* = 10.6, 6.5 Hz, 1H), 4.17 (t, *J* = 6.6 Hz, 1H), 3.60 – 3.51 (m, 2H), 3.29 (s, 1H), 0.95 – 0.86 (m, 2H), -0.02 (s, 9H). ^{13}C NMR (100 MHz, $CDCl_3$) δ (ppm): 173.84, 157.24, 145.30, 145.21, 142.95, 142.68, 139.47, 138.33, 129.27, 129.21, 128.62, 128.54, 126.63, 121.48, 80.64, 76.16, 69.60, 68.21, 54.89, 48.61, 30.97, 19.13, 0.00. HR-ESI-MS (m/z) Calcd for $C_{27}H_{32}IN_3O_5Si$ $[M+H]^+$, 633.12342, found: 634.12320.

(*S*)-2-(((9*H*-fluoren-9-yl)methoxy)carbonyl)amino)-3-(5-bromo-1-((2-trimethylsilyl)ethoxy)methyl)-1*H*-imidazol-4-yl)propanoic acid (14). To a stirred solution of **12** (2 g, 5.5 mmol) in 1,4-dioxane (15 mL) at 0 °C was added a 20% Na_2CO_3 solution (4.3 mL, 16.5 mmol, 3 equiv), followed by Fmoc-Cl (1.57 g, 6.1 mmol, 1.1 equiv). The reaction mixture was warmed to room temperature and stirred for 2 h. The solvent was removed under reduced pressure, and the residue was washed with H2O (20 mL) and Et_2O (20 mL). The aqueous phase was retained, cooled to 0 °C, and acidified with concentrated HCl to pH 1–2. The resulting mixture was extracted with EtOAc (3 × 30 mL). The combined organic extracts were concentrated under reduced pressure. The crude product was purified by reversed-phase chromatography (C18; eluent: H_2O/ACN, 0.1% TFA) to provide compound **14** (2.0 g, 62 %) as a white solid. ^{1}H NMR (400 MHz, $CDCl_3$) δ (ppm): 9.78 (s, 1H), 8.63 (s, 1H), 7.75 (d, *J* = 7.6 Hz, 2H), 7.58 (t, *J* = 8.1 Hz, 2H), 7.39 (t, *J* = 7.5 Hz, 2H), 7.30 (td, *J* = 7.5, 1.3 Hz, 2H), 6.13 (d, *J* = 7.1 Hz, 1H), 5.40 (d, *J* = 1.5 Hz, 2H), 4.66 (q, *J* = 6.1 Hz, 1H), 4.35 (dd, *J* = 7.1, 2.9 Hz, 2H), 4.17 (t, *J* = 7.1 Hz, 1H), 3.63 – 3.54 (m, 2H), 3.38 (dd, *J* = 15.0, 5.5 Hz, 1H), 3.27 (dd, *J* = 14.9, 5.6 Hz, 1H), 0.95 – 0.86 (m, 2H), -0.02(s,9H). ^{13}C NMR (100 MHz, $CDCl_3$) δ (ppm): 173.73, 163.73, 157.35, 145.28, 145.16, 142.83, 142.74, 137.68, 132.86, 129.26, 128.59, 126.70, 126.59, 121.49, 107.49, 69.80, 68.58, 54.64, 48.54, 29.76, 19.15, 0.00. HR-ESI-MS (m/z) Calcd for $C_{27}H_{32}BrN_3O_5Si$ $[M+H]^+$, 585.13729, found: 586.13753.

1.2 Peptide synthesis

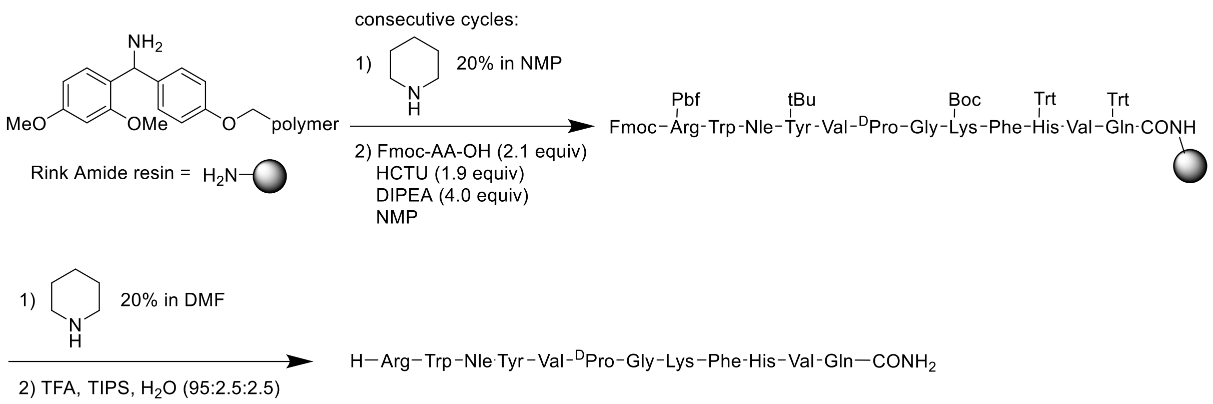

Scheme S2. Synthetic scheme for peptide **1**. Amino acids are depicted with three-letter code. Fmoc-AA-OH = Fmoc-Gln(Trt)-OH, Fmoc-Val-OH, Fmoc-His(Trt)-OH, Fmoc-Phe-OH, Fmoc-Lys(Boc)-OH, Fmoc-Gly-OH, Fmoc-D-Pro-OH, Fmoc-Val-OH, Fmoc-Trp-OH, Fmoc-Nle-OH, Fmoc-Tyr-OH, Fmoc-Arg(Pbf)-OH.

Peptides were synthesized on a 67 µmol scale by semi-automated solid-phase peptide synthesis (SPPS) on a Prelude Peptide Synthesizer (Protein Technologies) using the N_α-Fmoc protection scheme. Amino acids with the following side chain protecting groups were used: *tert*-Butyl (tBu) for Tyr, 2,2,4,6,7-pentamethyldihydrobenzofuran-5-sulfonyl (Pbf) for Arg , *tert*-butyloxycarbonyl (Boc) for Lys and trityl (Trt) for His and Gln. NovaPEG Rink amide resin (loading 0.44 mmol/g) was used as the solid-phase. Before initiating the synthesis, the resin was allowed to swell in DMF overnight. The pre-swelled Rink amide resin was Fmoc-deprotected by treatment with piperidine (20% in *N*-methyl-2-pyrrolidone (NMP), 8 mL, 2 x 5 min). After washing with NMP (7 x 8 mL), amino acid solution in DMF (125 mM, 2.1 equiv), *O*-(1H-6-chlorobenzotriazole-1-yl)-1,1,3,3-tetramethyluronium hexafluorophosphate (HCTU) (0.45 M in DMF, 1.9 equiv), diisopropylethylamine (DIPEA) (2 M in NMP, 4.0 equiv) and additional NMP (1.3 mL) was added. The reaction was allowed to proceed for 2 h, washed with NMP (4.5 mL) and repeated once again (double coupling). Non-coupled *N*-termini were capped by treatment with acetic anhydride (5 mL, 5 min) in order to prevent further propagation of truncated peptide chains and washed again with NMP (7 x 8 mL). The cycle was restarted with Fmoc deprotection to allow for coupling of the next amino acid. Final Fmoc deprotection was performed manually using piperidine (20% in DMF, 8 mL, 15 min).

The non-canonical amino acids Fmoc-L-HomoSer(Me)-OH, Fmoc-His(5-Br)(SEM)-OH and Fmoc-His(5-I)(SEM)-OH were coupled manually according to the following protocol. The Fmoc deprotected peptide chain on Rink amide resin was retrieved from the peptide synthesizer and washed with DMF (3 x 5 mL). A solution of the non-canonical amino acid (1.0 equiv), *O*-(7-azabenzotriazol-1-yl)-*N*,*N*,*N*',*N*'-tetramethyluronium hexafluorophosphate (HATU) (0.9 equiv) and DIPEA (2.0 equiv) in DMF (2 mL) was shaken for 20 min. The amino acid solution was added to the resin and the reaction mixture was agitated for 2 h. Solvents were drained and the coupling was repeated once with fresh amino acid solution (double coupling). Solvents were drained and the resin was washed with DMF (3 x 5 mL). In order to evaluate whether the coupling was completed, an aliquot was withdrawn and subjected to Fmoc deprotection by treatment with piperidine (20% in DMF, 500 µL) and shaken for 20 min. Subsequent side chain deprotection and cleavage from the resin was achieved by treatment with a mixture of TFA, triisopropylsilane and H_2O MQ (95:2.5:2.5, 500 µL). The cleavage cocktail was shaken for 45 min. Reagents were removed under airflow and the residue was redissolved in H_2O MQ. LC-MS analysis of the cleaved peptide confirmed successful coupling to the peptide chain. Subsequent

canonical amino acids were coupled in automated fashion according to the protocol described previously.

After completion of the desired sequence, side chain deprotection and cleavage from the resin was accomplished by treatment with a mixture of TFA, triisopropylsilane and H_2O MQ (95:2.5:2.5, 8 mL). The cleavage mixture was shaken on a saw-rocker for 4 h and then filtered. Remaining resin was washed with TFA (20 mL) and filtered. The combined filtrates were concentrated to < 5 mL, washed with ice-cold diethyl ether (40 mL) and centrifuged (10 min, 4400 rpm). Diethyl ether was decanted and the precipitate was vortexed. This procedure was repeated twice. The solvent was evaporated and the precipitate was redissolved in H_2O MQ. To fully dissolve the peptide, NaOH (aq) was added dropwise to pH = 7-8. Purification was performed by preparative HPLC on a VWR LaPrep system using a Kinetex 5 µm C18 F5 100 Å column (250 x 21.2 mm) and a single wavelength detector at λ = 215 nm. A gradient of MeCN + 0.1% formic acid : H_2O + 0.1% formic acid (5-50% MeCN over 30 min) was used as mobile phase with a flow rate of 15 mL/min. Lyophilization afforded peptides **1-5** as white powders. Obtained amounts and yields are summarized in Table S1, whereas HRMS data is given in Table S2.

Table S1. Obtained yields after HPLC purification for peptides **1-5**. The structure of the peptides are shown in Figure S3.

Peptide	Amount (mg)	Amount (µmol)	Yield (%)
**1**	23.5	15.4	23
**2**	30.3	18.7	28
**3**	28.9	18.0	27
**4**	18.5	11.1	17
**5**	26.9	16.3	24

Table S2. HRMS data for peptides **1-5**. The structure of the peptides are shown in Figure S3.

Peptide	Ionization mode	Found ion	Chemical formula	m/z calc.	m/z found
**1**	ESI+	$[M+H]^+$	$C_{75}H_{110}N_{21}O_{14}^+$	1528.8536	1528.8542
**2**	ESI-	$[M-H]^-$	$C_{74}H_{105}BrN_{21}O_{15}^-$	1608.7267	1608.7303
**3**	ESI-	$[M-H]^-$	$C_{74}H_{105}BrN_{21}O_{14}S^-$	1624.7039	1624.7111
**4**	ESI-	$[M-H]^-$	$C_{74}H_{105}IN_{21}O_{15}^-$	1654.7149	1654.7156
**5**	ESI+	$[M+H]^+$	$C_{74}H_{107}IN_{21}O_{14}S^+$	1672.7067	1672.7026

1.3 Alignment medium

1.3.1 Synthesis of alignment media

Polymer gels used as alignment medium for NMR were synthesized following a modified procedure adapted from Farley *et al.*.[5] Monomers were filtered through Al_2O_3 and cotton to remove stabilisers. The inner glass walls of the NMR tubes, the gels were synthesized in, were siliconized using Sigmacote® solution to prevent adhesion. The freshly coated tubes were dried over night at 100 °C, washed with MeOH and dried again at 100 °C for 6 h.

Six variations of a 4-acryloylmorpholine-based copolymer using different co-monomers and monomer ratios were tested (Table S3).

Table S3. Monomer ratios of the gels tested for suitability as alignment medium.

Gel	Monomer x	Monomer y	Monomer z	Ratio x : y : z
**G1**	4-acryloylmorpholine	methyl methacrylate	1,4-butanediol diacrylate	100 : 23 : 1.2
**G2**	4-acryloylmorpholine	acrylonitrile	ethylene glycol diacrylate	100 : 23 : 1.5
**G3**	4-acryloylmorpholine	acrylonitrile	ethylene glycol diacrylate	100 : 23 : 1.2
**G4**	4-acryloylmorpholine	N,N-dimethylacrylamide	1,4-butanediol diacrylate	100 : 23 : 1.2
**G5**	4-acryloylmorpholine	1-vinyl-2-pyrrolidinone	divinyl adipate	100 : 23 : 1.2
**G6**	4-acryloylmorpholine	vinyl acetate	divinyl adipate	100 : 23 : 1.2

A solution containing 4-acryloylmorpholine (2.24 g, 2.00 mL, 1 equiv), acrylonitrile (194 mg, 241 µL, 0.23 equiv), ethylene glycol diacrylate (32.5 mg, 29.7 µL, 0.012 equiv), AIBN (2.00 mg, 0.075 mol%) and DMSO (500 µL) was prepared. The mixture was agitated and degassed by sonication under nitrogen flow for 15 min. The mixture was transferred to 3 mm NMR tubes. The NMR tubes were flame-sealed, immersed in dry sand and put into the oven at 60 °C for 24 h. After cooling to room temperature, poly-4-acryloylmorpholine (p-4-AM) gels were withdrawn from the NMR tubes by breaking the glass. Pieces of 3 cm length were cut. The gel pieces were submerged in DMSO for 24 h. The solvent was decanted and replaced with fresh DMSO leaving the gel pieces submerged for 24 h. This procedure was performed three times. The gel pieces were dried under airflow for 48 h and subsequently dried under reduced pressure at 50 mbar for 24 h. Transparent gel pieces were obtained after drying further at 40 °C for 3 h.

1.3.2 Swelling and compression

The gels were subjected to a swelling test in various solvents used commonly used for NMR spectroscopy (see Table S4) and compression in water (Table S5).

The gels were synthesized in 3 mm NMR tubes (inner diameter ca. 2.4 mm). In order to prevent bending while compressing in 5 mm tubes (inner diameter ca. 4.2 mm), the swollen gel needs to almost reach the tube wall, *i.e.* a diameter swelling factor close to 1.75. In water, only gel **G3** came close to this degree of swelling (see Table S4). Hence, gel **G3** is the only suitable for the use as alignment medium in water. In addition it is the only tested gel that did withstand compression (see Table S5). Gels **G1**, **G4** and **G6** also showed promising swelling properties in $CHCl_3$.

A

B

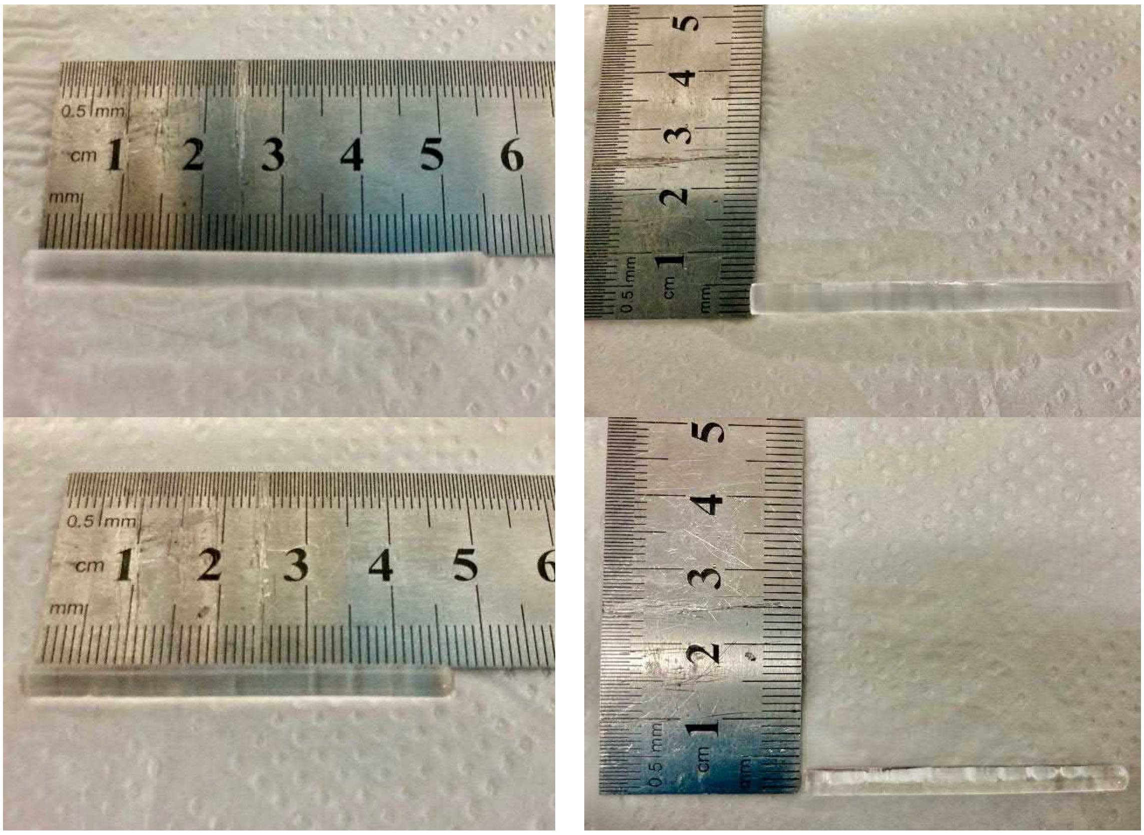

Figure S1. (A) Gel **G1** after swelling in $CHCl_3$ for 24 h. Freshly prepared gel (30 x 3 mm) expanded to 55 x 5 mm. (B) Gel **G3** after swelling in DMSO for 24 h. Freshly prepared gel (30 x 3 mm) expanded to 46 x 3 mm.

Table S4. Swelling factors (length x diameter) in respective solvents.

Gel	H_2O	Acetone	CH_3CN	CH_3OH	$CHCl_3$	CH_2Cl_2	DMF	DMSO	Pyridine	Toluene
**G1**	1.1x1.3				1.8x1.7					
**G2**	1.2x1.3				1.5x1.0					
**G3**	1.3x1.6							1.5x1.0		
**G4**	1.9x1.3	1.1x1.0	1.6x1.3	1.2x1.0	1.8x1.7	1.9x1.3	1.7x.1.3	1.6x1.3	1.9x1.5	1.0x0.7
**G5**	1.5x1.2	1.2x1.0	1.6x1.0	1.3x1.0	1.7x1.3	1.7x1.5	1.4x1.0	1.5x1.2	1.5x1.2	1.0x0.7
**G6**	1.9x1.3	1.2x1.0	1.6x1.0	0.9x0.8	1.8x1.7	1.9x1.3	1.9x1.2	1.8x1.3	2.0x1.3	1.0x0.7

Table S5. Lengths of relaxed and compressed gels in water (mm).

Gel	Relaxed	Compressed	Factor	Behaviour
**G1**	39	15	0.38	bends
**G2**	33	19	0.58	brakes
**G3**	40	26	0.65	good
**G4**	38	28	0.74	brakes
**G5**	36	32	0.89	brakes
**G6**	40	35	0.88	bends

1.3.3 Residual dipolar couplings (RDCs) of brucine *N*-oxide

The alignment capabilities of gel **G3** were tested by extracting residual dipolar couplings for brucine *N*-oxide. Weakly and strongly aligned f2-coupled $^{1}H,^{13}C$-HSQC spectra were recorded (see Section 2).

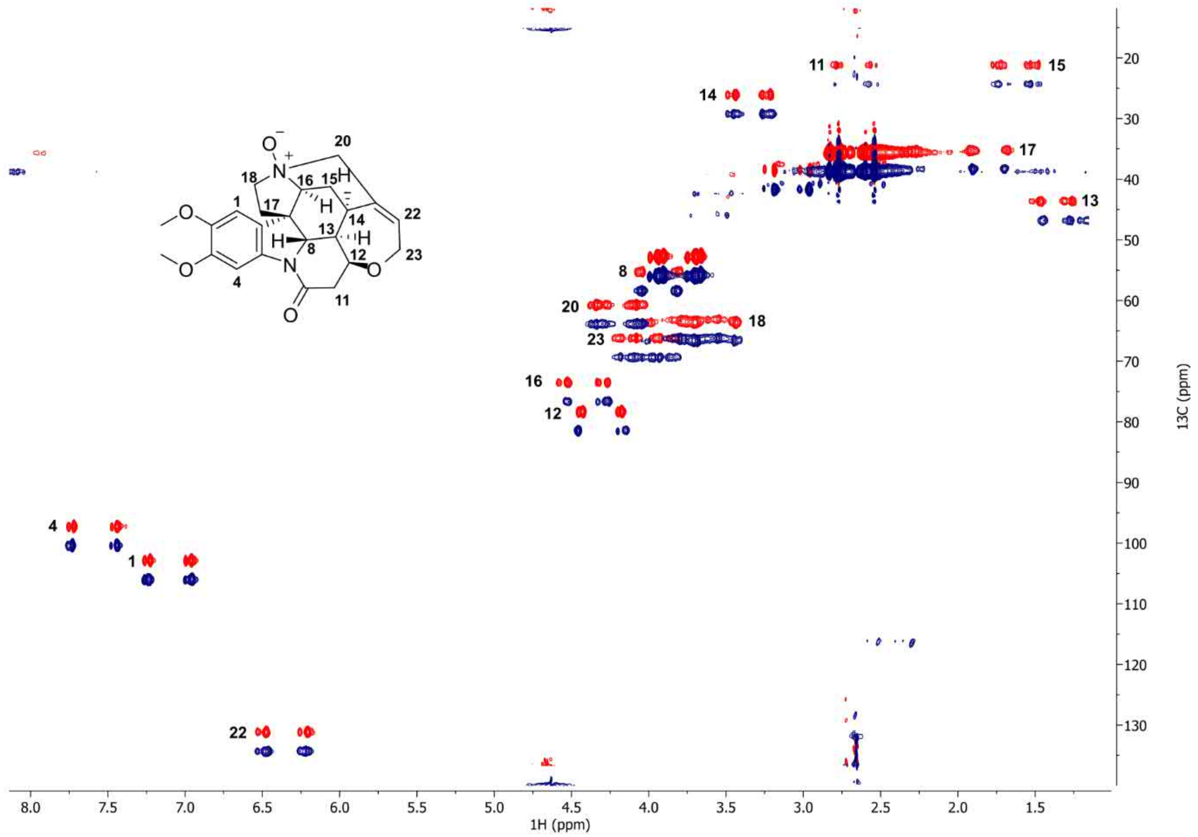

Figure S2. Overlaid f2-coupled $^{1}H,^{13}C$-HSQC spectra of brucine *N*-oxide (D_2O, 600 MHz) in weakly (red) and strongly aligned conditions (blue). The strongly aligned spectrum is shifted by 3 ppm in the f1 dimension for better visibility.

Table S6. RDC values *D* for brucine *N*-oxide in D_2O determined as the difference between total coupling constants *T* in weakly and strongly aligned conditions.

Assignment	T_{weak} (Hz)	T_{strong} (Hz)	*D* (Hz)
1	160.9	167.4	6.5
4	166.0	174.0	8.0
8	133.1	134.6	1.5
11	132.3	159.5	27.3
12	153.7	183.1	29.5
13	119.3	105.7	-13.6
14	135.3	139.6	4.4
15	147.8	158.9	11.2
16	154.8	163.5	8.7
17	132.4	128.3	-4.1
18	158.9	157.7	-1.2
20	154.4	180.4	26.0
22	160.5	148.9	-11.6
23	160.9	150.6	-10.3

2 NMR spectroscopy

^{1}H, ^{13}C, COSY, TOCSY, HSQC and NOESY spectra were recorded with excitation sculpting water suppression on a 800 MHz Bruker Avance III HD spectrometer equipped with a cryogenic TXO probe (CRPHe TR- 13 C/ 15 N/ 1 H 5mm-Z). Solution samples were prepared by dissolving lyophilized compound (3 mg) in a 9:1 mixture of H_2O:D_2O (450 µL).

A series of seven NOESY (noesyesgpph) spectra with mixing times from 100 to 700 ms (100 ms increments) was recorded for each peptide. The NOESY spectra were recorded with a relaxation delay of 2.5 s, 16 transients, 8192 points in f2 and 512 increments in f1. All parameters were kept identical throughout the series.

Residual dipolar couplings were extracted from f1-coupled, f2-decoupled ^{1}H,^{13}C-HSQC spectra recorded on a 600 MHz Bruker Avance Neo spectrometer equipped with a cryogenic TCI probe (CRPHe TR-1H & 19F/13C/15N 5 mm-EZ). A modified perfect_clip_hsqc pulse sequence[6] was used with a relaxation delay of 2.0 s, 16 transients, 512 points in f2 and 2048 increments in f1, with 16 transients in isotropic and 32 transients in anisotropic conditions. Lyophilized compound (9 mg) was dissolved in D_2O (500 µL). A poly-(4-acryloylmorpholine-*co*-acrylonitrile) gel piece of 30 mm length was added to the peptide solution and allowed to swell for at least 8 h. Weakly aligned reference data was acquired in the swollen gel. Strong alignment was achieved by compressing the gel to about 70% of its swollen length using a compression gel device by New Era Enterprise, Inc.. ^{2}H NMR spectra were recorded to observe the residual quadrupolar coupling of the solvent signal (unresolved in weakly aligned conditions, 11.7 – 15.6 Hz in strongly aligned conditions) which confirms molecular alignment in the magnetic field.

Chemical shifts are reported in parts per million (ppm, δ scale). NMR spectra were processed with MestReNova (v. 15.0.1-35756). The original FIDs as well as the processed spectra are deposited in the open access repository Zenodo and are available free of charge on doi:10.5281/zenodo.16900969.

2.1 Chemical shift assignments

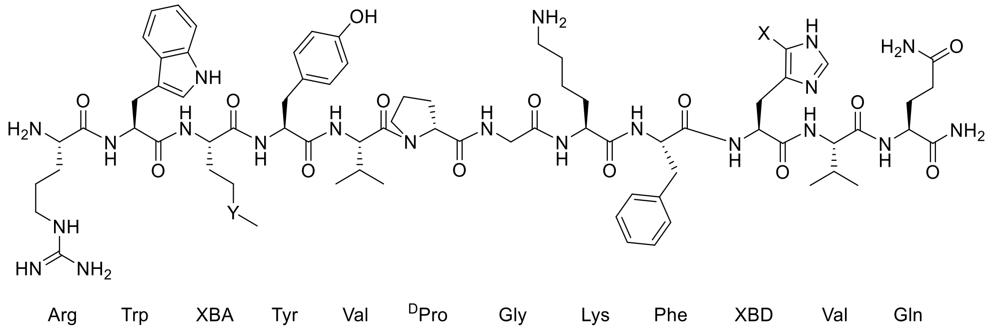

1: X=H, Y=CH_2, **2:** X=Br, Y=O, **3:** X=Br, Y=S, **4:** X=I, Y=O, **5:** X=I, Y=S

Figure S3. Structure of compounds **1-5**.

Table S7. ^{1}H NMR chemical shift assignment of compound **1** in H_2O:D_2O (9:1).

Residue	Hα	Hβ	Hγ	Hδ	Hε	H1	H2	H3	H4	H5	H6	H7	NH[c]
Arg-1	3.86	1.76	1.46	3.06									-
Trp-2	4.90	3.02				10.03[a]	7.07		7.35	6.97	7.13	7.37	7.34
Nle-3	4.38	1.50, 1.59	1.05	1.12	0.69								8.55
Tyr-4	4.78	2.62, 2.81					6.70	6.58		6.58	6.70		8.24
Val-5	4.40	1.86	0.76, 0.79										8.45
D-Pro-6	4.28	1.84, 2.22	1.91	3.58, 3.62									
Gly-7	3.63, 3.91												7.95
Lys-8	4.39	1.59, 1.63	1.18, 1.23	1.54, 1.62	2.85	8.05[b]							7.69
Phe-9	4.61	2.73, 2.82					6.90	7.04	6.98	7.04	6.90		8.37
His-10	4.62	2.82, 2.97					7.58	6.86[a]		7.03			8.61
Val-11	3.89	1.77	0.68, 0.73										8.20
Gln-12	4.19	1.77, 1.94	2.19										8.28

[a]NH [b]NH_2 [c]backbone

Table S8. ^{1}H NMR chemical shift assignment of compound **2** in H_2O:D_2O (9:1).

Residue	Hα	Hβ	Hγ	Hδ	Hε	H1	H2	H3	H4	H5	H6	H7	NH[c]
Arg-1	3.91	1.68	1.41	3.03	7.05[a]								7.68
Trp-2	4.80	2.99				10.03[a]	7.08		7.34	6.98	7.14	7.37	7.39
Hse(Me)-3	4.47	1.62, 1.74	3.23		3.17								8.62
Tyr-4	4.93	2.67, 2.84					6.78	6.57		6.57	6.78		8.21
Val-5	4.39	1.94	0.82, 0.83										8.50
D-Pro-6	4.29	1.87, 2.22	1.95	3.61, 3.67									
Gly-7	3.73, 3.89												8.05
Lys-8	4.45	1.65, 1.68	1.22, 1.27	1.56	2.86	7.92[a]							7.76
Phe-9	4.87	2.64, 2.72					6.83	6.98	7.07	6.98	6.83		8.23
His(Br)-10	4.65	2.82, 3.01					7.49						8.43
Val-11	3.94	1.77	0.67, 0.71										8.21
Gln-12	4.19	1.81, 1.92	2.22										8.34

[a]NH [b]NH_2 [c]backbone

Table S9. ^{1}H NMR chemical shift assignment of compound **3** in H_2O:D_2O (9:1).

Residue	Hα	Hβ	Hγ	Hδ	Hε	H1	H2	H3	H4	H5	H6	H7	NH[c]
Arg-1	3.79	1.72	1.44	3.06	7.05[a]								7.69
Trp-2	4.86	2.99				10.04	7.09		7.30	6.95	7.14	7.37	7.38
Met-3	4.53	1.67, 1.81	2.27		1.94								8.73
Tyr-4	4.98	1.67, 2.83					6.74	6.57		6.57	6.74		8.26
Val-5	4.41	1.94	0.80, 0.81										8.52
D-Pro-6	4.27	1.87, 2.26	1.95	3.61, 3.67									
Gly-7	3.70, 3.92												8.04
Lys-8	4.48	1.65, 1.68	1.27	1.67	2.88	7.92[b]							7.74
Phe-9	4.90	2.65, 2.74					6.82	6.96	6.91	6.96	6.82		8.28
His(Br)-10	4.67	2.80, 3.02					7.51						8.50
Val-11	3.95	1.72	0.63, 0.68										8.20
Gln-12	4.19	1.76, 1.67	2.22										8.31

[a]NH [b]NH_2 [c]backbone

Table S10. ^{1}H NMR chemical shift assignment of compound **4** in H_2O:D_2O (9:1).

Residue	Hα	Hβ	Hγ	Hδ	Hε	H1	H2	H3	H4	H5	H6	H7	NH[c]
Arg-1	3.85	1.68	1.47	3.06	7.06[a]								7.69
Trp-2	4.80	2.98				10.03[a]	7.08		7.34	6.97	7.14	7.37	7.39
Hse(Me)-3	4.47	1.62, 1.71	3.23		3.17								8.63
Tyr-4	4.93	2.69, 2.84					6.79	6.57		6.57	6.79		8.20
Val-5	4.40	1.94	0.82, 0.83										8.51
D-Pro-6	4.29	1.87, 2.22	1.95	3.61, 3.67									
Gly-7	3.75, 3.89												8.09
Lys-8	4.45	1.65, 1.68	1.22, 1.27	1.54	2.86	7.92[b]							7.77
Phe-9	4.87	2.64, 2.72					6.82	6.96	6.96	6.96	6.82		8.23
His(I)-10	4.65	2.82, 2.99					7.58						8.42
Val-11	3.94	1.77	0.67, 0.71										8.21
Gln-12	4.19	1.81, 1.92	2.22										8.33

[a]NH [b]NH_2 [c]backbone

Table S11. ^{1}H NMR chemical shift assignment of compound **5** in H_2O:D_2O (9:1).

Residue	Hα	Hβ	Hγ	Hδ	Hε	H1	H2	H3	H4	H5	H6	H7	NH[c]
Arg-1	3.94	1.79, 1.83	1.50	3.08	7.07[a]								7.69
Trp-2	4.88	2.99				10.04[a]	7.10		7.30	6.95	7.14	7.38	8.63
Met-3	4.52	1.71, 1.82	2.28		1.94								8.73
Tyr-4	4.97	2.68, 2.83					6.77	6.52		6.52	6.77		8.26
Val-5	4.43	1.94	0.80, 0.81										8.54
D-Pro-6	4.28	1.87, 2.25	1.95	3.62, 3.69									
Gly-7	3.70, 3.90												8.10
Lys-8	4.48	1.64, 1.70	1.24, 1.29	1.57	2.88	7.92[a]							7.75
Phe-9	4.85	2.66, 2.74					6.82	6.96	6.92	6.96	6.82		8.29
His(I)-10	4.67	2.81, 2.98					7.51						8.48
Val-11	3.96	1.72	0.63, 0.68										8.14
Gln-12	4.18	1.79, 1.92	2.22										8.28

[a]NH [b]NH_2 [c]backbone

2.2 Interproton distances derived from NOE build-ups

A series of seven NOESY spectra was acquired for each compound to extract quantitative interproton distances. The intensities of corresponding crosspeaks (cp) and diagonal peaks (dp) were normalized according to the peak amplitude normalization for improved cross relaxation (PANIC) method:[7]

$$I_{\mathrm{norm}} = \sqrt{\frac{\mathrm{cp_{ab}} \cdot \mathrm{cp_{ba}}}{\mathrm{dp_a} \cdot \mathrm{dp_b}}},$$

where I_{norm} is the normalized intensity for the NOE between protons a and b. A series of at least five normalized intensities from increasing mixing times following a linear relationship with $R^2 \geq 0.95$ were used to determine the NOE build-up rate σ_{ab} for each proton-proton pair. All interproton distances r_{ab} were deduced with the aid of the invariable reference distance r_{ref} = 2.54 Å between the ortho protons of tyrosine:[8]

$$r_{\mathrm{ab}} = r_{\mathrm{ref}} \cdot \left(\frac{\sigma_{\mathrm{ref}}}{\sigma_{\mathrm{ab}}}\right)^{-6}.$$

Error estimates were adjusted to the size of the distance and are given in Table S12. Longer distances arise from lower peak intensities inheriting a larger experimental uncertainty.[9] Interproton distances which were used in the fitting procedures for compounds **1-5** are listed in Tables S13-S17.

Table S12. Tiered error estimates for NOE-derived interproton distances.

r_{ab}	ε_{NOE}
< 2.5 Å	0.1 Å
2.5 – 3.5 Å	0.2 Å
> 3.5 Å	0.3 Å

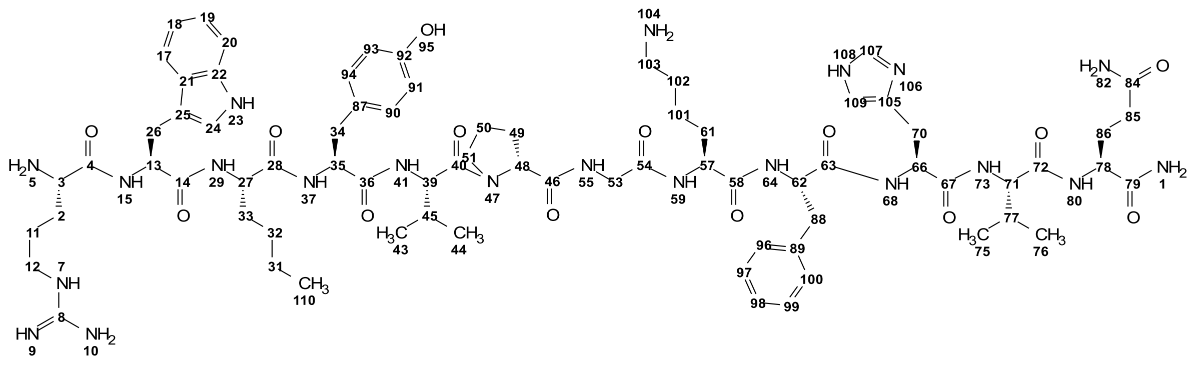

Figure S4. Structure and atom numbering for compound **1.**

Table S13. NOE-derived interproton distances for compound **1**. Ambiguous assignments are separated by a comma.

#	Type a	Type b	Proton a	Proton b	σ_{ab}	R^2	Distance r_{ab} (Å)
1	Hα,Hδ	Hα	51,53	48	8.07E-06	0.960	3.92
2	Hα,Hδ	Hα	51,53	27,39,57	7.87E-05	0.996	2.68
3	Hα,Hδ	NH	51,53	59	1.87E-05	0.963	3.41
4	Hα,Hδ	NH	51,53	55	7.27E-05	0.967	2.71
5	Hα,Hδ	NH	51,53	41	7.03E-06	0.962	4.01
6	Hα	NH	3,53,71	59	1.86E-05	0.992	3.41
7	Hα	NH	3,53,71	55	3.40E-05	0.966	3.08
8	Hα	NH	3,53,71	73	2.30E-05	0.972	3.29
9	Hα	NH	3,53,71	80	1.04E-04	0.995	2.56
10	Hα	NH	3,53,71	29	5.29E-06	0.960	4.20
11	Hα	NH	78	80	2.35E-05	0.969	3.28
12	Hα	NH	48	55	7.93E-05	0.962	2.68
13	Hα	NH	27,39,57	59	1.73E-05	0.974	3.45
14	Hα	NH	27,39,57	37	9.31E-05	0.998	2.60
15	Hα	NH	27,39,57	64	3.79E-05	0.964	3.03
16	Hα	NH	27,39,57	41	2.12E-05	0.962	3.33
17	Hα	NH	27,39,57	29	1.90E-05	0.974	3.40
18	Hα	NH	13	29	2.31E-04	0.996	2.24
19	NH	NH	59	41	2.16E-05	0.965	3.32
20	NH	NH	55	41	5.32E-06	0.986	4.20
21	NH	NH	37	29	2.06E-05	0.951	3.35
22	Hβ	Hα,Hδ	45,49	51,53	2.71E-05	0.996	3.20
23	Hβ	Hα	45,49	48	2.56E-05	0.961	3.23
24	Hβ	Hα	45,49	27,39,57	1.02E-05	0.960	3.76
25	Hβ	Hα,Hδ	34	51,53	2.43E-06	0.972	4.78
26	Hγ	Hβ	32	70	3.64E-06	0.984	4.47
27	Hβ	Har	70	19	1.85E-06	0.945	5.00
28	Hβ	Hα,Hγ	26	3,53,71	5.57E-06	0.997	4.16
29	Hα,Hδ	Hα	33,102	27,39,57	8.56E-06	0.967	3.88
30	Hβ	Har	70	24,97,99,109	4.31E-06	0.987	4.35
31	Hβ	HN	70	78	1.08E-05	0.959	3.73
Ref.	Har	Har	91,93	90,94	1.08E-04	0.992	2.54

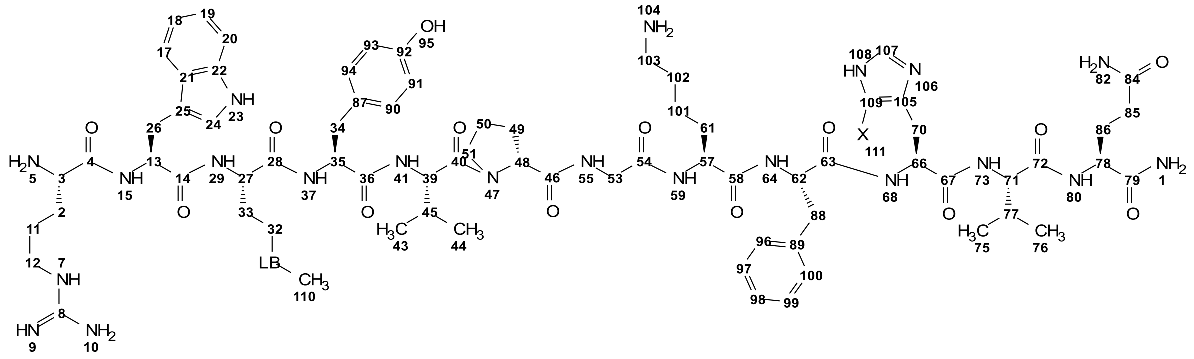

Figure S5. Structure and atom numbering for compounds **2-5**. X = Br, I; LB = O, S.

Table S14. NOE-derived interproton distances for compound **2**. Ambiguous assignments are separated by a comma.

#	Type a	Type b	Proton a	Proton b	σ_{ab}	R^2	Distance r_{ab} (Å)
1	Hα	NH	71,3,53	37,64,73	1.06E-05	0.989	3.67
2	Hα	NH	48	59	6.77E-06	0.960	3.96
3	Hα	NH	39	41	4.33E-05	0.964	2.90
4	Hα	NH	27,57	59	3.52E-05	0.953	3.01
5	Hα	NH	35	37,64,73	3.17E-05	0.991	3.06
6	Hα	NH	35	41	2.26E-04	0.995	2.20
7	NH	NH	59	41	2.78E-05	0.995	3.13
8	NH	NH	37,64,73	41	1.16E-05	0.954	3.62
9	Hβ	NH	33,61,2	59	3.13E-05	0.989	3.07
10	Hβ	NH	33,61,2	41	2.01E-06	0.959	4.84
11	Hβ	NH	88	68	2.91E-05	0.957	3.10
12	Hβ,Hε	NH	34,70,103	41	1.17E-05	0.993	3.61
13	Hδ	Hα	51	48	1.15E-05	0.962	3.62
14	Hδ	Hα	51	39	1.24E-04	0.998	2.44
15	Hδ	NH	51	41	5.19E-06	0.969	4.14
16	Hδ	Hα	51	48	7.54E-06	0.963	3.89
17	Hδ	Hα	51	39	1.46E-04	0.997	2.37
18	Hδ	NH	51	41	4.27E-06	0.967	4.27
19	Hγ	Har	32	107	2.55E-06	0.986	4.66
Ref.	Har	Har	91,93	90,94	9.68E-05	0.980	2.54

Table S15. NOE-derived interproton distances for compound **3**. Ambiguous assignments are separated by a comma.

#	Type a	Type b	Proton a	Proton b	σ_{ab}	R^2	Distance r_{ab} (Å)
1	Hα	NH	53	59	3.26E-05	0.997	3.16
2	Hα	NH	71	73	3.79E-05	0.990	3.08
3	Hα	NH	71	80	1.38E-04	1.000	2.48
4	Hα	NH	71	29	1.48E-05	0.955	3.61
5	Hα	NH	48	59	6.68E-06	0.977	4.12
6	Hα	NH	39	41	3.28E-05	0.989	3.16
7	Hα	NH	57	64	1.36E-04	0.992	2.49
8	Hα	NH	62	64	4.32E-05	0.981	3.02
9	Hα	NH	62	68	2.16E-04	0.954	2.31
10	Hα	NH	35	37	4.45E-05	0.989	3.00
11	Hα	NH	35	41	2.16E-04	0.999	2.31
12	NH	NH	59	64	1.25E-05	0.965	3.71
13	NH	NH	59	41	2.70E-05	0.997	3.26
14	NH	NH	37	29	1.80E-05	0.967	3.49
15	NH	NH	64	41	6.20E-06	0.955	4.17
16	NH	NH	68	29	2.32E-05	0.956	3.34
17	Hβ	NH	49	55	8.56E-06	0.987	3.95
18	Hβ	Hα	88	62	8.43E-05	0.964	2.70
19	Hβ	NH	88	64	5.39E-05	0.997	2.91
20	Hβ	NH	88	68	2.44E-05	0.988	3.32
21	Hβ	Hα	26	71	6.43E-06	0.993	4.14
22	Hβ	Hα	70	71	3.64E-06	0.973	4.55
23	Hβ	NH	70	73	2.01E-05	0.967	3.43
24	Hδ	NH	51	41	4.65E-06	0.982	4.37
Ref.	Har	Har	91,93	90,94	1.21E-04	0.986	2.54

Table S16. NOE-derived interproton distances for compound **4**. Ambiguous assignments are separated by a comma.

#	Type a	Type b	Proton a	Proton b	σ_{ab}	R^2	Distance r_{ab} (Å)
1	Hα	NH	53	59	3.97E-05	0.996	3.03
2	Hα	NH	53	55	1.20E-04	0.997	2.52
3	Hα	Hα	53	48	4.34E-06	0.974	4.38
4	Hα	NH	53	59	3.59E-05	1.000	3.08
5	Hα	NH	53	55	1.01E-04	0.995	2.59
6	Hα	NH	71	80	1.02E-04	0.991	2.58
7	Hα	NH	71	29	8.09E-06	0.954	3.94
8	Hα	NH	48	59	6.07E-06	0.988	4.14
9	Hα	NH	48	55	8.66E-05	0.993	2.66
10	Hα	NH	48	41	1.34E-06	0.999	5.32
11	Hα	NH	39	41	3.32E-05	0.984	3.12
12	Hα	NH	27,57	68	4.47E-06	0.973	4.35
13	Hα	NH	27,57	29	1.66E-05	0.950	3.50
14	Hα	NH	62	68	1.79E-04	0.968	2.35
15	Hα	NH	35	64,73,37	2.66E-05	0.980	3.24
16	Hα	NH	35	41	2.25E-04	0.999	2.27
17	NH	NH	59	64,73,37	1.25E-05	0.998	3.67
18	NH	NH	59	41	2.29E-05	0.997	3.32
19	NH	NH	64,73,37	68	9.94E-06	0.962	3.81
20	NH	NH	64,73,37	41	8.37E-06	0.989	3.92
21	NH	NH	64,73,37	29	1.31E-05	0.991	3.64
22	NH	NH	68	29	1.73E-05	0.968	3.48
23	Hβ	NH	33	64,73,37	9.38E-06	0.974	3.85
24	Hβ	Hα	77	78	2.94E-06	0.983	4.67
25	Hβ	NH	77	64,73,37	5.74E-06	0.999	4.18
26	Hβ	Hα	86	3	1.50E-06	0.980	5.23
27	Hβ	Hα	49	53	7.27E-07	0.955	5.89
28	Hβ	NH	49	55	8.96E-06	0.960	3.88
29	Hβ	Hα	88	62	9.93E-05	0.954	2.60
30	Hβ	NH	88	68	2.95E-05	0.995	3.18
31	Hβ	NH	24	59	8.17E-07	0.995	5.78
32	Hβ	Hα	88	62	8.88E-05	0.951	2.65
33	Hβ	NH	88	64,73,37	4.06E-05	0.999	3.01
34	Hβ	NH	88	68	2.80E-05	0.983	3.21
35	Hδ	NH	51	55	9.10E-06	0.981	3.87
36	Hδ	Hα	51	48	9.10E-06	0.994	3.87
37	Hδ	Hα	51	39	1.49E-04	1.000	2.43
38	Hδ	NH	51	41	5.61E-06	0.972	4.19
39	Hβ	Hγ	33	32	2.63E-05	0.958	3.24
40	Hβ	NH	33	29	2.45E-05	1.000	3.28
41	Hβ	NH	70,26	68	2.53E-05	0.995	3.26
42	Hγ	Har	32	107	4.20E-06	1.000	4.40
43	Hγ	NH	32	29	1.00E-05	0.962	3.81
Ref.	Har	Har	91,93	90,94	1.13E-04	0.992	2.54

Table S17. NOE-derived interproton distances for compound **5**. Ambiguous assignments are separated by a comma.

#	Type a	Type b	Proton a	Proton b	σ_{ab}	R^2	Distance r_{ab} (Å)
1	Hα	HN	53	59	2.98E-05	0.994	3.15
2	Hα	HN	53	55	8.96E-05	0.998	2.62
3	Hα	HN	53	59	2.77E-05	0.997	3.19
4	Hα	HN	53	55	7.09E-05	0.999	2.73
5	Hα	Hα	71	13	2.31E-05	0.948	3.29
6	Hα	HN	71	73	3.24E-05	0.974	3.11
7	Hα	HN	71	29	8.88E-06	0.980	3.86
8	Hα	HN	48	59	6.40E-06	0.989	4.07
9	Hα	HN	39	41	4.15E-05	0.971	2.98
10	Hα	HN	57	59	4.88E-05	0.956	2.90
11	Hα	HN	13	29	1.76E-04	0.949	2.34
12	Hα	HN	35	37	3.96E-05	0.967	3.00
13	Hα	HN	35	41	2.13E-04	0.998	2.27
14	HN	HN	59	64,80	1.22E-05	0.992	3.66
15	HN	HN	59	41	2.62E-05	0.997	3.22
16	HN	HN	55	41	6.56E-06	0.966	4.05
17	HN	HN	73	68	6.68E-06	0.958	4.04
18	HN	HN	37	41	9.68E-06	0.997	3.80
19	HN	HN	37	29	1.53E-05	0.995	3.52
20	HN	HN	68	29	3.02E-05	0.998	3.14
21	Hβ	HN	61	41	3.54E-06	0.973	4.49
22	Hβ	HN	49	55	1.03E-05	0.986	3.76
23	Hδ	HN	51	41	5.21E-06	0.990	4.21
24	Hδ	Hα	51	48	1.03E-05	0.967	3.76
25	Hδ	HN	51	41	5.56E-06	0.966	4.17
26	Hβ	HN	33,61,77	29	1.98E-05	0.998	3.37
27	Hβ	HN	2,33	37	1.75E-05	0.998	3.44
28	Hβ	HN	2,33	29	1.57E-05	0.995	3.50
29	Hβ,Hγ	HN	32,49,85	37	2.77E-06	0.975	4.68
30	Hβ,Hγ	HN	32,49,85	29	9.42E-06	0.998	3.82
31	Hβ	HN	24,70	68	1.38E-05	0.980	3.58
32	Hβ	HN	26,70	68	2.28E-05	0.997	3.30
Ref.	Har	Har	91,93	90,94	1.09E-04	0.981	2.54

2.3 Residual dipolar couplings (RDCs)

The residual dipolar coupling constant D_{CH} was calculated as the difference between the total coupling constants T observed in strongly and weakly aligned conditions:

$$D_{\mathrm{CH}} = T_{\mathrm{strong}} - T_{\mathrm{weak}}$$

For CH_2 and CH_3 groups, individual coupling constants for each C-H pair cannot be determined from f1-coupled HSQC spectra. The splitting between outer lines was therefore measured and divided by 2 and 3 respectively to obtain the averaged total coupling constant. The error ε_{RDC} on measured RDCs is calculated from the average of the linewidths at half maximum height $\Delta\nu_{1/2}$ of crosspeaks A and B, as well as the signal to noise ratio S/N averaged over both crosspeaks:

$$\varepsilon_{\mathrm{RDC}} = \frac{\Delta\nu_{1/2}(\mathrm{A}) + \Delta\nu_{1/2}(\mathrm{B})}{2 \cdot S/N}$$

which is given in Hz. Measured RDC values are listed in Tables S18-S21. The same atom numbering is used as for the NOE data (see Figure S5).

Table S18. RDC values *D* for compound **2** determined as the difference between total coupling constants *T* in weakly and strongly aligned conditions. Measured splittings from CH_n groups were divided by n.

#	Residue	Type	Assignment	T_{weak} (Hz)	T_{strong} (Hz)	*D* (Hz)	ε_{RDC} (Hz)
1	Arg-1	Cα-H	3	147.0	147.5	0.5	1.2
2	Trp-2	Cα-H	13	142.7	144.1	1.4	1.6
3	Hse(Me)-3	Cα-H	27	141.2	142.0	0.8	2.0
4		Cβ-H_2	33	129.1	128.6	-0.6	2.0
5		Cγ-H_2	32	143.1	143.5	0.5	1.1
6		Cε-H_3	110	141.4	135.5	-5.9	0.9
7	Tyr-4	Cα-H	35	143.8	142.7	-1.2	2.0
8	Val-5	Cα-H	39	139.3	141.5	2.2	2.9
9	D-Pro-6	Cα-H	48	148.7	148.9	0.2	1.2
10	Lys-8	Cα-H	57	141.3	141.6	0.3	2.0
11	Phe-9	Cα-H	62	143.9	143.9	-0.1	0.9
12	His(Br)-10	Cβ-H_2	70	132.8	133.7	1.0	6.1
13		Cε-H	107	214.0	214.2	0.3	6.3
14	Val-11	Cα-H	71	143.0	143.9	0.9	2.2
15	Gln-12	Cα-H	78	142.7	142.8	0.1	1.9

Table S19. RDC values *D* for compound **3** determined as the difference between total coupling constants *T* in weakly and strongly aligned conditions. Measured splittings from C-H_n groups were divided by n.

#	Residue	Type	Assignment	T_{weak} (Hz)	T_{strong} (Hz)	*D* (Hz)	ε_{RDC} (Hz)
1	Arg-1	Cα-H	3	147.6	147.2	-0.4	1.1
2	Trp-2	Cα-H	13	143.3	142.7	-0.6	1.1
3	Met-3	Cα-H	27	141.4	141.5	0.1	1.6
4		Cβ-H_2	33	129.7	133.1	3.3	1.8
5		Cγ-H_2	32	139.2	139.1	-0.1	1.1
6		Cε-H_3	110	139.0	125.4	-13.7	0.9
7	Tyr-4	Cα-H	35	143.7	144.5	0.8	1.4
8	Val-5	Cα-H	39	140.7	141.5	0.8	1.4
9	D-Pro-6	Cα-H	48	149.2	150.5	1.3	1.4
10	Lys-8	Cα-H	57	142.5	141.5	-1.0	1.5
11	Phe-9	Cα-H	62	145.5	145.3	-0.3	1.1
12	His(Br)-10	Cα-H	66	143.9	144.9	1.0	1.7
13		Cβ-H_2	70	133.1	133.1	-0.1	2.1
14		Cε-H	107	157.8	154.9	-2.9	4.1
15	Val-11	Cα-H	71	142.5	143.2	0.8	2.0
16	Gln-12	Cα-H	78	142.9	143.9	0.9	1.5

Table S20. RDC values *D* for compound **4** determined as the difference between total coupling constants *T* in weakly and strongly aligned conditions. Measured splittings from C-H$_n$ groups were divided by n.

#	Residue	Type	Assignment	T_{weak} (Hz)	T_{strong} (Hz)	*D* (Hz)	ε_{RDC} (Hz)
1	Arg-1	Cα-H	3	146.3	148.7	2.4	2.6
2	Trp-2	Cα-H	13	142.9	143.3	0.4	1.6
3	Hse(Me)-3	Cα-H	27	141.6	142.6	1.0	1.6
4		Cβ-H_2	33	130.1	128.3	-1.8	2.3
5		Cγ-H_2	32	144.2	141.6	-2.6	1.3
6		Cε-H_3	110	142.0	134.7	-7.4	0.1
7	Tyr-4	Cα-H	35	143.2	141.1	-2.2	1.6
8	Val-5	Cα-H	39	141.7	139.0	-2.7	2.1
9	D-Pro-6	Cα-H	48	148.8	148.7	-0.1	0.7
10	Lys-8	Cα-H	57	141.3	142.7	1.4	2.3
11	His(I)-10	Cα-H	66	143.5	141.8	-1.7	0.8
12		Cβ-H_2	70	134.3	132.5	-1.8	3.6
13		Cε-H	107	160.1	147.2	-12.9	3.1
14	Val-11	Cα-H	71	142.4	143.5	1.1	2.4
15	Gln-12	Cα-H	78	143.5	143.8	0.3	1.4

Table S21. RDC values *D* for compound **5** determined as the difference between total coupling constants *T* in weakly and strongly aligned conditions. Measured splittings from C-H$_n$ groups were divided by n.

#	Residue	Type	Assignment	T_{weak} (Hz)	T_{strong} (Hz)	*D* (Hz)	ε_{RDC} (Hz)
1	Arg-1	Cα-H	3	148.1	147.2	-0.9	5.3
2	Trp-2	Cα-H	13	145.1	142.7	-2.4	4.0
3	Hse(Me)-3	Cα-H	27	145.1	142.7	-2.4	3.3
4		Cβ-H_2	33	125.9	128.3	2.5	5.0
5		Cγ-H_2	32	139.7	140.3	0.6	2.3
6		Cε-H_3	110	138.0	125.2	-12.9	0.6
7	Tyr-4	Cα-H	35	145.1	143.8	-1.2	4.3
8	Val-5	Cα-H	39	142.7	139.0	-3.6	4.9
9	D-Pro-6	Cα-H	48	147.5	148.7	1.2	3.5
10	Lys-8	Cα-H	57	141.5	144.0	2.5	5.6
11	His(I)-10	Cα-H	66	142.7	146.3	3.6	3.0
12		Cβ-H_2	70	129.5	130.5	1.0	4.7
13		Cε-H	107	149.8	155.4	5.7	2.1
14	Val-11	Cα-H	71	142.7	145.6	2.9	4.4
15	Gln-12	Cα-H	78	148.1	152.2	4.2	4.4

2.4 Amide temperature coefficients

^1H NMR spectra were acquired for compound **5** on a 600 MHz Bruker Avance Neo spectrometer equipped with a cryogenic TCI probe (CRPHe TR-^1H & $^{19}F/^{13}C/^{15}N$ 5 mm-EZ) in a 9:1 mixture of H_2O:D_2O at temperatures ranging from 5 °C/278 K to 50 °C/323 K. The spectra were referenced against residual CD_2HCN in a capillary. Temperature dependence of the reference was accounted for by correcting chemical shifts using the temperature coefficient $\Delta\delta/\Delta T$ = 2.3 ppb K^{-1} for acetonitrile.[10] The chemical shifts of all amide proton signals showed a linear temperature dependence with $R^2 > 0.99$. Chemical shifts and amide proton temperature coefficients are listed in Table S22. For amide protons pointing towards the opposing peptide strand, temperature coefficients become less negative when moving from the terminal residues towards the β-turn (see Figures S6 and S7). This trend is consistent with an increasing likelihood of intramolecular cross-strand hydrogen bond formation, which reduces thermal sensitivity of the amide proton environment. In contrast, amide protons directed away from the opposing strand do not display a comparable pattern, suggesting limited or no participation in intramolecular hydrogen bonding.

Table S22. Amide proton temperature coefficients $\Delta\delta_{NH}/\Delta T$ (ppb K^{-1}) for compound **5** in H_2O:D_2O (9:1).

Residue	Arg-1	Trp-2	Met-3	Tyr-4	Val-5	Gly-7	Lys-8	Phe-9	His-10	Val-11	Gln-12
5 °C	7.105	8.026	8.079	7.627	7.866	7.542	7.033	7.658	7.811	7.582	7.642
10 °C	7.060	7.984	8.054	7.596	7.847	7.487	7.022	7.622	7.790	7.530	7.613
15 °C	7.021	7.943	8.028	7.564	7.828	7.435	7.012	7.587	7.770	7.478	7.587
20 °C	6.975	7.903	7.999	7.531	7.807	7.384	7.005	7.555	7.748	7.426	7.555
25 °C	6.935	7.866	7.969	7.498	7.785	7.336	6.997	7.527	7.715	7.377	7.524
30 °C	6.897	7.840	7.935	7.463	7.761	7.289	6.989	7.503	7.701	7.330	7.493
35 °C	6.860		7.900	7.427	7.736	7.244	6.982	7.464	7.675	7.284	7.464
40 °C	6.823		7.862	7.390	7.708	7.202	6.975	7.435	7.647	7.238	7.435
45 °C	6.785		7.822	7.352	7.679	7.162	6.968	7.402	7.618	7.195	7.407
50 °C	6.754		7.780	7.313	7.648	7.124	6.961	7.366	7.587	7.153	7.382
$\Delta\delta_{NH}/\Delta T$	-7.8	-7.6	-6.6	-7.0	-4.8	-9.3	-1.6	-6.3	-4.9	-9.5	-5.9
R^2	0.998	0.995	0.993	0.998	0.993	0.997	0.995	0.999	0.995	0.999	0.999

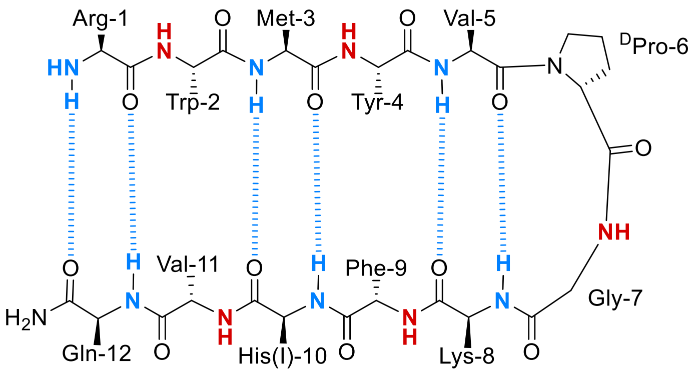

Figure S6. Structure of compound **5** with solvent-exposed amides highlighted in red and amides potentially involved in cross-strand hydrogen bonding in blue.

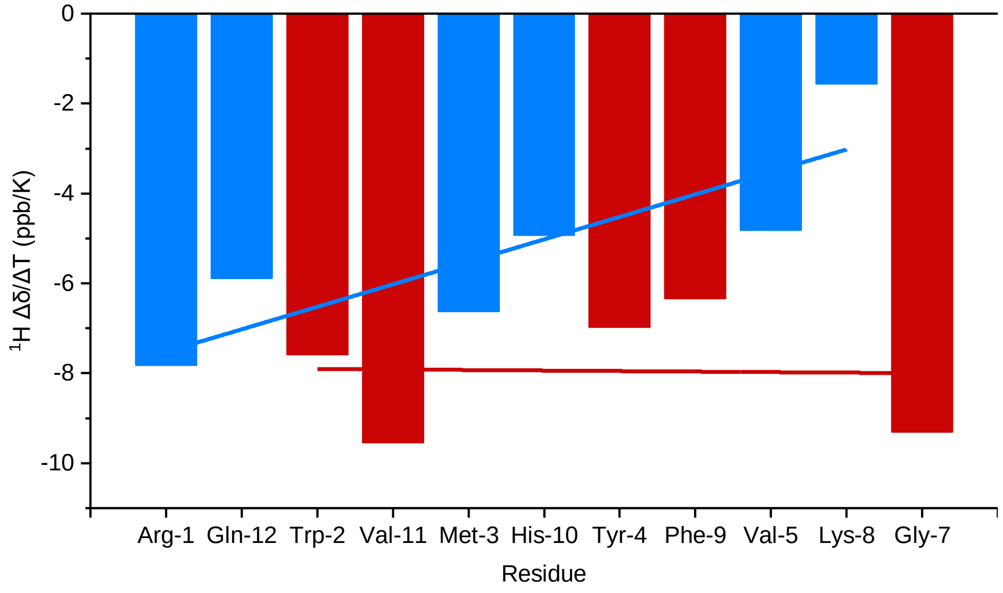

Figure S7. Amide temperature coefficients $\Delta\delta_{NH}/\Delta T$ (ppb K^{-1}) for compound **5** in H_2O:D_2O (9:1). Residues are sorted from termini (left) to β-turn (right). Solvent-exposed amides are marked in red, while amides potentially involved in cross-strand hydrogen bonding are marked in blue.

2.5 Titration of diethyl ether to 4-bromo-1*H*-imidazole

To investigate whether a intermolecular weak halogen bond is detectable through chemical shift changes in dilute polar solution, diethyl ether was titrated into a 4 mM solution of 4-bromo-1*H*-imidazole in CD_3OD. ^1H and ^{13}C chemical shifts were followed after addition of 0.5 to up to 20 equivalents of diethyl ether. ^1H NMR spectra are shown in Figure 1 of the main text and ^{13}C NMR spectra in Figure S8. No significant chemical shift changes were observed (< 0.003 ppm for ^1H and < 0.03 ppm for ^{13}C).

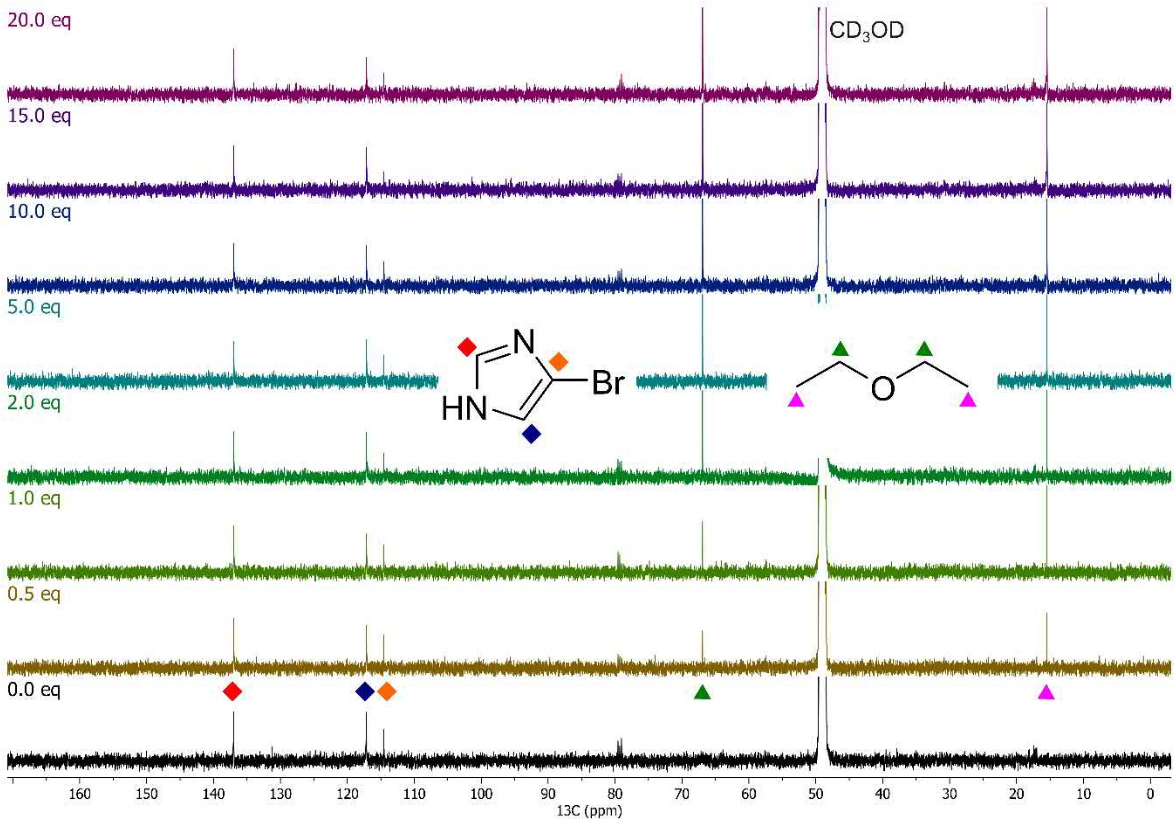

Figure S8. ^{13}C NMR titration adding equivalents of diethyl ether to 4-bromo-1*H*-imidazole in CD_3OD

3 Computational conformational sampling

3.1 Conformational sampling for backbone analysis

Monte Carlo Multiple Minimum (MCMM) torsional sampling was performed to generate theoretical conformational ensembles. The MacroModel module implemented in the Schrödinger Maestro software package (v. 13.7.125) was used. For each peptide, independent conformational searches with 50000 steps were performed utilizing OPLS4, AMBER* and MMFF force fields and implicit solvation models GB/SA for water and $CHCl_3$. Energy minimization was performed following a Polak-Ribière conjugate gradient (PRCG) algorithm with 5000 iterations. All conformers within an energy window of 42 kJ mol^{-1} from the global minimum were saved. Redundant conformers within a RMSD of 2.0 Å were eliminated.

For peptides containing halogenated His residues, additional constrained conformational searches were performed. In these additional conformation searches constraints were applied in order to mimic attractive interactions between the halogen towards a Lewis base. This is necessary due to the lack of suitable parametrization for a halogen´s σ-hole in current force fields. A distance constraint

was set to (90 ± 9) % of the van der Waals radii of halogen (Br = 1.85 Å, I = 1.98 Å) and respective Lewis base (LB) atom (O = 1.52 Å, S = 1.80 Å). The C-X ⋯LB angle was set to (180 ± 18)°. All constraints were applied with a force constant of 500 kJ mol^{-1} $Å^2$.

The output ensembles corresponding to the same compound were combined and subjected to a redundant conformer elimination (RCE) based on the comparison of backbone heavy atom coordinates with a 2.0 Å RMSD cut-off. The obtained conformational ensembles were used as input for the StereoFitter-based backbone analysis. Results are summarized in Table S24.

The conformations obtained from the conformational sampling were classified based on their overall shape and organization. The characterization criteria for β-hairpin conformations are presented in Table S23. Residues belonging to the turn region (D-Pro-6 and Gly-7) were compared to characteristic angles for type II´ β-turn formation, as determined by Jiménez.[11] The β-sheet geometry of the backbone was assessed based on interstrand distances between opposing Cα atoms and C(O)-N(H) pairs. Peptide conformers with at least four interstrand hydrogen bonds were characterized as β-hairpins. Hydrogen bonding was determined using a (C)O-(N)H distance threshold of ≤ 3 Å as described before.[12]

Table S23. Criteria used to classify conformers as β-hairpin.

Residue	ϕ	ψ	Interstand Cα-Cα (average)	Interstrand C(O)-N(H) (average)	Interstrand (C)O-(N)H
1-5, 8-12	-	-	≤ 6 Å	≤ 6 Å	at least four ≤ 3 Å
D-Pro-6	60±90°	120±90°	-	-	-
Gly-7	-80±90°	0±90°	-	-	-

Table S24. Details of MCMM conformational search.

Compound	Force field	Solvation model	XB constraints[a]	Number of conformers	
				Total	after RCE[b]
**1**	OPLS4	Water	no	294	
	OPLS4	$CHCl_3$	no	77	
	AMBER*	Water	no	104	
	AMBER*	$CHCl_3$	no	45	
	MMFF	Water	no	861	
	MMFF	$CHCl_3$	no	113	
				Σ 1494	788
**2**	OPLS4	Water	no	107	
	OPLS4	$CHCl_3$	no	65	
	AMBER*	Water	no	65	
	AMBER*	$CHCl_3$	no	24	
	MMFF	Water	no	103	
	MMFF	$CHCl_3$	no	15	
	OPLS4	Water	yes	23	
	OPLS4	$CHCl_3$	yes	19	
	AMBER*	Water	yes	75	
	AMBER*	$CHCl_3$	yes	35	

	MMFF	Water	yes	206	
	MMFF	$CHCl_3$	yes	19	
				Σ 756	348
**3**	OPLS4	Water	no	83	
	OPLS4	$CHCl_3$	no	10	
	AMBER*	Water	no	45	
	AMBER*	$CHCl_3$	no	48	
	MMFF	Water	no	100	
	MMFF	$CHCl_3$	no	31	
	OPLS4	Water	yes	51	
	OPLS4	$CHCl_3$	yes	35	
	AMBER*	Water	yes	48	
	AMBER*	$CHCl_3$	yes	69	
	MMFF	Water	yes	80	
	MMFF	$CHCl_3$	yes	101	
				Σ 701	292
**4**	OPLS4	Water	no	283	
	OPLS4	$CHCl_3$	no	31	
	AMBER*	Water	no	67	
	AMBER*	$CHCl_3$	no	63	
	MMFF	Water	no	123	
	MMFF	$CHCl_3$	no	49	
	OPLS4	Water	yes	166	
	OPLS4	$CHCl_3$	yes	127	
	AMBER*	Water	yes	11	
	AMBER*	$CHCl_3$	yes	15	
	MMFF	Water	yes	480	
	MMFF	$CHCl_3$	yes	62	
				Σ 1477	632
**5**	OPLS4	Water	no	129	
	OPLS4	$CHCl_3$	no	112	
	AMBER*	Water	no	52	
	AMBER*	$CHCl_3$	no	33	
	MMFF	Water	no	505	
	MMFF	$CHCl_3$	no	61	
	OPLS4	Water	yes	190	
	OPLS4	$CHCl_3$	yes	52	
	AMBER*	Water	yes	112	
	AMBER*	$CHCl_3$	yes	77	
	MMFF	Water	yes	462	
	MMFF	$CHCl_3$	yes	33	
				Σ 1818	791

[a] Constraints applied to the C-X ···LB angle and X ··· LB distance to mimic halogen bond geometry.

[b] Redundant conformer elimination with 2.0 Å RMSD cut-off for heavy atom coordinates.

3.2 Conformational sampling for side chain analysis

Each conformer of a halogenated peptide with a population of at least 3%, as identified by backbone analyses (see Section 4 below), was individually subjected to a second Monte Carlo Multiple Minimum (MCMM) conformational search in order to sample the orientation of the side chain containing a halogen bond acceptor (XBA) or donor (XBD) moiety. The backbone conformations were preserved by constraining ϕ-, ψ- and ω-torsional angles with a 1000 kJ mol^{-1} force constant. For each conformer, independent torsional samplings with 10000 steps were performed utilizing the AMBER* force field and implicit solvation models GB/SA for water and $CHCl_3$. Energy minimization was performed following a Polak-Ribière conjugate gradient (PRCG) algorithm with 5000 iterations. All conformers within an energy window of 42 kJ mol^{-1} from the found global minimum were saved. Redundant conformers within a RMSD of 1.5 Å were eliminated. Due to the lack of suitable parametrization for a halogen´s σ-hole, two additional constrained samplings were performed to also sample halogen-bonded geometries. The X···LB distance was constrained to (90 ± 9) % of the van der Waals radii of the halogen (Br = 1.85 Å, I = 1.98 Å) and respective Lewis base (LB) atom (O = 1.52 Å, S = 1.80 Å) with a force constant of 1000 kJ mol^{-1}. The C-X···LB angle was set to (180 ± 0)° with force constants of 100 or 1000 kJ mol^{-1} for the two constrained samplings respectively. All sampled conformers sharing the same backbone conformation were compared based on the heavy atom coordinates of the His and LB side chains. Redundant conformers within a RMSD cut-off of 1.7 Å were eliminated, ensuring that at least one halogen-bonded conformer per backbone conformation was preserved. The obtained conformational ensembles served as input for the side chain analysis.

4 Backbone analysis using NOEs and *J*-couplings

The interproton distances derived from the NOE build-up curves and $^3J_{HH}$ coupling constants extracted from ^{1}H NMR spectra were deconvoluted against the theoretical ensembles using the StereoFitter plugin (v. 1.1.6) for MestreNova (v. 15.0.1). StereoFitter was run without model selection and using the non negative linear least squares fitting algorithm. Interproton distances were prepared as shown in Section 2.2. If the intensity of a NOESY crosspeak arises from multiple NOEs due to signal overlap or the proton assignment is ambiguous, the potential contribution of each given proton-proton pair is taken into account by the Stereofitter algorithm. The pseudodistance r_{pseudo} obtained from the NOE build-up curve is then back-calculated from the individual interproton distances r_i of the selected theoretical conformers i:

$$r_{\text{pseudo}} = \sqrt[-6]{\sum_i r_i^{-6}}.$$

Consequently, r_{pseudo} will be shorter than the shortest individual distance. Due to the inverse sixth power relationship between NOE build-up rate and interproton distance, it is possible to even include overlapping protons in the experimental input, that turn out to be too distant from each other to contribute to the observed NOE. Their contribution to the back-calculated pseudodistance will be negligibly small. Dihedral angles were calculated from scalar coupling constants internally by StereoFitter using the parametrized Karplus-like equation:[13]

$$^3J_{\text{HN-H}\alpha} = 6.4\cos^2\varphi - 1.4\cos\varphi + 1.9,$$

Where $^3J_{\text{HN-H}\alpha}$ is the three-bond coupling constant between backbone amide HN and Hα, while φ is the dihedral angle between N-H and C-H_α bonds. A general error of 1 Hz was estimated for experimental coupling constants.

Since the NMR data arises from the population-weighted contribution of all conformers present in solution, the conformers existing in solution have to be selected from the input conformation pool and their populations determined. The agreement of experimental interproton distances and *J*-couplings and the corresponding back-calculated values that are population weighted averages can be expressed by:

$$\chi^2 = \sum_i \left(\frac{V_i^{\text{exp}} - V_i^{\text{calc}}}{\varepsilon_i}\right)^2,$$

Where ε_i are the uncertainties on the experimental values V_i^{exp}. The best fitting populations are found by minimizing χ^2. More details can be found in the user manual for StereoFitter.

NOE distances and *J*-couplings used in the fitting procedures for compounds **1-5** are shown in Table S25-S34 and visualized in Figures S9-S13. The fitting of this dataset yielded population-weighted conformational ensembles describing the backbone conformation of compounds **1-5**. The solution ensemble was validated by the Jackknife method, that is by iteratively removing random 10% of the distance data, and observing the conformational families do not undergo larger changes in population, and no new conformational family arises and non disappears. The result was found valid if the percentage of β-hairpin conformations did not deviate by more than 10% over 100 iterations from the fitting with complete dataset. As a second validation step, up to ±5% random noise was applied to the input distances and the populations of the major conformational families was observed to not alter by more than 10%. The populations of the backbone ensembles determined for compounds **1-5** are listed in Table S35.

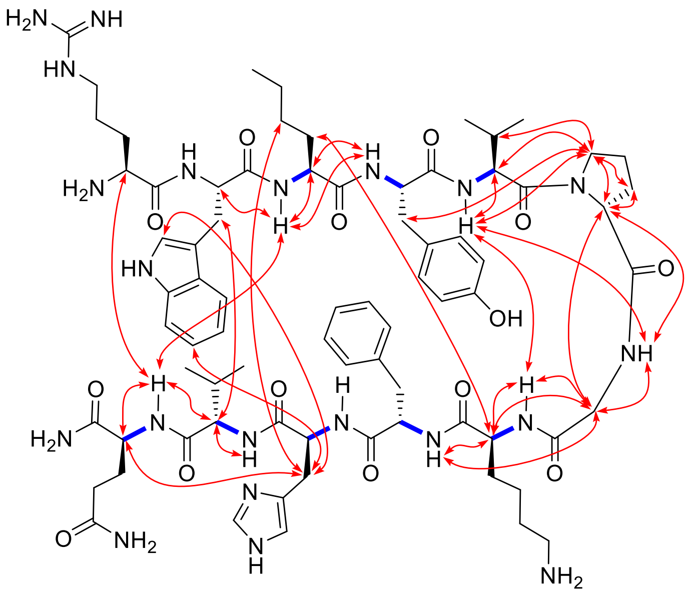

Figure S9. The key NOEs used to derive interproton distances (red) and $^{3}J_{HN\text{-}H\alpha}$ couplings (blue) used for the conformational analysis of the bakcbone of compound **1**.

Table S25. NOE-derived and back-calculated interproton distances for the backbone analysis of compound **1**.

#	Proton a	Proton b	Exp. (Å)	Calc. average (Å)	Δ (Å)
1	51,53	48	3.92	4.03	0.11
2	51,53	27,39,57	2.68	4.07	1.39*
3	51,53	59	3.41	3.40	-0.01
4	51,53	55	2.71	2.93	0.22
5	51,53	41	4.01	3.99	-0.02
6	3,53,71	59	3.41	3.42	0.01
7	3,53,71	55	3.08	2.97	-0.11
8	3,53,71	73	3.29	3.59	0.30
9	3,53,71	80	2.56	3.41	0.85
10	3,53,71	29	4.20	4.55	0.35
11	78	80	3.28	2.94	-0.34
12	48	55	2.68	2.57	-0.11
13	27,39,57	59	3.45	3.70	0.25
14	27,39,57	37	2.60	3.52	0.92
15	27,39,57	64	3.03	3.40	0.37
16	27,39,57	41	3.33	3.73	0.40
17	27,39,57	29	3.40	3.57	0.17
18	13	29	2.24	2.34	0.10
19	59	41	3.32	3.39	0.07
20	55	41	4.20	4.12	-0.08
21	37	29	3.35	3.26	-0.09
22	45,49	51,53	3.20	3.42	0.22
23	45,49	48	3.23	3.10	-0.13
24	45,49	27,39,57	3.76	4.35	0.59
25	34	51,53	4.78	4.46	-0.32
26	32	70	4.47	4.48	0.01

27	70	19	5.00	5.52	0.52
28	26	3,53,71	4.16	4.43	0.27
29	33,102	27,39,57	3.88	4.25	0.37
30	70	24,97,99,109	4.35	3.82	-0.53
31	70	78	3.73	3.53	-0.20
RMSD					0.38

* The large deviation is due to signal overlap

Table S26. Experimental and back-calculated $^{3}J_{HN\text{-}H\alpha}$ coupling constants for the backbone analysis of compound **1**.

**#**	**Residue**	**Exp. (Hz)**	**Calc. average (Hz)**	**Δ (Hz)**
1	Nle-3	8.3	7.9	-0.5
2	Tyr-4	8.7	8.3	-0.4
3	Val-5	8.7	8.3	-0.4
4	Lys-8	5.6	6.2	-0.4
5	Phe-9	7.4	7.8	0.6
6	His-10	8.7	8.2	0.4
7	Val-11	7.9	8.0	0.1
8	Gln-12	7.5	7.9	0.5
RMSD				0.36

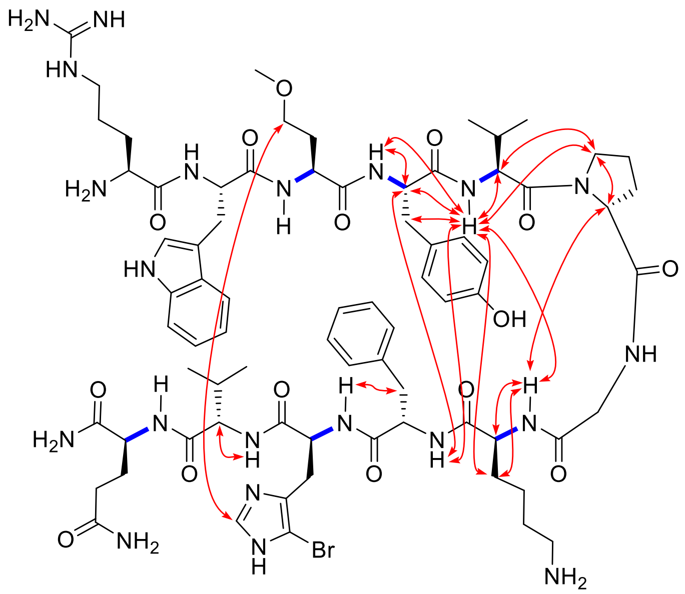

Figure S10. The key NOEs used to derive interproton distances (red) and $^3J_{HN\text{-}H\alpha}$ couplings (blue) used for the conformational analysis of the bakcbone of compound **2**.

Table S27. NOE-derived and back-calculated interproton distances for the backbone analysis of compound **2**.

#	Proton a	Proton b	Exp. (Å)	Calc. average (Å)	Δ (Å)
1	71,3,53	37,64,73	3.67	4.05	0.38
2	51	41	3.96	3.87	-0.09
3	48	59	2.90	2.97	0.07
4	39	41	3.01	2.96	-0.05
5	27,57	59	3.06	2.96	-0.10
6	35	37,64,73	2.20	2.24	0.04
7	51	48	3.13	3.24	0.11
8	33,61,2	41	3.62	3.73	0.11
9	59	41	3.07	3.02	-0.05
10	51	39	4.84	4.59	-0.25
11	37,64,73	41	3.10	3.07	-0.03
12	33,61,2	59	3.61	3.56	-0.05
13	35	41	3.62	3.61	-0.01
14	88	68	2.44	2.41	-0.03
15	51	41	4.14	4.14	0.00
16	34,70,103	41	3.89	3.73	-0.16
17	51	48	2.37	2.41	0.04
18	32	107	4.27	4.14	-0.13
19	51	39	4.66	4.81	0.15
RMSD					0.13

Table S28. Experimental and back-calculated $^{3}J_{HN\text{-}H\alpha}$ coupling constants for the backbone analysis of compound **2**.

#	Residue	Exp. (Hz)	Calc. average (Hz)	Δ (Hz)
1	Hse(Me)-3	8.6	8.6	0.0
2	Tyr-4	9.0	8.9	-0.1
3	Val-5	8.9	9.0	0.1
4	Lys-8	8.7	8.4	-0.2
5	His(Br)-10	7.8	7.8	-0.1
6	Gln-12	7.0	7.2	0.1
RMSD				0.10

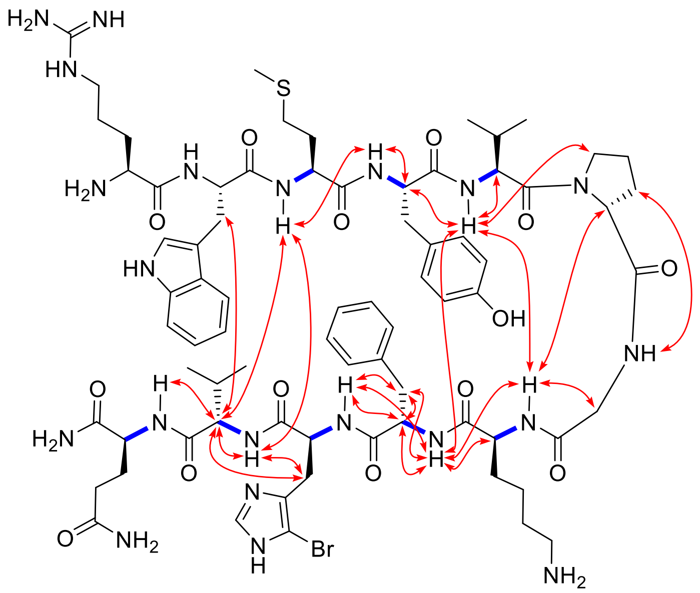

Figure S11. The key NOEs used to derive interproton distances (red) and $^3J_{HN\text{-}H\alpha}$ couplings (blue) used for the conformational analysis of the bakcbone of compound **3**.

Table S29. NOE-derived and back-calculated interproton distances for the backbone analysis of compound **3**.

#	Proton a	Proton b	Exp. (Å)	Calc. average (Å)	Δ (Å)
1	53	59	3.16	3.13	-0.03
2	71	73	3.08	2.92	-0.16
3	71	80	2.48	2.47	-0.01
4	88	64	3.61	3.95	0.34
5	88	68	4.12	3.91	-0.21
6	71	29	3.16	2.97	-0.19
7	26	71	2.49	2.47	-0.02
8	48	59	3.02	2.93	-0.09
9	39	41	2.31	2.33	0.02
10	57	64	3.00	2.96	-0.04
11	62	64	2.31	2.31	0.00
12	62	68	3.71	3.70	-0.01
13	35	37	3.26	3.38	0.12
14	35	41	3.49	3.49	0.00
15	70	71	4.17	4.35	0.18
16	70	73	3.34	3.32	-0.02
17	59	64	3.95	4.04	0.09
18	59	41	2.70	2.66	-0.04
19	37	29	2.91	2.92	0.01
20	64	41	3.32	3.27	-0.05
21	51	41	4.14	4.10	-0.04
22	68	29	4.55	5.23	0.68
23	49	55	3.43	3.37	-0.06
24	88	62	4.37	4.03	-0.34
RMSD					0.19

Table S30. Experimental and back-calculated $^3J_{HN\text{-}H\alpha}$ coupling constants for the backbone analysis of compound **3**.

#	Residue	Exp. (Hz)	Calc. average (Hz)	Δ (Hz)
1	Met-3	8.7	8.5	-0.2
2	Tyr-4	9.1	8.8	-0.3
3	Val-5	8.9	9.1	0.1
4	Lys-8	8.9	8.7	-0.3
5	Phe-9	7.8	8.2	0.4
6	His(Br)-10	8.4	8.1	-0.2
7	Val-11	7.4	7.5	0.1
8	Gln-12	6.9	7.0	0.1
RMSD				0.22

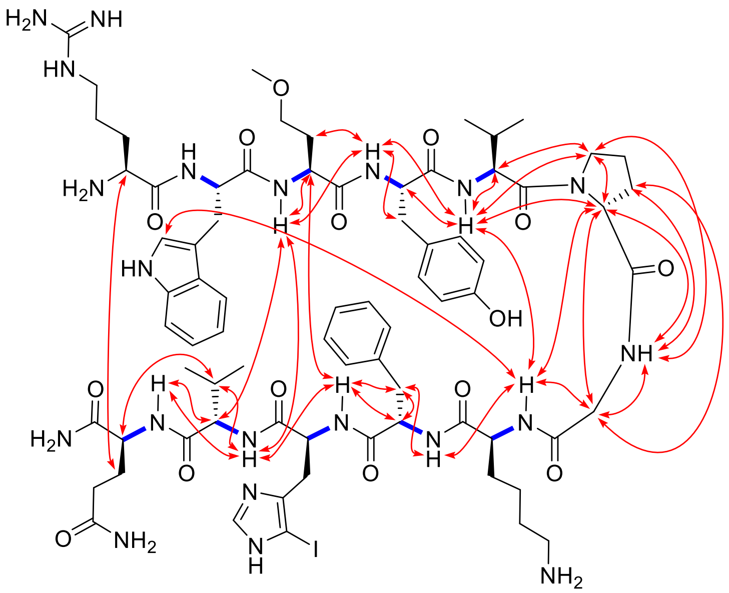

Figure S12. The key NOEs used to derive interproton distances (red) and $^{3}J_{HN\text{-}H\alpha}$ couplings (blue) used for the conformational analysis of the bakcbone of compound **4**.

Table S31. NOE-derived and back-calculated interproton distances for the backbone analysis of compound **4**.

#	Proton a	Proton b	Exp. (Å)	Calc. average (Å)	Δ (Å)
1	53	59	3.03	3.06	0.03
2	53	55	2.52	2.61	0.09
3	88	68	4.38	4.67	0.29
4	53	48	3.08	3.06	-0.02
5	53	59	2.59	2.61	0.02
6	53	55	2.58	2.61	0.03
7	71	80	3.94	4.14	0.20
8	71	29	4.14	3.92	-0.22
9	24	59	2.66	2.60	-0.06
10	48	59	5.32	5.12	-0.20
11	48	55	3.12	2.97	-0.15
12	88	62	4.35	4.40	0.05
13	88	64,73,37	3.50	3.32	-0.18
14	88	68	2.35	2.37	0.02
15	48	41	3.24	3.23	-0.01
16	39	41	2.27	2.30	0.03
17	27,57	68	3.67	3.60	-0.07
18	27,57	29	3.32	3.30	-0.02
19	62	68	3.81	4.04	0.23
20	35	64,73,37	3.92	3.87	-0.05
21	35	41	3.64	3.86	0.22
22	51	55	3.48	3.43	-0.05
23	59	64,73,37	3.85	4.39	0.54
24	59	41	4.67	4.72	0.05
25	64,73,37	68	4.18	4.22	0.04
26	51	39	5.23	5.27	0.04

27	51	41	5.89	5.16	-0.73
28	64,73,37	41	3.88	3.89	0.01
29	64,73,37	29	2.60	2.61	0.01
30	68	29	3.18	3.16	-0.02
31	51	48	5.78	5.17	-0.61
32	33	64,73,37	2.65	2.61	-0.04
33	77	78	3.01	3.01	0.00
34	77	64,73,37	3.21	3.16	-0.05
35	86	3	3.87	3.62	-0.25
36	49	53	3.87	3.72	-0.15
37	49	55	2.43	2.38	-0.05
38	88	62	4.19	3.96	-0.23
RMSD					0.22

Table S32. Experimental and back-calculated $^{3}J_{HN\text{-}H\alpha}$ coupling constants for the backbone analysis of compound **4**.

**#**	**Residue**	**Exp. (Hz)**	**Calc. average (Hz)**	**Δ (Hz)**
1	Trp-2	8.4	8.1	-0.3
2	Hse(Me)-3	8.9	8.8	-0.2
3	Tyr-4	9.2	8.8	-0.5
4	Val-5	8.8	8.9	0.1
5	Lys-8	8.9	8.8	-0.2
6	Phe-9	7.1	7.5	0.4
7	His(I)-10	8.2	8.2	0.0
8	Val-11	7.7	7.8	0.1
9	Gln-12	7.0	7.0	0.0
RMSD				0.25

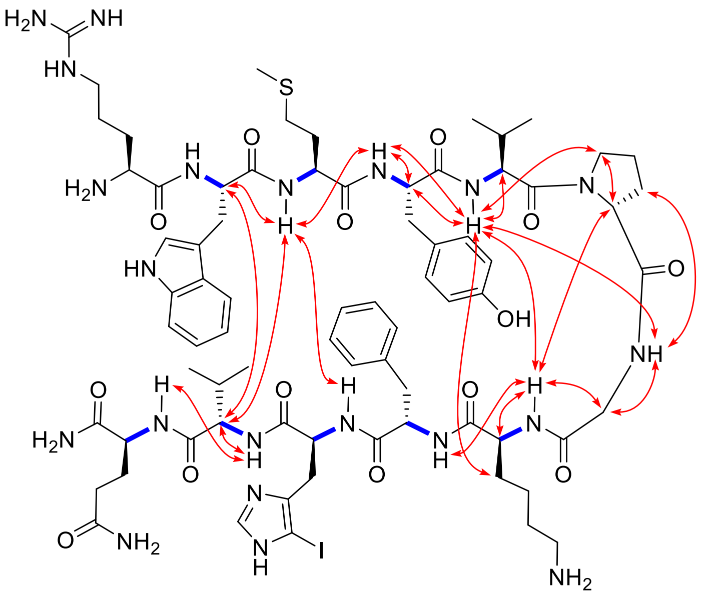

Figure S13. The key NOEs used to derive interproton distances (red) and $^{3}J_{HN\text{-}H\alpha}$ couplings (blue) used for the conformational analysis of the bakcbone of compound **5**.

Table S33. NOE-derived and back-calculated interproton distances for the backbone analysis of compound **5**.

#	Proton a	Proton b	Exp. (Å)	Calc. average (Å)	Δ (Å)
1	53	59	3.15	3.15	0.00
2	53	55	2.62	2.66	0.04
3	53	59	3.19	3.15	-0.04
4	53	55	2.73	2.66	-0.07
5	71	13	3.29	3.26	-0.03
6	71	73	3.11	2.93	-0.18
7	71	29	3.86	4.20	0.34
8	48	59	4.07	3.98	-0.09
9	39	41	2.98	2.97	-0.01
10	57	59	2.90	2.89	-0.01
11	13	29	2.34	2.35	0.01
12	35	37	3.00	2.94	-0.06
13	35	41	2.27	2.28	0.01
14	59	64,80	3.66	3.67	0.01
15	59	41	3.22	3.18	-0.04
16	51	48	4.05	3.96	-0.09
17	55	41	4.04	4.03	-0.01
18	73	68	3.80	3.74	-0.06
19	37	41	3.52	3.51	-0.01
20	37	29	3.14	3.14	0.00
21	68	29	4.49	4.49	0.00
22	61	41	3.76	3.72	-0.04
23	49	55	4.21	4.20	-0.01
24	51	41	3.76	3.84	0.08
25	51	41	4.17	4.20	0.03
RMSD					0.09

Table S34. Experimental and back-calculated $^3J_{HN\text{-}H\alpha}$ coupling constants for the backbone analysis of compound **5**.

#	Residue	Exp. (Hz)	Calc. average (Hz)	Δ (Hz)
1	Trp-2	7.8	7.7	-0.1
2	Met-3	8.5	8.3	-0.2
3	Tyr-4	8.3	8.3	0.0
4	Val-5	8.9	9.0	0.0
5	Lys-8	7.8	7.9	0.1
6	Phe-9	7.4	7.5	0.2
7	His(I)-10	8.2	8.1	-0.2
8	Val-11	8.1	8.2	0.2
9	Gln-12	8.3	8.1	-0.2
RMSD				0.13

Table S35. Populations of backbone conformations selected by StereoFitter for compounds **1-5**.

Conf. #	1	2	3	4	5
1	18%	27%	14%	15%	15%
2	10%	19%	13%	14%	13%
3	9%	17%	13%	14%	9%
4	9%	14%	12%	8%	7%
5	8%	6%	11%	7%	6%
6	6%	5%	10%	6%	5%
7	6%		6%	5%	5%
8	6%		3%	4%	5%
9	5%		3%	4%	5%
10	4%		3%	3%	5%
11	4%				4%
12					4%
13					4%
14					4%
15					4%
β-hairpin	22%	44%	45%	40%	65%

Conf. 1: 18%

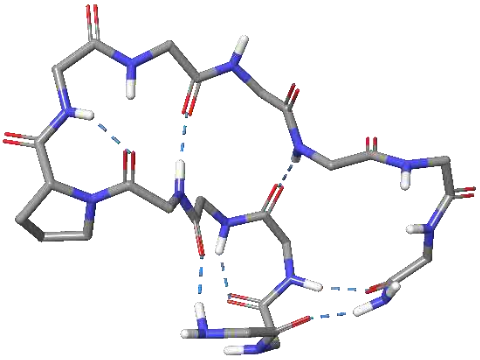

Conf. 2: 10%

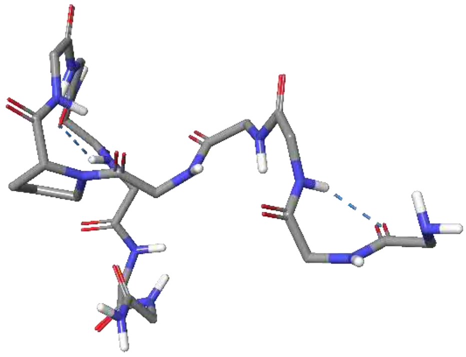

Conf. 3: 9%

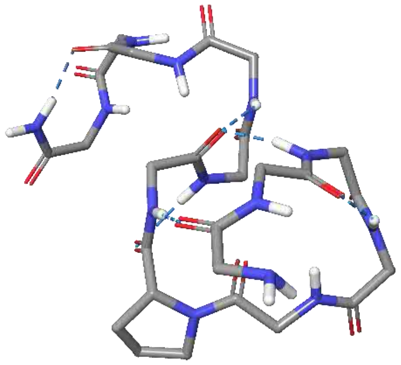

Conf. 4: 9%

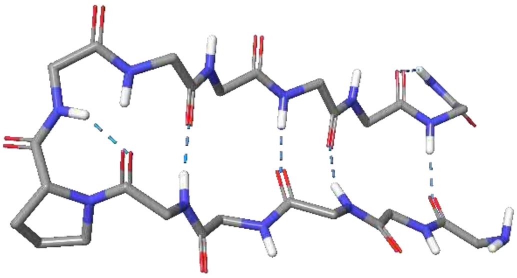

Conf. 5: 8%

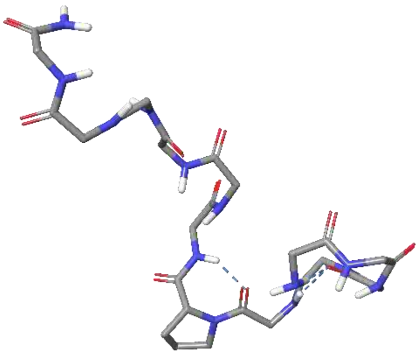

Conf.6: 6%

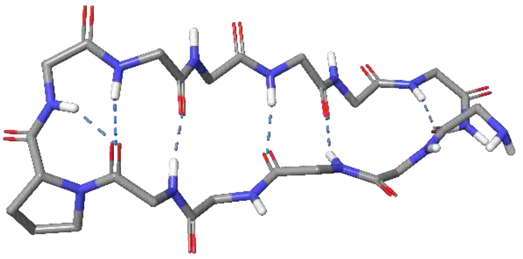

Conf. 7: 6%

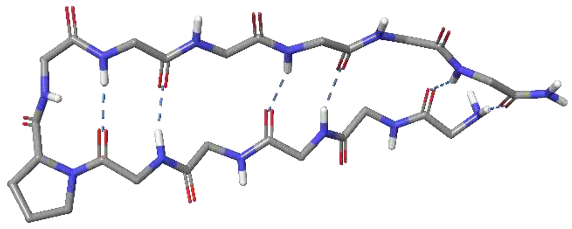

Conf. 8: 6%

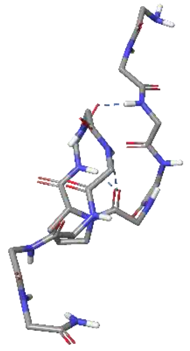

Conf. 9: 5%

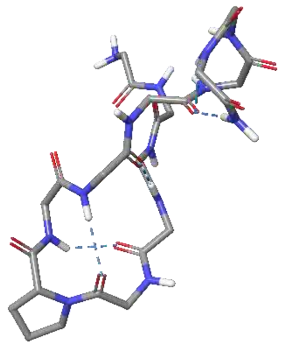

Conf. 10: 4%

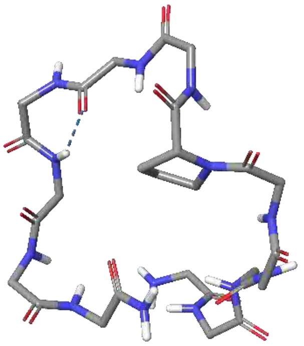

Conf. 11: 4%

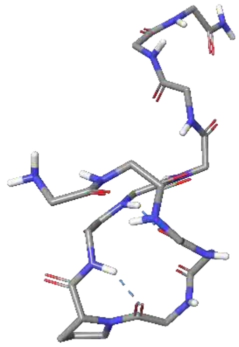

Figure S14. The backbone conformations for compound **1** with populations > 3% selected by StereoFitter. Side chains and non-polar hydrogens are omitted for clarity. Hydrogen bonds are visualized as blue dotted lines.

Conf. 1: 27%

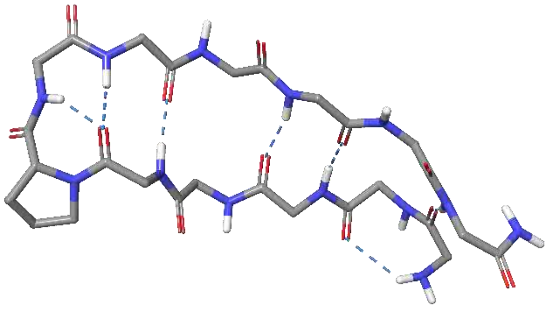

Conf. 2: 19%

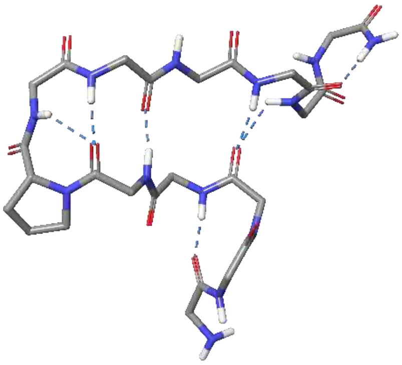

Conf. 3: 17%

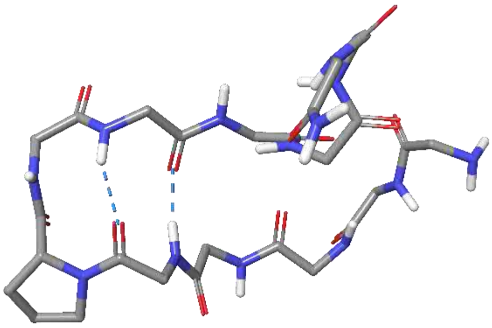

Conf. 4: 14%

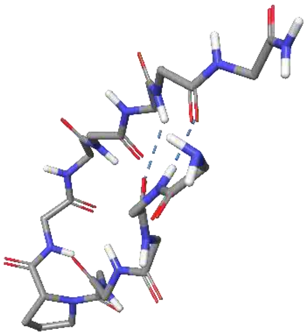

Conf. 5: 6%

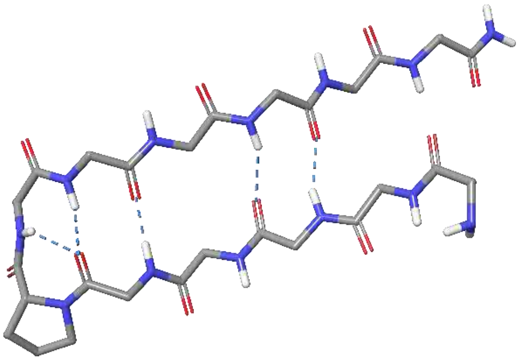

Conf.6: 5%

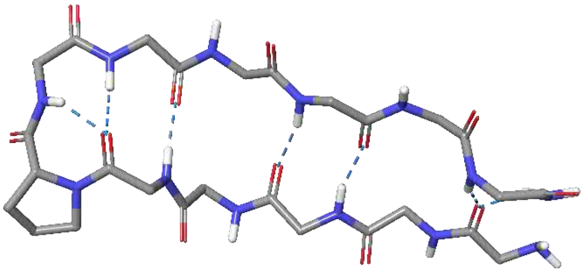

Figure S15. The backbone conformations for compound **2** with populations > 3% selected by StereoFitter. Side chains and non-polar hydrogens are omitted for clarity. Hydrogen bonds are visualized as blue dotted lines.

Conf. 1: 14%

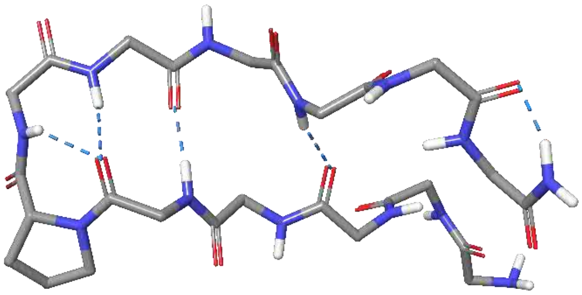

Conf. 2: 13%

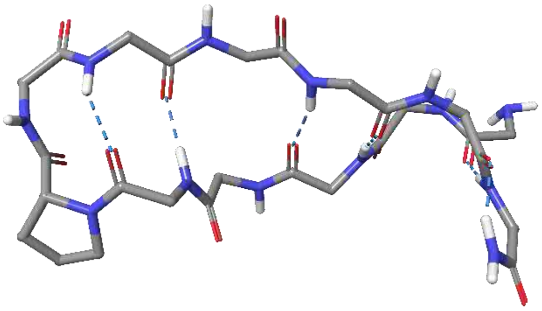

Conf. 3: 13%

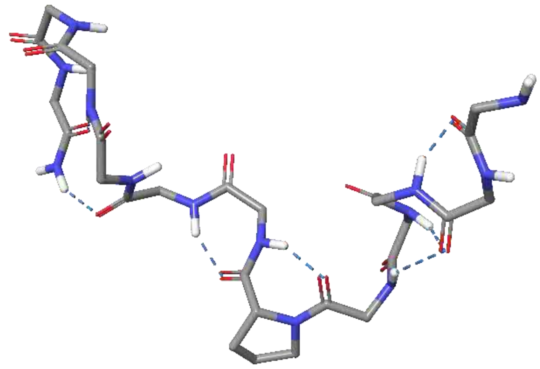

Conf. 4: 12%

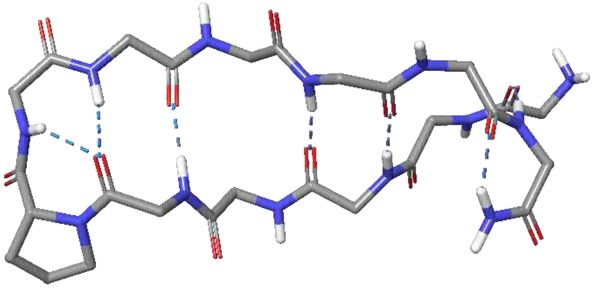

Conf. 5: 11%

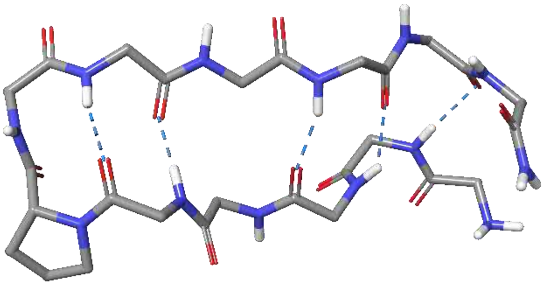

Conf.6: 10%

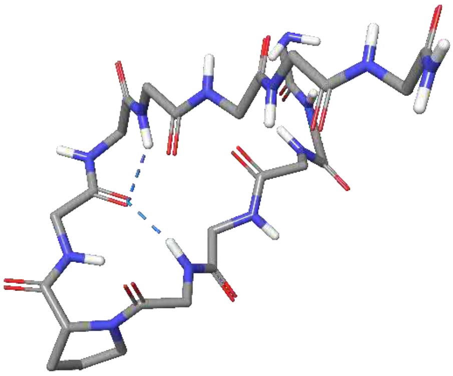

Conf. 7: 6%

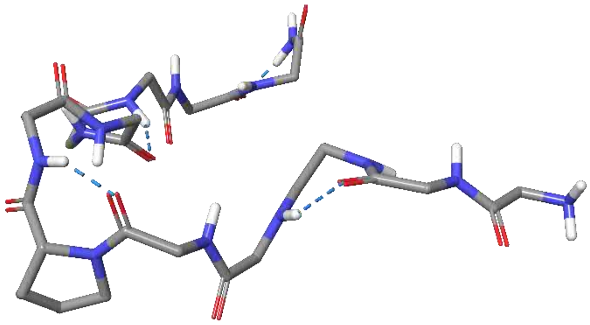

Conf. 8: 3%

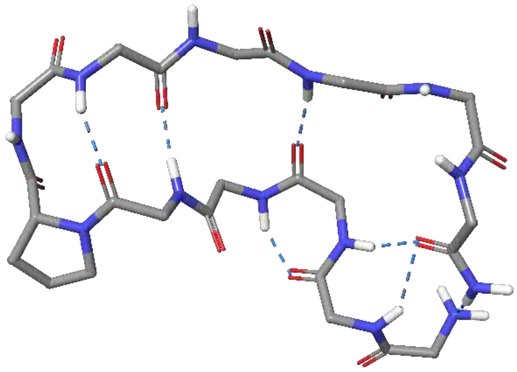

Conf. 9: 3%

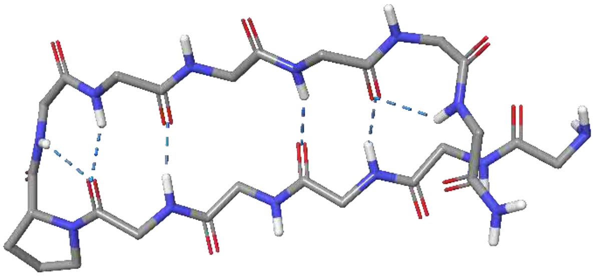

Conf. 10: 3%

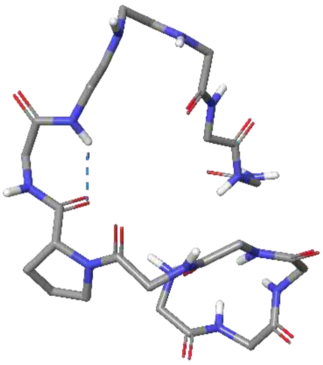

Figure S16. The backbone conformations for compound **3** with populations > 3% selected by StereoFitter. Side chains and non-polar hydrogens are omitted for clarity. Hydrogen bonds are visualized as blue dotted lines.

Conf. 1: 15%

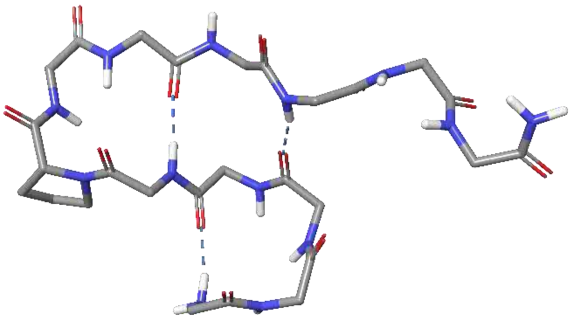

Conf. 2: 14%

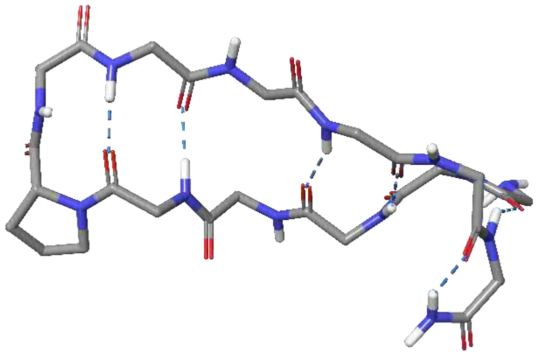

Conf. 3: 14%

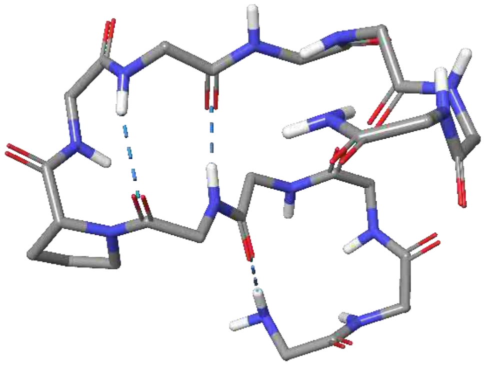

Conf. 4: 8%

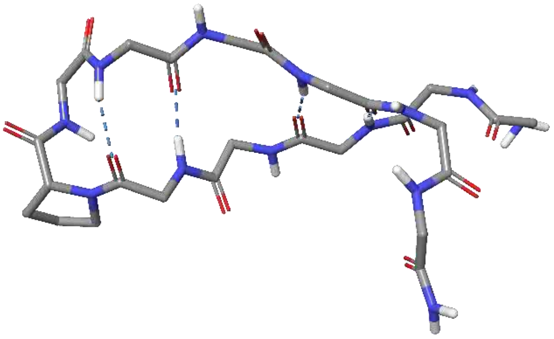

Conf. 5: 7%

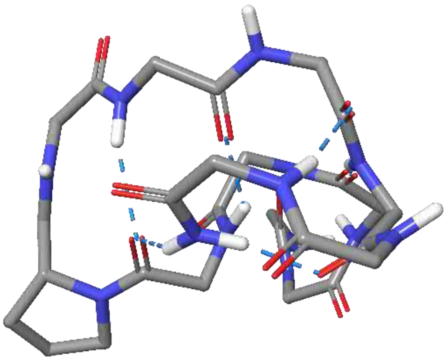

Conf.6: 6%

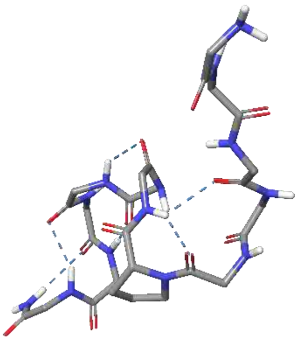

Conf. 7: 5%

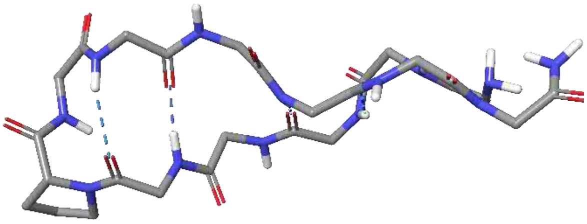

Conf. 8: 4%

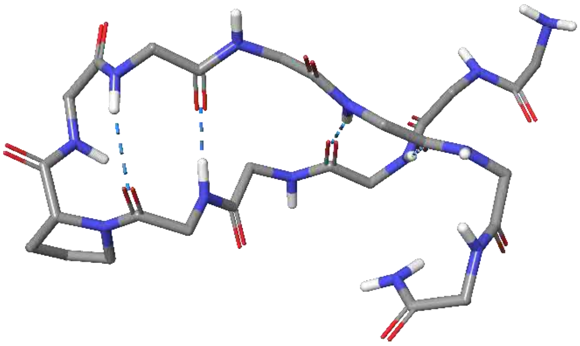

Conf. 9: 4%

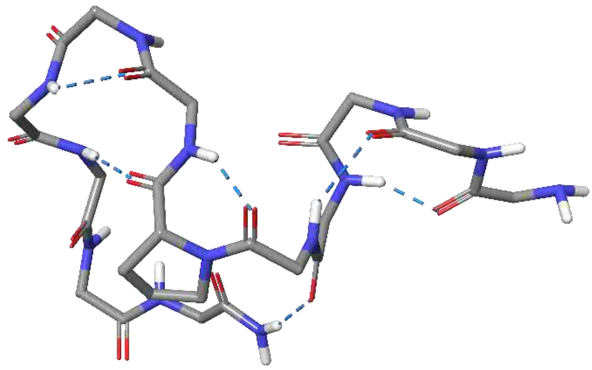

Conf. 10: 3%

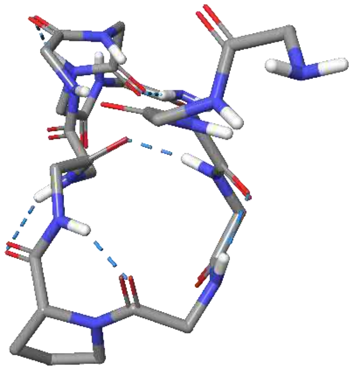

Figure S17. The backbone conformations for compound **4** with populations > 3% selected by StereoFitter. Side chains and non-polar hydrogens are omitted for clarity. Hydrogen bonds are visualized as blue dotted lines.

Conf. 1: 15%

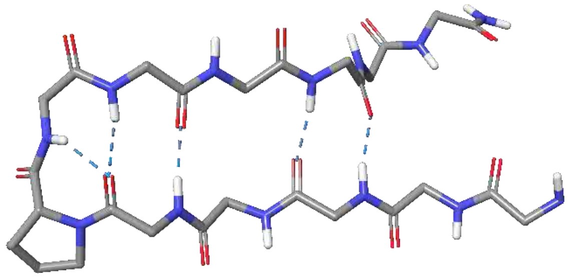

Conf. 2: 13%

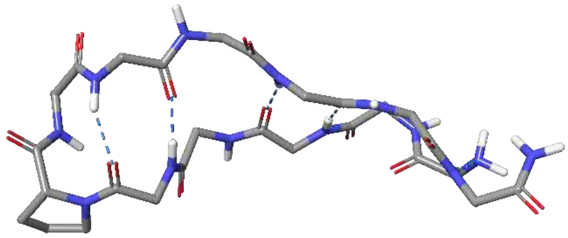

Conf. 3: 9%

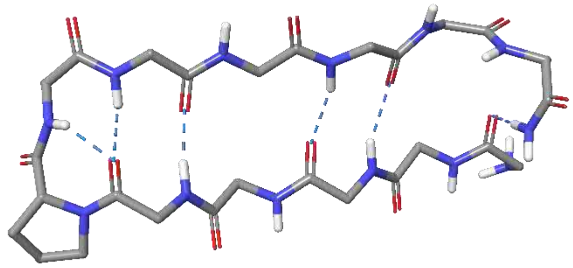

Conf. 4: 7%

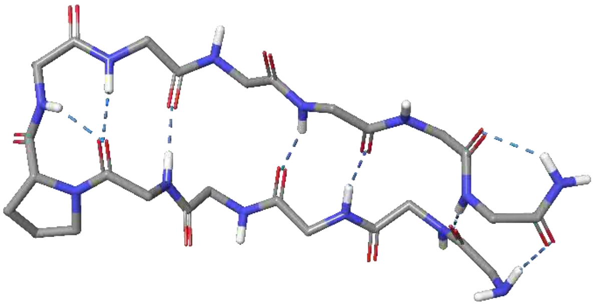

Conf. 5: 6%

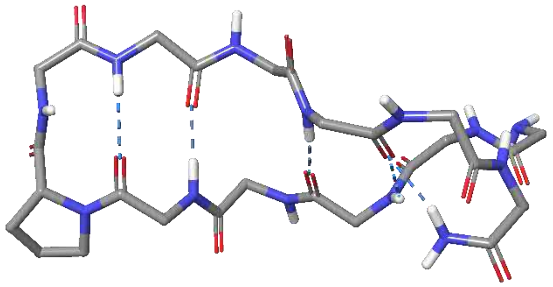

Conf.6: 5%

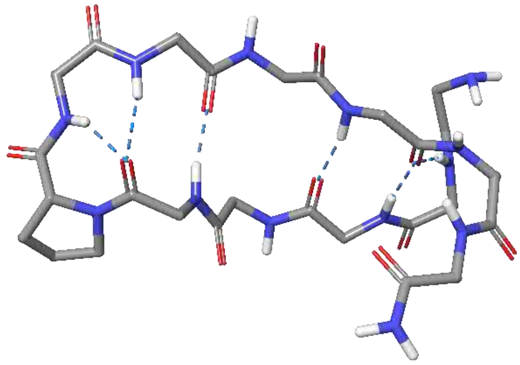

Conf. 7: 5%

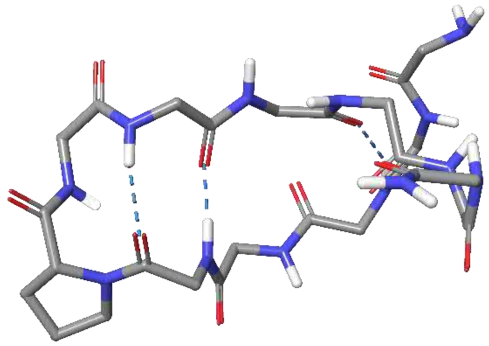

Conf. 8: 5%

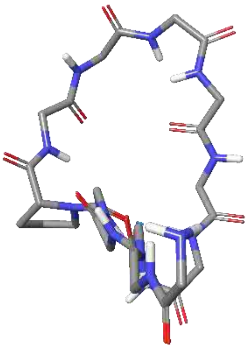

Conf. 9: 5%

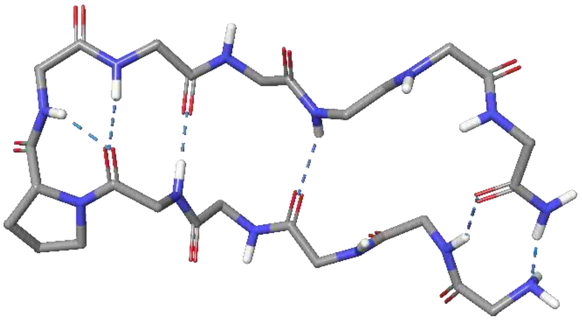

Conf. 10: 5%

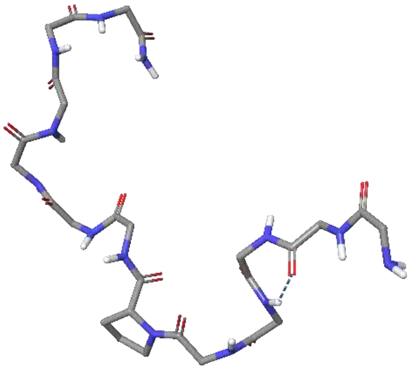

Conf. 11: 4%

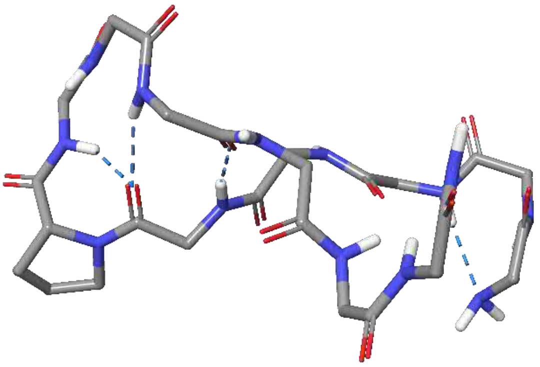

Conf. 12: 4%

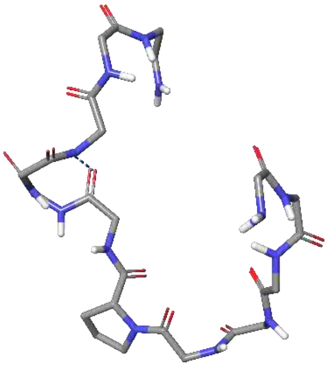

Conf. 13: 4%

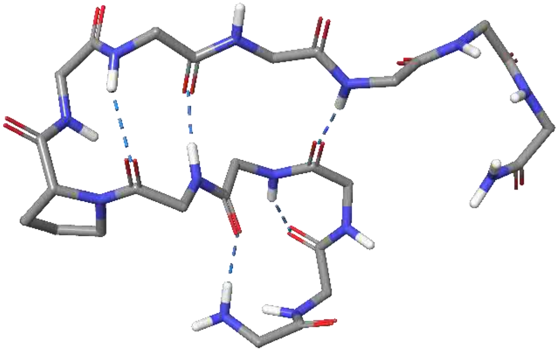

Conf. 14: 4%

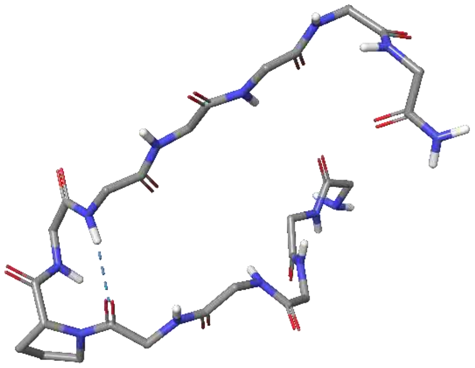

Conf. 15: 4%

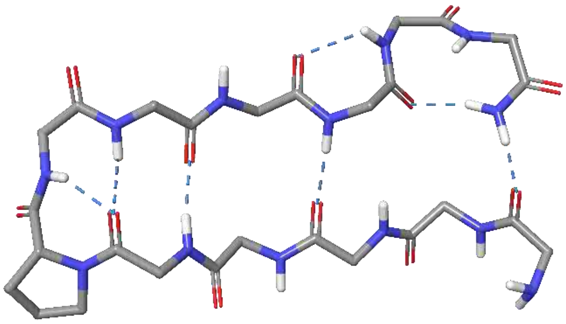

Figure S18. The backbone conformations for compound **5** with populations > 3% selected by StereoFitter. Side chains and non-polar hydrogens are omitted for clarity. Hydrogen bonds are visualized as blue dotted lines.

5 Side chain analysis using RDCs and NOEs

The side chain geometry of compounds **2**-**4** capable of halogen bonding was refined in a second fitting step. The RDCs and NOE-derived interproton distances highlighted in Figures S19-S22 and listed in Tables S36-S43 were deconvoluted against the side chain-sampled theoretical ensembles using the StereoFitter plugin (v. 1.1.6) for MestreNova (v. 15.0.1).

The alignment tensor was computed by singular value decomposition (SVD)[14] using the single tensor approximation. Hereby, all conformers were superimposed by minimizing the deviation of backbone heavy atom coordinates. Determination of the alignment tensor allowed the back-calculation of theoretical RDC values which were used in combination with NOE-derived distances to find the best fitting populations by minimizing χ^2, as shown before. The conformer populations obtained from StereoFitter were fed to Mspin (v. 2.6.1)[15] to refine the alignment tensor. The SVD condition number (CN) describes how sensitive the calculated alignment tensor is to variations in the RDC input data, or with other words whether there are enough independent bond vectors for the description of the alignment tensor. A value closer to 0 indicates a better defined system, with condition numbers below 50 indicating a system well enough described by RDC for getting reliable results. The general degree of order (GDO) is an estimate of the degree of alignment determined from the size of the RDC values and the tensorial information. The GDO values agree well with the type of alignment medium used. The quality of fit between experimental and back-calculated RDCs from the alignment tensor is described by the Cornilescu quality factor *Q*, which is given by:[16]

$$Q = \sqrt{\frac{\sum_i \left(D_i^{\exp} - D_i^{\mathrm{calc}}\right)^2}{\sum_i \left(D_i^{\exp}\right)^2}}.$$

Q is directly correlated to the Pearson coefficient R_P. The quality of fit was deemed good with $Q < 0.42$, corresponding to $R_P > 0.9$. Quality measures for the RDC fitting are listed in Table S36.

Table S36. Quality measures for the fitting of RDC data by MSpin. Robustness of the alignment tensor to variations in the experimental data is described by the SVD CN. The degree of alignment is indicated by the GDO. The correlation between experimental and back-calculated RDC data is evaluated by *Q* factor, Pearson correlation coefficient R_P and RMSD.

Compound	SVD CN	GDO	*Q*	R_P	RMSD (Hz)
2	16.7	0.2%	0.322	0.947	0.565
3	12.7	0.2%	0.005	1.000	0.016
4	9.2	0.2%	0.219	0.972	0.912
5	5.5	0.2%	0.376	0.927	1.620

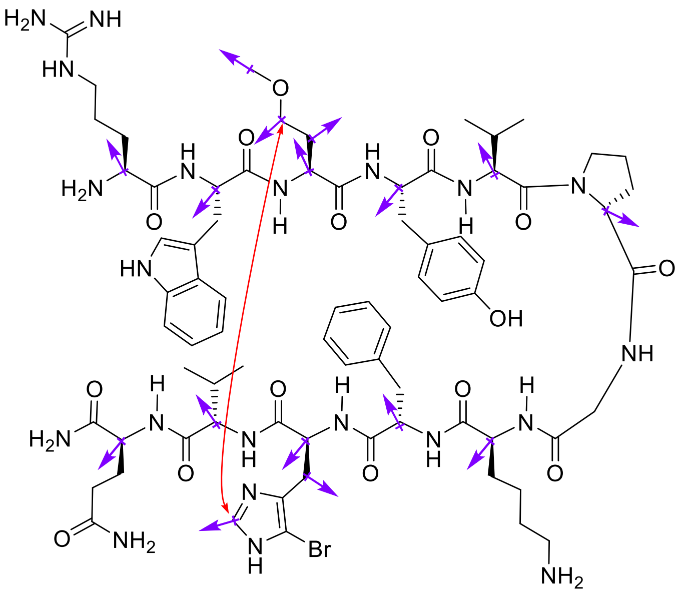

Figure S19. Structure of compound **2** with C-H RDCs (purple) and NOE-derived interproton distances (red) used for the analysis of the side chain orientation of the amino acids involved into the halogen bonding. RDCs from C-H_n groups (n = 2, 3) were averaged as discussed above.

Table S37. Experimental and back-calculated RDC values for the side chain analysis of compound **2**.

#	Residue	Assignment	Exp. (Hz)	Calc. average (Hz)	Δ (Hz)
1	Arg-1	3	0.5	0.5	0.0
2	Trp-2	13	1.5	1.1	-0.3
3	Hse(Me)-3	27	0.8	-0.1	-0.9
4		33	-0.6	0.2	0.7
5		32	0.5	0.4	0.0
6		110	-5.9	-5.8	0.0
7	Tyr-4	35	-1.2	-0.6	0.6
8	Val-5	39	2.2	2.4	0.2
9	D-Pro-6	48	0.2	0.4	0.1
10	Lys-8	57	0.3	0.5	0.2
11	Phe-9	62	-0.1	-0.1	0.0
12	His(Br)-10	70	1.0	0.2	-0.8
13		107	0.3	-1.1	-1.3
14	Val-11	71	0.9	0.9	0.0
15	Gln-12	78	0.1	1.1	1.0
RMSD					0.58

Table S38. NOE-derived and back-calculated interproton distances for the side chain analysis of compound **2**.

#	Proton a	Proton b	Exp. (Å)	Calc. average (Å)	Δ (Å)
1	32	107	4.66	4.75	0.09
RMSD					0.09

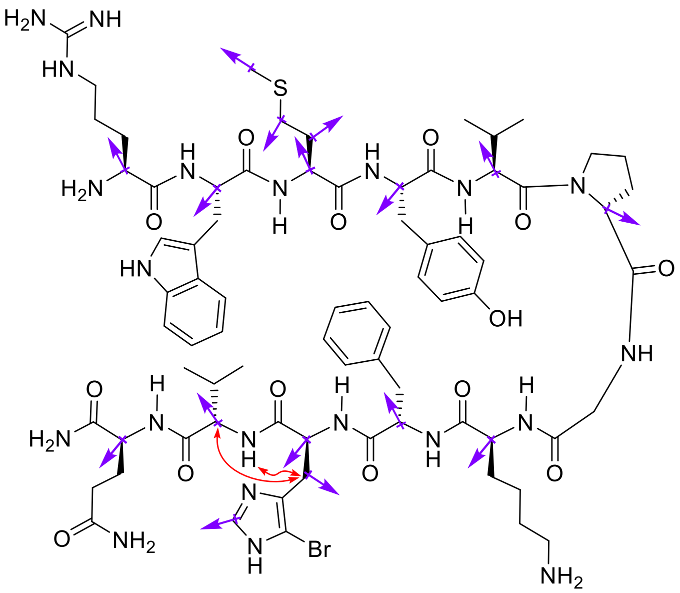

Figure S20. Structure of compound **3** with C-H RDCs (purple) and NOE-derived interproton distances (red) used for the side chain analysis. RDCs from C-H_n groups (n = 2, 3) were averaged, as described above.

Table S39. Experimental and back-calculated RDC values for the side chain analysis of compound **3**.

#	Residue	Assignment	Exp. (Hz)	Calc. average (Hz)	Δ (Hz)
1	Arg-1	3	-0.4	-0.4	0.0
2	Trp-2	13	-0.6	-0.6	0.0
3	Met-3	27	0.1	0.1	0.0
4		33	3.3	3.3	0.0
5		32	-0.1	-0.1	0.0
6		110	-13.7	-13.7	0.0
7	Tyr-4	35	0.8	0.8	0.0
8	Val-5	39	0.8	0.8	0.0
9	D-Pro-6	48	1.3	1.3	0.0
10	Lys-8	57	-1.0	-1.1	0.0
11	Phe-9	62	-0.3	-0.3	0.0
12	His(Br)-10	66	1.0	1.0	0.0
13		70	-0.1	-0.1	0.0
14		107	-2.9	-2.9	0.0
15	Val-11	71	0.8	0.8	0.0
16	Gln-12	78	1.0	0.9	0.0
RMSD					0.02

Table S40. NOE-derived and back-calculated interproton distances for the side chain analysis of compound **3**.

#	Proton a	Proton b	Exp. (Å)	Calc. average (Å)	Δ (Å)
1	70	71	4.55	5.17	0.62
2	70	73	3.43	3.35	-0.08
RMSD					0.44

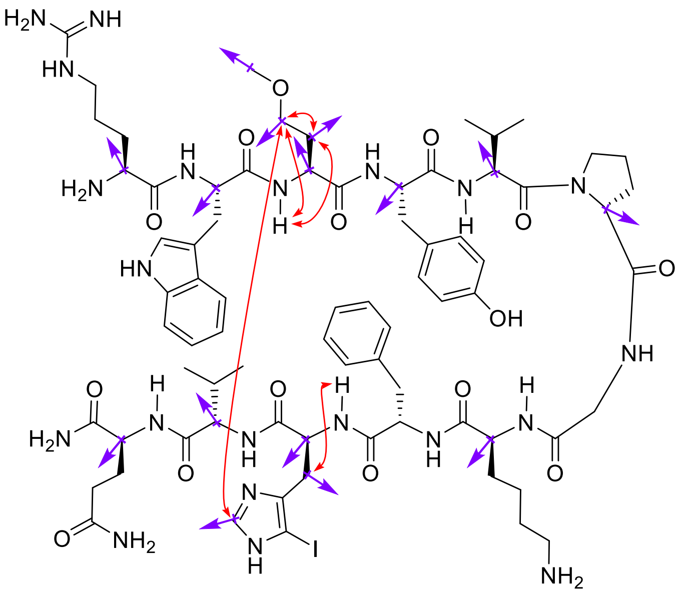

Figure S21. Structure of compound **4** with C-H RDCs (purple) and NOE-derived interproton distances (red) used for the side chain analysis. RDCs from C-H_n groups (n = 2, 3) were averaged.

Table S41. Experimental and back-calculated RDC values for the side chain analysis of compound **4**.

#	Residue	Assignment	Exp. (Hz)	Calc. average (Hz)	Δ (Hz)
1	Arg-1	3	2.5	1.8	-0.6
2	Trp-2	13	0.4	0.1	-0.3
3	Hse(Me)-3	27	1.0	0.8	-0.2
4		33	-1.8	1.3	3.1
5		32	-2.6	-1.7	0.9
6		110	-7.4	-7.3	0.0
7	Tyr-4	35	-2.2	-3.1	-1.0
8	Val-5	39	-2.7	-1.0	1.7
9	D-Pro-6	48	-0.1	0.3	0.4
10	Lys-8	57	1.4	1.1	-0.3
11	His(I)-10	66	-1.7	-2.2	-0.4
12		70	-1.8	1.3	3.1
13		107	-12.9	-14.6	-1.7
14	Val-11	71	1.1	0.1	-1.1
15	Gln-12	78	0.3	0.2	-0.1
RMSD					1.37

Table S42. NOE-derived and back-calculated interproton distances for the side chain analysis of compound **4**.

#	Proton a	Proton b	Exp. (Å)	Calc. average (Å)	Δ (Å)
1	33	32	3.24	2.63	-0.61
2	33	29	3.28	2.84	-0.44
3	70,26	68	3.26	3.05	-0.21
4	32	107	4.40	4.82	0.42
5	32	29	3.81	3.58	-0.23
RMSD					1.13

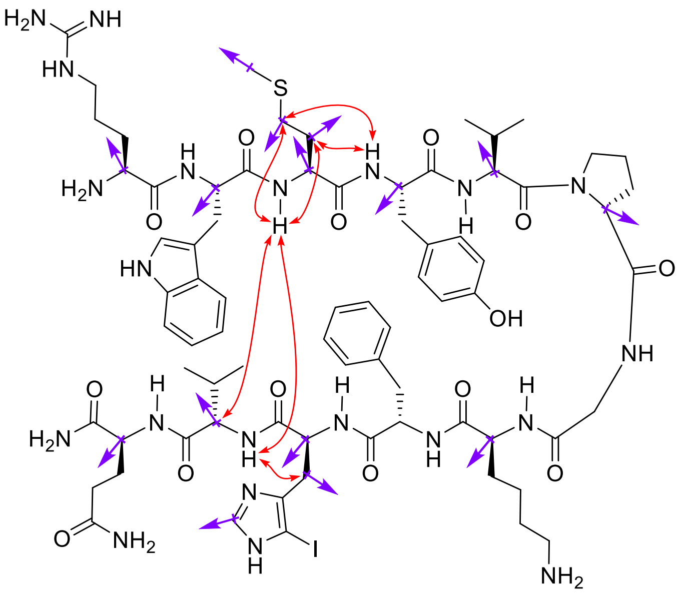

Figure S22. Structure of compound **5** with C-H RDCs (purple) and NOE-derived interproton distances (red) used for the side chain analysis. RDCs from C-H_n groups (n = 2, 3) were averaged, as described above.

Table S43. Experimental and back-calculated RDC values for the side chain analysis of compound **5**.

#	Residue	Assignment	Exp. (Hz)	Calc. average (Hz)	Δ (Hz)
1	Arg-1	3	-0.9	-1.0	0.0
2	Trp-2	13	-2.4	-1.7	0.7
3	Hse(Me)-3	27	-2.4	-2.7	-0.3
4		33	2.5	-1.9	-4.3
5		32	0.6	0.7	0.1
6		110	-12.9	-12.8	0.0
7	Tyr-4	35	-1.2	-2.6	-1.4
8	Val-5	39	-3.6	-3.1	0.5
9	D-Pro-6	48	1.2	0.8	-0.4
10	Lys-8	57	2.5	-0.3	-2.8
11	His(I)-10	66	3.6	4.2	0.6
12		70	1.0	4.6	3.6
13		107	5.7	5.8	0.2
14	Val-11	71	2.9	3.8	0.9
15	Gln-12	78	4.2	4.2	0.0
RMSD					1.71

Table S44. NOE-derived and back-calculated interproton distances for the side chain analysis of compound **5**.

#	Proton a	Proton b	Exp. (Å)	Calc. average (Å)	Δ (Å)
1	71	29	3.86	4.49	0.63
2	68	29	3.14	3.09	-0.05
3	33,61,77	29	3.37	3.55	0.18
4	2,33	37	3.44	3.30	-0.14
5	2,33	29	3.50	3.40	-0.10
6	32,49,85	37	4.68	4.30	-0.38
7	32,49,85	29	3.82	3.63	-0.19
8	24,70	68	3.58	3.35	-0.23
9	26,70	68	3.30	3.34	0.04
RMSD					0.60

6 DFT calculations

A total of five model dimers were constructed to refine the geometries of residues potentially involved in halogen bonding. The geometry of the His(I) ···Met dimer was extracted from the backbone ensemble of compound **5**, capped with an acetyl group (ACE) at the N-terminus and an N-methyl amide group (NHE) at the C-terminus. The geometries of the other three halogen-containing dimers were obtained by substituting specific atoms in the His(I)···Met dimer, including His(I)···Hse(Me), His(Br)···Met and His(Br)···Hse(Me). The reference model dimer, His···Nle, was constructed based on the optimized geometry of the His(I)···Met dimer, whose side chain position was reoptimized while the rest of the system remained frozen. Geometry optimizations of model systems were carried out at the M062X/6-311+g(d,p) level of theory[17] (SDD basis set for iodine and bromine).[18] Solvent effects were simulated by the Polarizable Continuum Model (PCM) with water as solvent.[19] Single-point calculation was performed based on the optimized structures at the M062X/6-311+g(d,p) level of theory[14] to get the interaction energy. The basis set superposition error (BSSE) was evaluated by the counterpoise method of Boys and Bernardi.[20] All calculations were carried out with the Gaussian 16 program package.[21] The interaction energy ΔE was defined as the difference between the energy of the complex and the sum of the energies of the monomers in the geometry within the complex. The interaction energy between the two residues was then assessed by the following equation:

$$\Delta E = E_{\mathrm{com}} - E_{\mathrm{A}} - E_{\mathrm{B}} + \mathrm{BSSE}$$

where ΔE is the interaction energy, E_{com} is the energy of the whole complex, E_A and E_B are the energies of single residues and BSSE the basis set superposition error. Distance criteria used to define the presence of a halogen bond are given in Table S45. Refined geometries and electrostatic potential (ESP) maps showing the σ-hole are depicted in Figures S23 and S24.

Table S45. Distance criterion of XB length

**XB type**	Br-O	Br-S	I-O	I-S
**XB length**	≤3.37 Å	≤3.65 Å	≤3.50 Å	≤3.78 Å

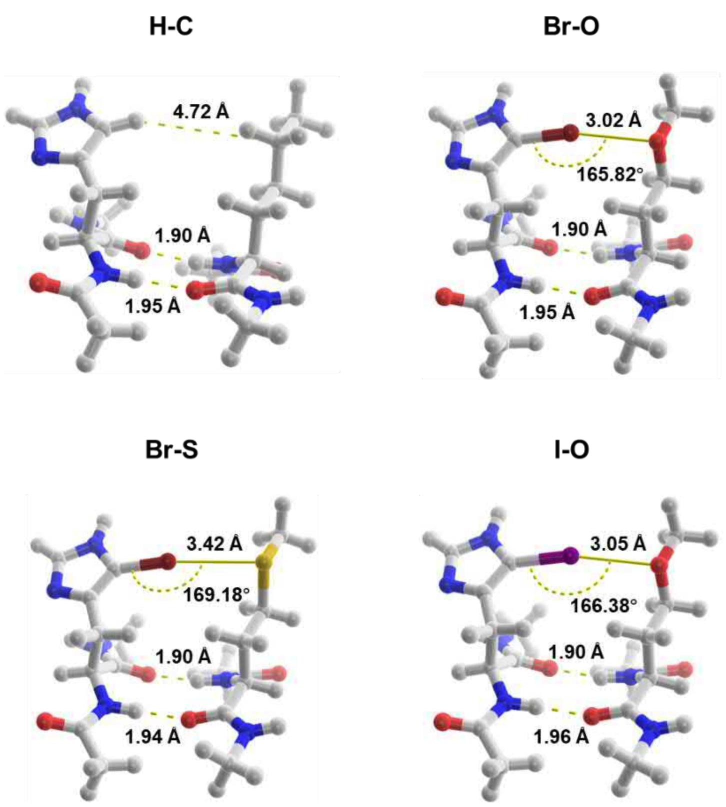

Figure S23. The equilibrium geometries for His···Nle, His(Br) ···Hse(Me), His(Br)···Met and His(I) ···Hse(Me) dimers as found in compounds **1-4**.

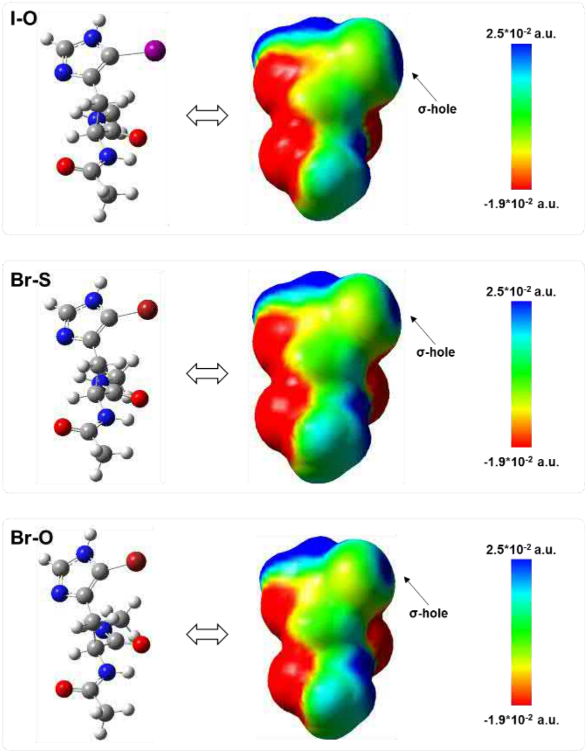

Figure S24. ESP maps of halogenated histidine (isoval = 0.0004).

7 NMR spectra

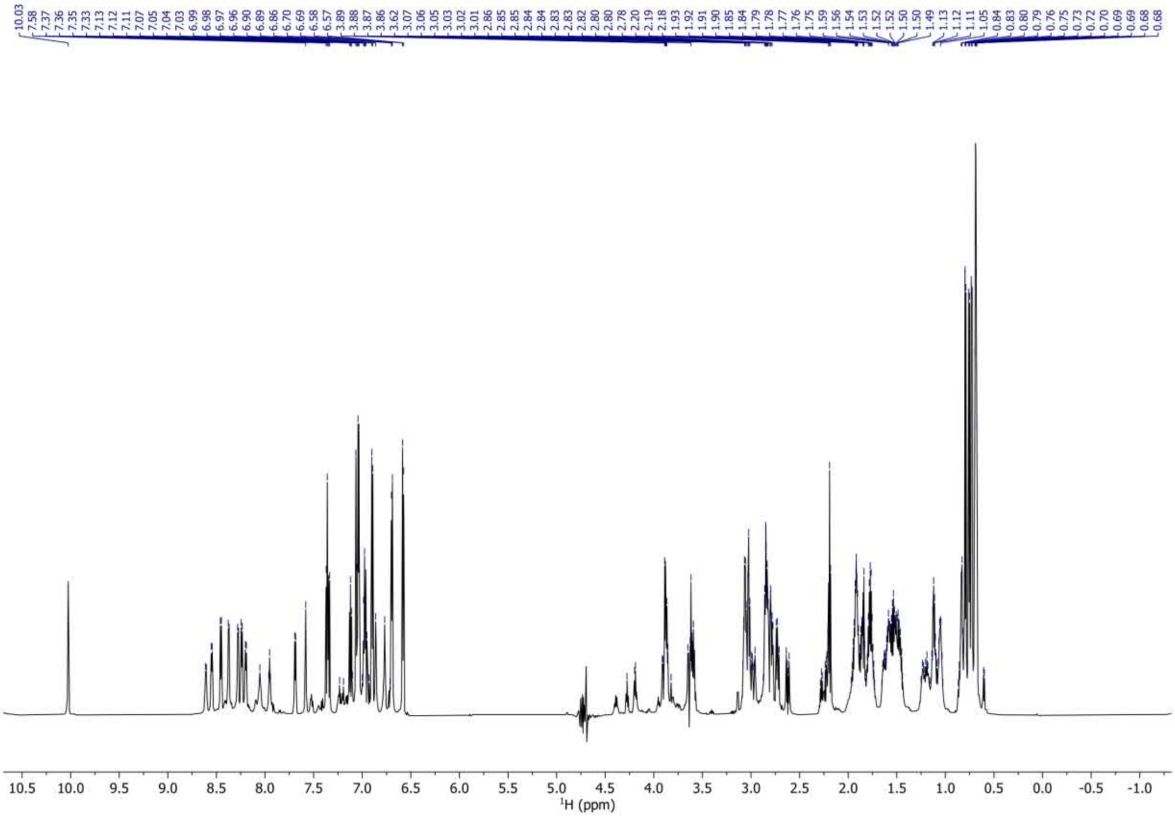

Figure S25. ^{1}H NMR spectrum of compound **1** (800 MHz, H_2O:D_2O 9:1, 25 °C).

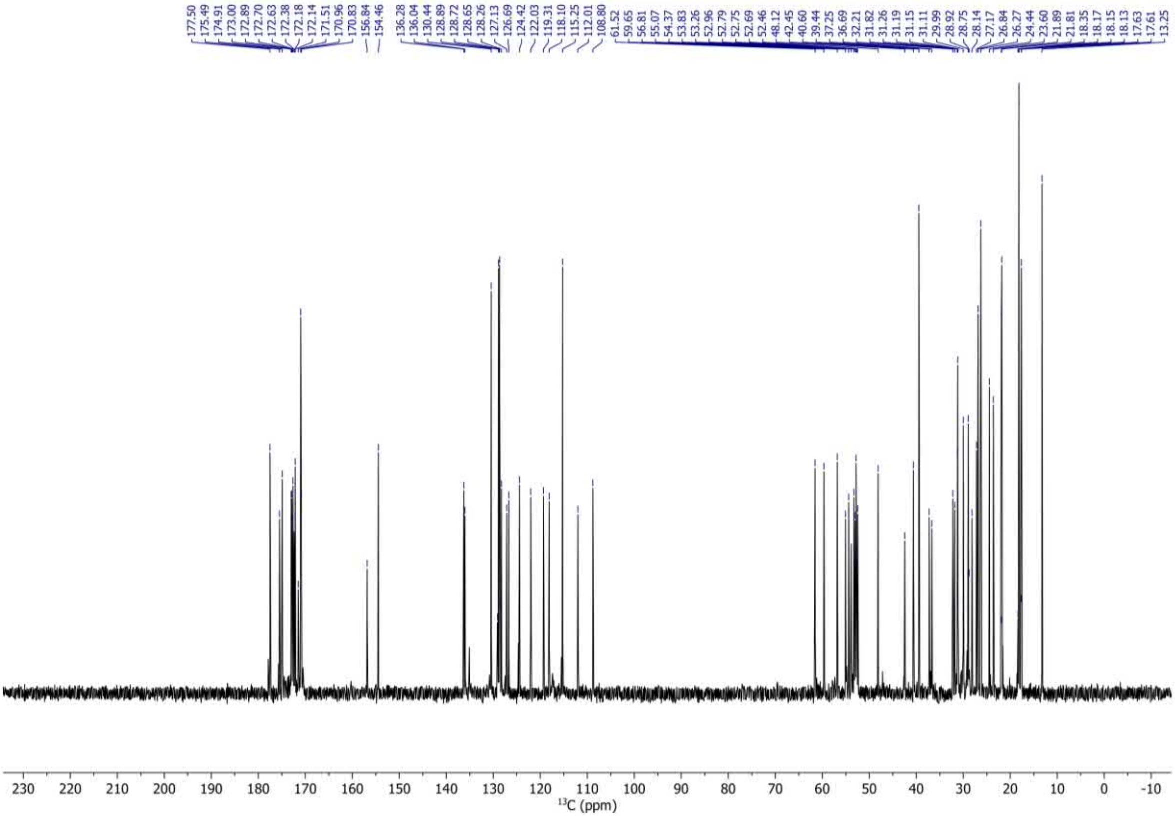

Figure S26. ^{13}C NMR spectrum of compound **1** (201 MHz, H_2O:D_2O 9:1, 25 °C).

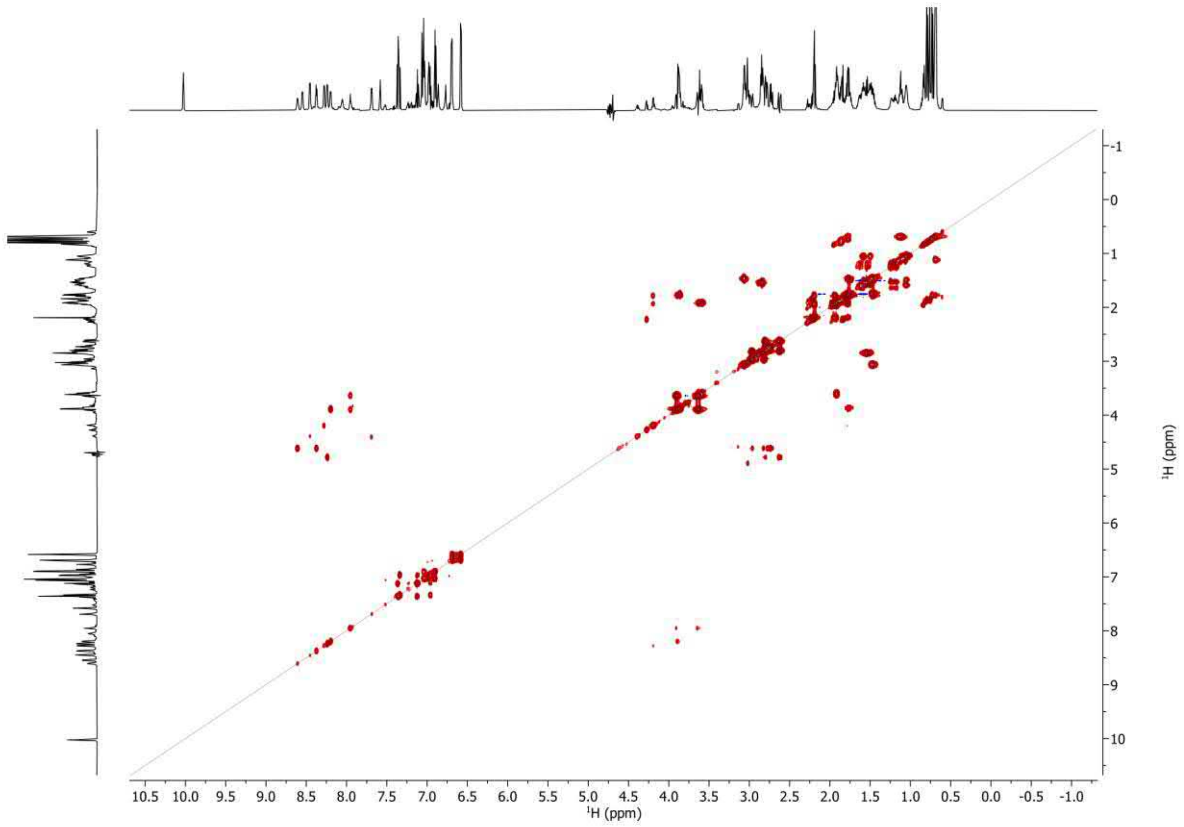

Figure S27. COSY spectrum of compound **1** (800 MHz, H_2O:D_2O 9:1, 25 °C).

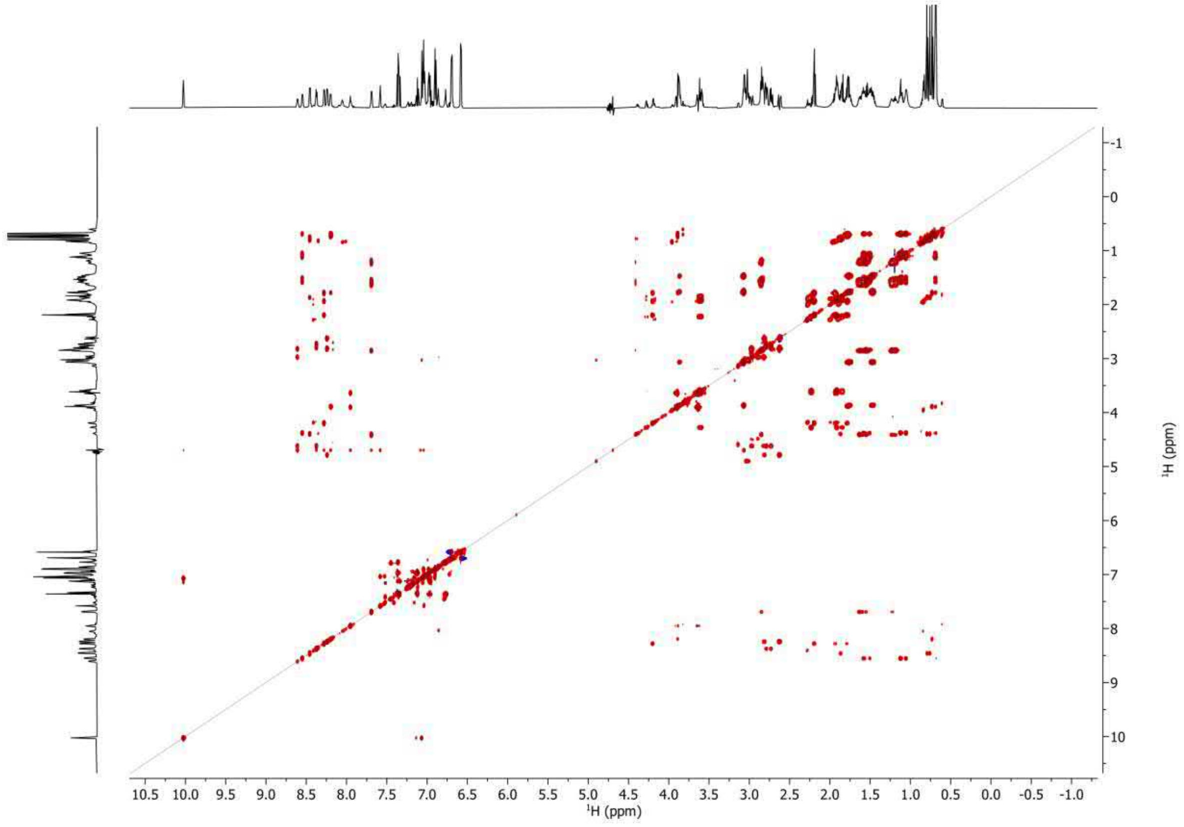

Figure S28. TOCSY spectrum of compound **1** (800 MHz, H_2O:D_2O 9:1, 25 °C).

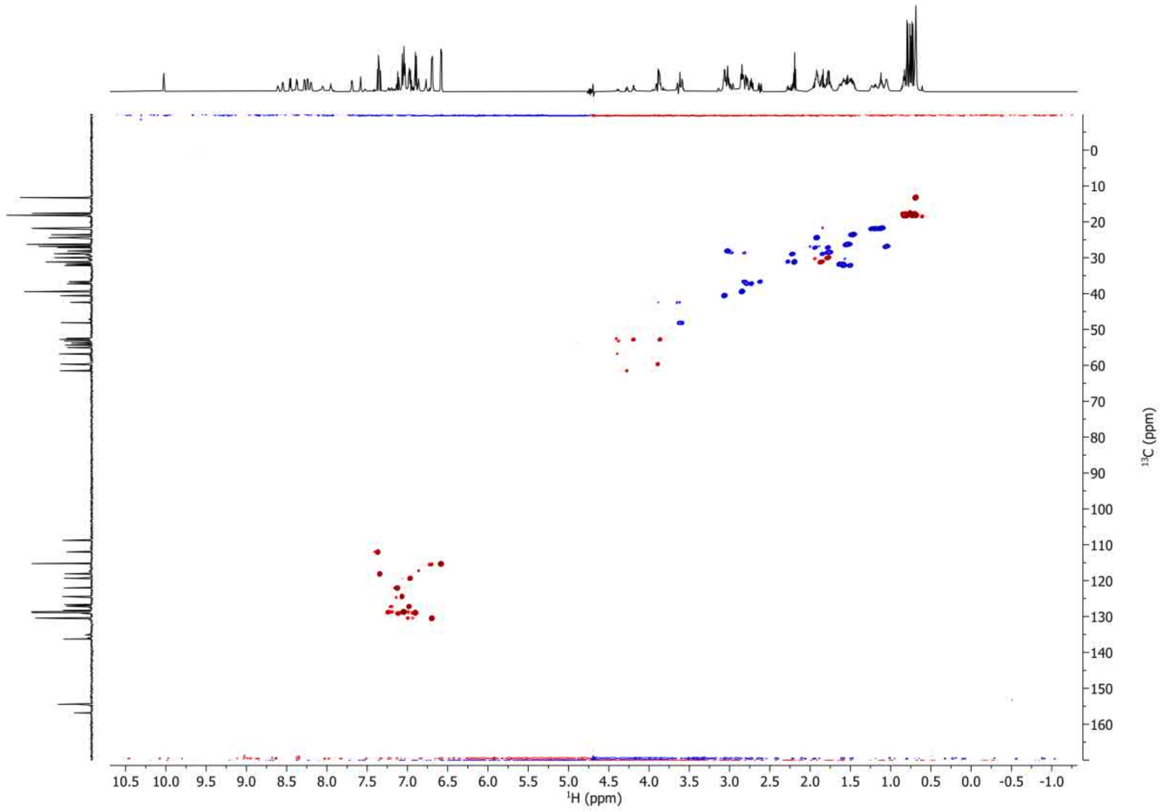

Figure S29. HSQC spectrum of compound **1** (800 MHz, H_2O:D_2O 9:1, 25 °C).

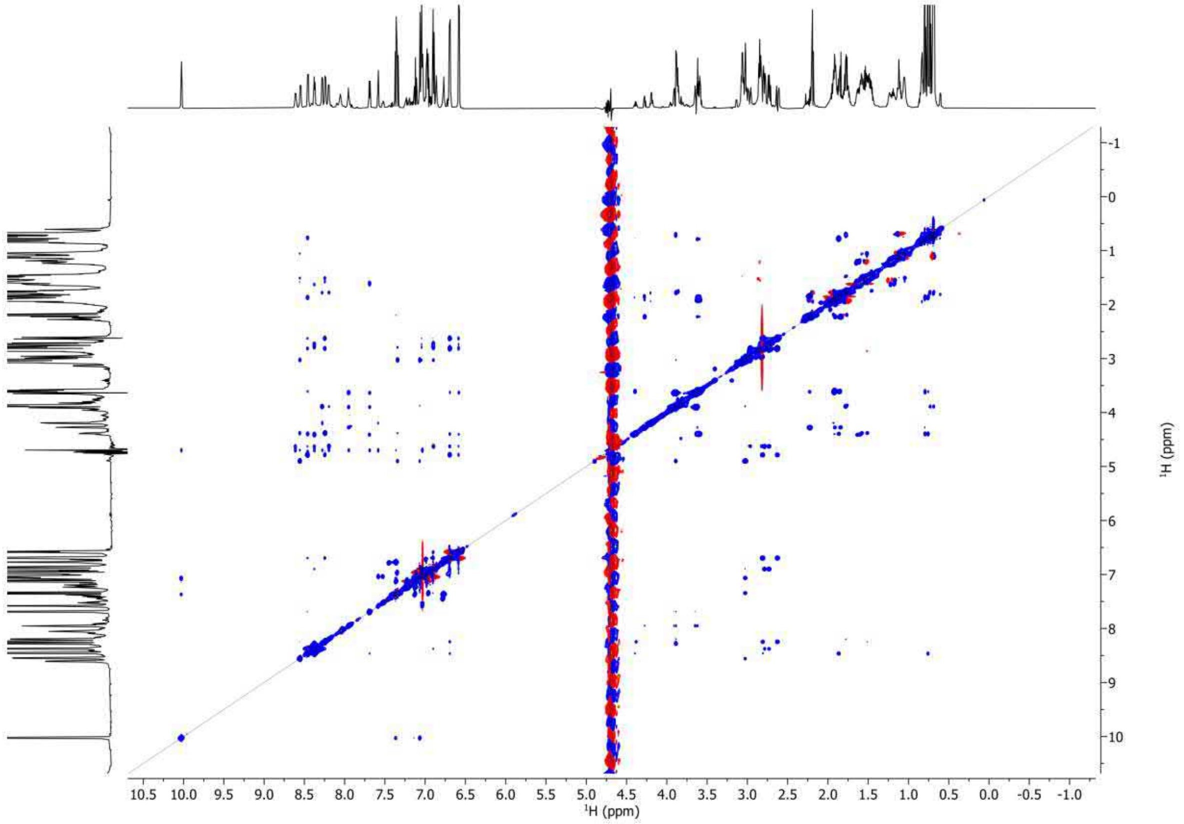

Figure S30. NOESY spectrum of compound **1** (800 MHz, H_2O:D_2O 9:1, 25 °C, t_{mix} = 700 ms).

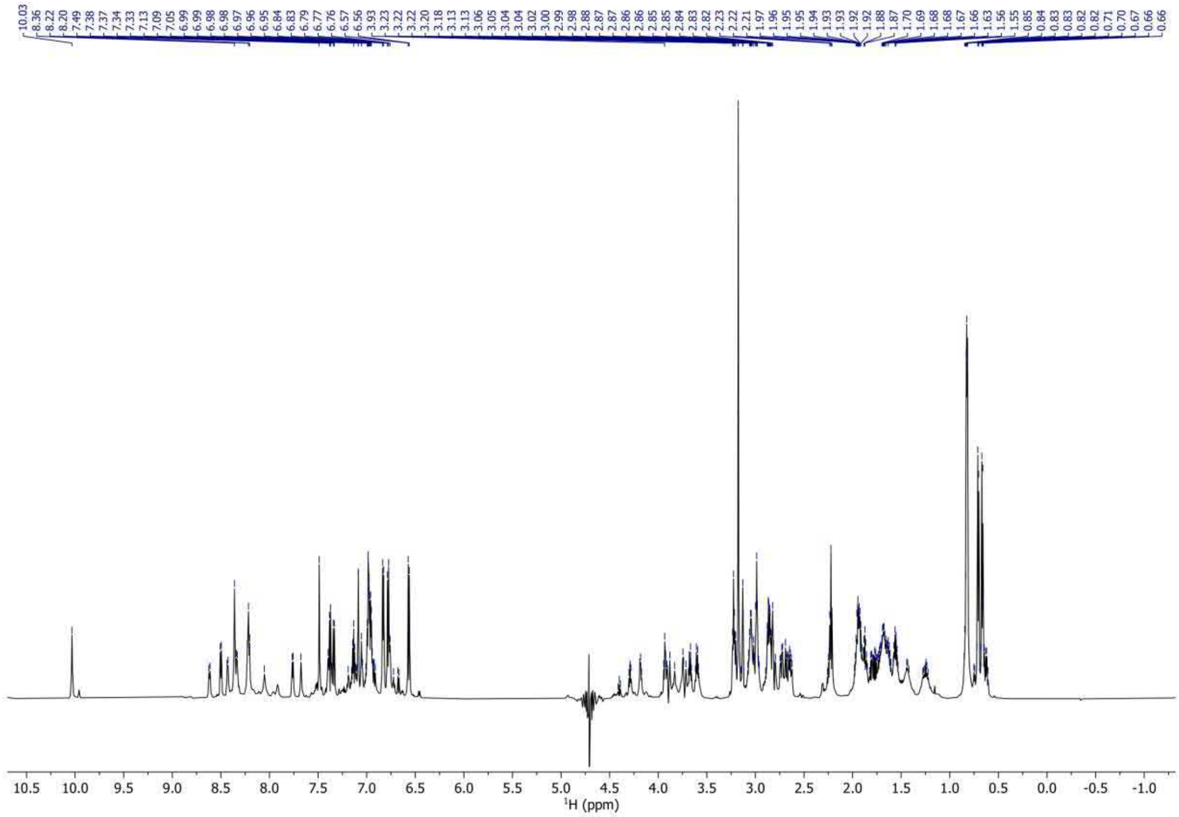

Figure S31. ^{1}H NMR spectrum of compound **2** (800 MHz, H_2O:D_2O 9:1, 25 °C).

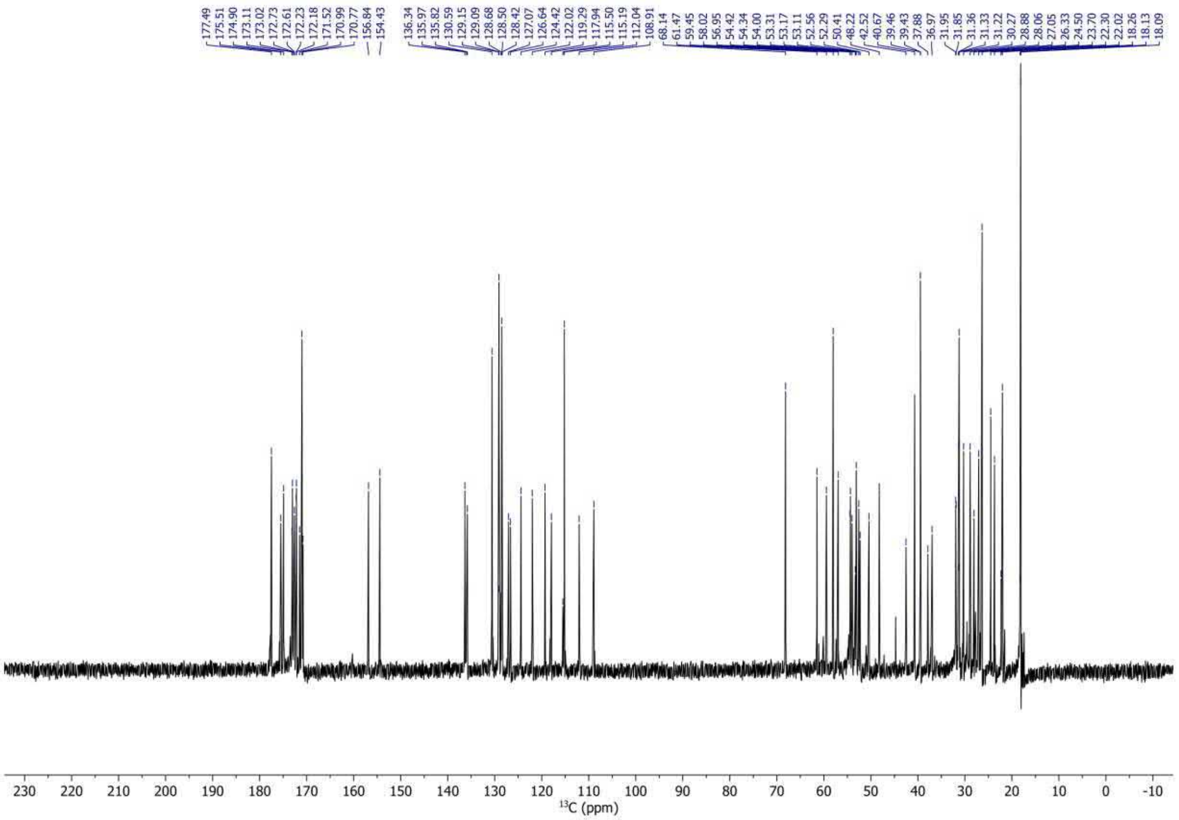

Figure S32. ^{13}C NMR spectrum of compound **2** (201 MHz, H_2O:D_2O 9:1, 25 °C).

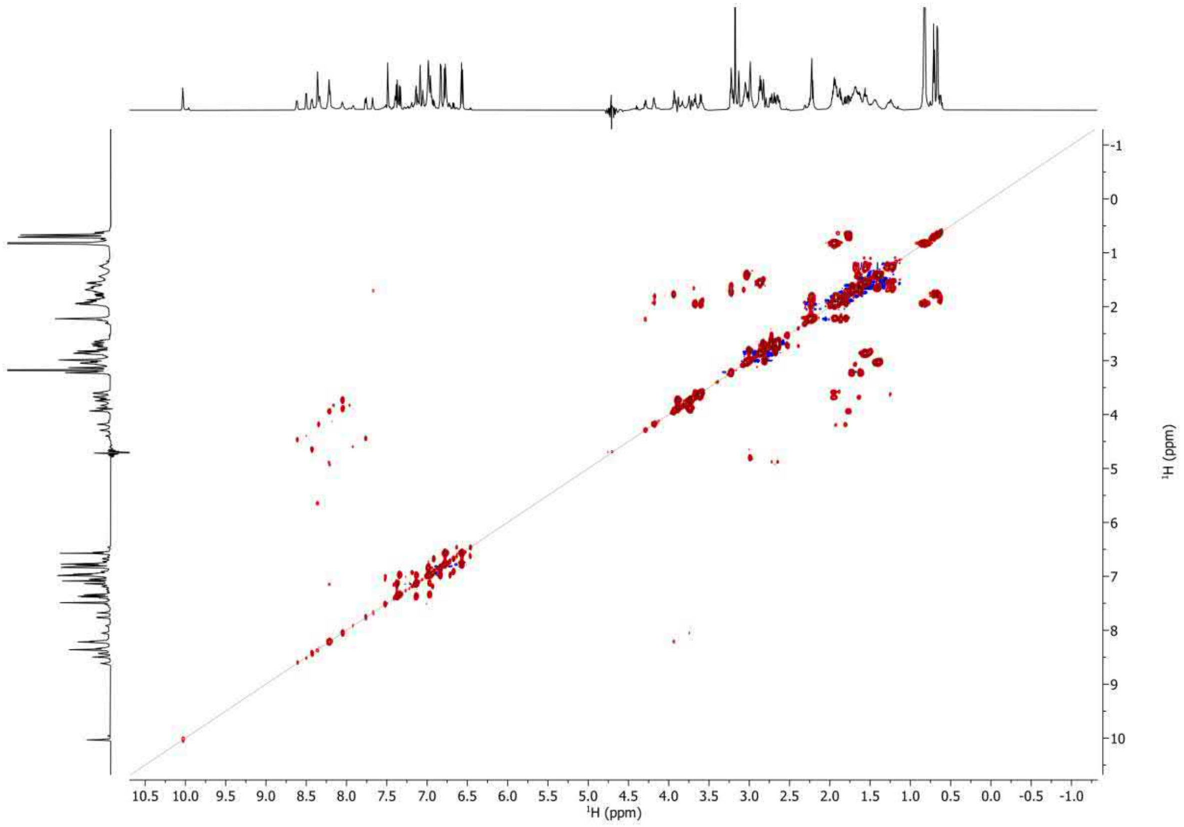

Figure S33. COSY spectrum of compound **2** (800 MHz, H_2O:D_2O 9:1, 25 °C).

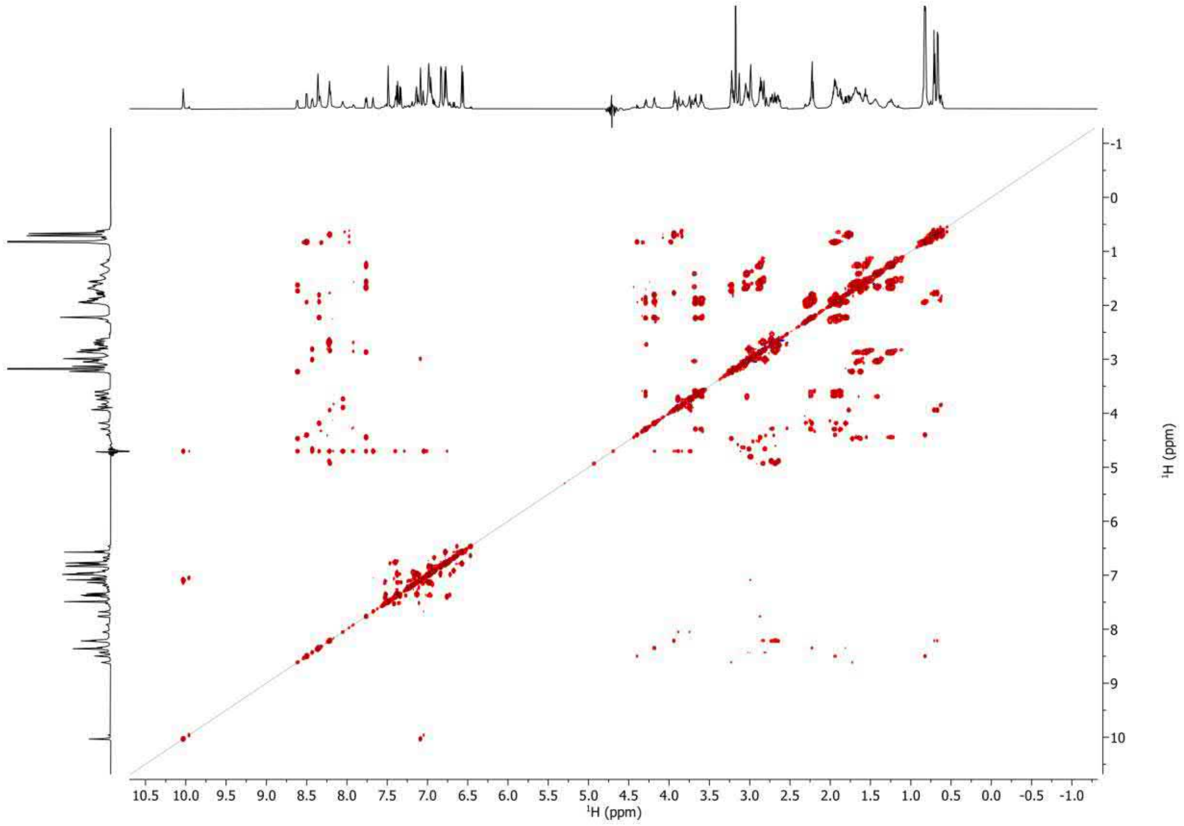

Figure S34. TOCSY spectrum of compound **2** (800 MHz, H_2O:D_2O 9:1, 25 °C).

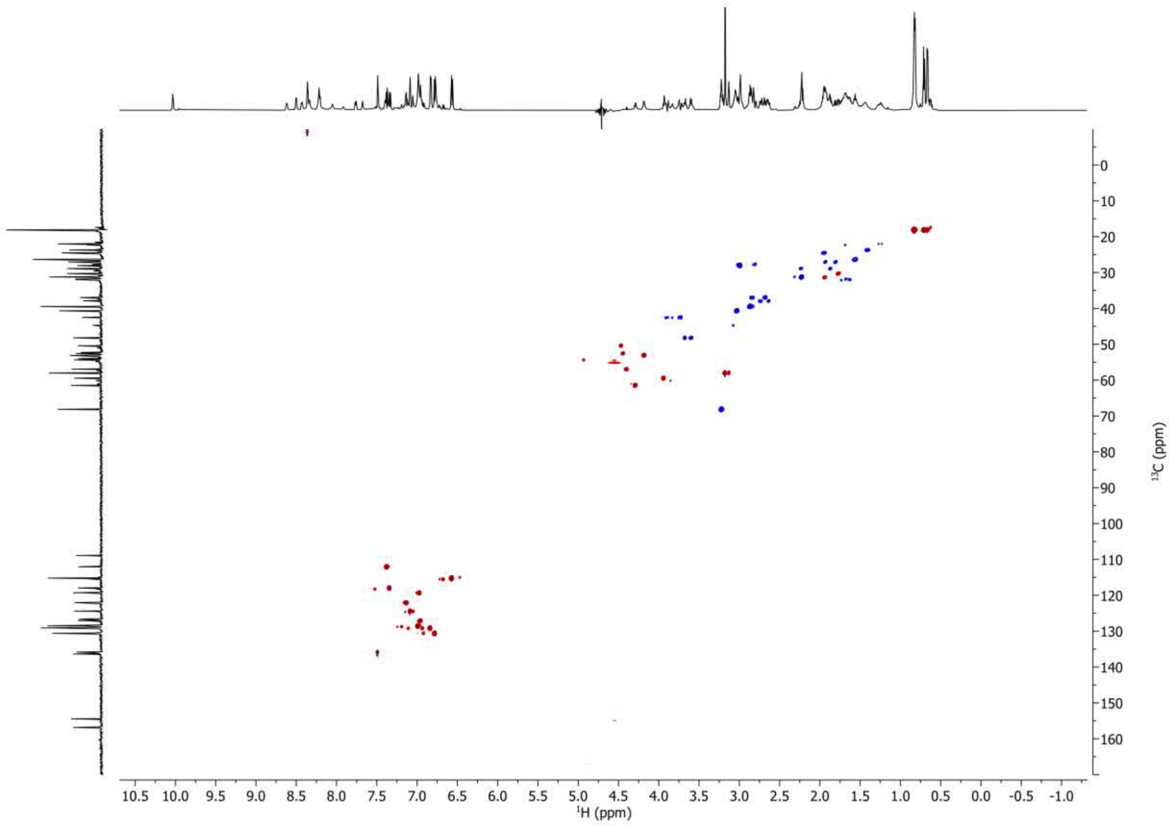

Figure S35. HSQC spectrum of compound **2** (800 MHz, H_2O:D_2O 9:1, 25 °C).

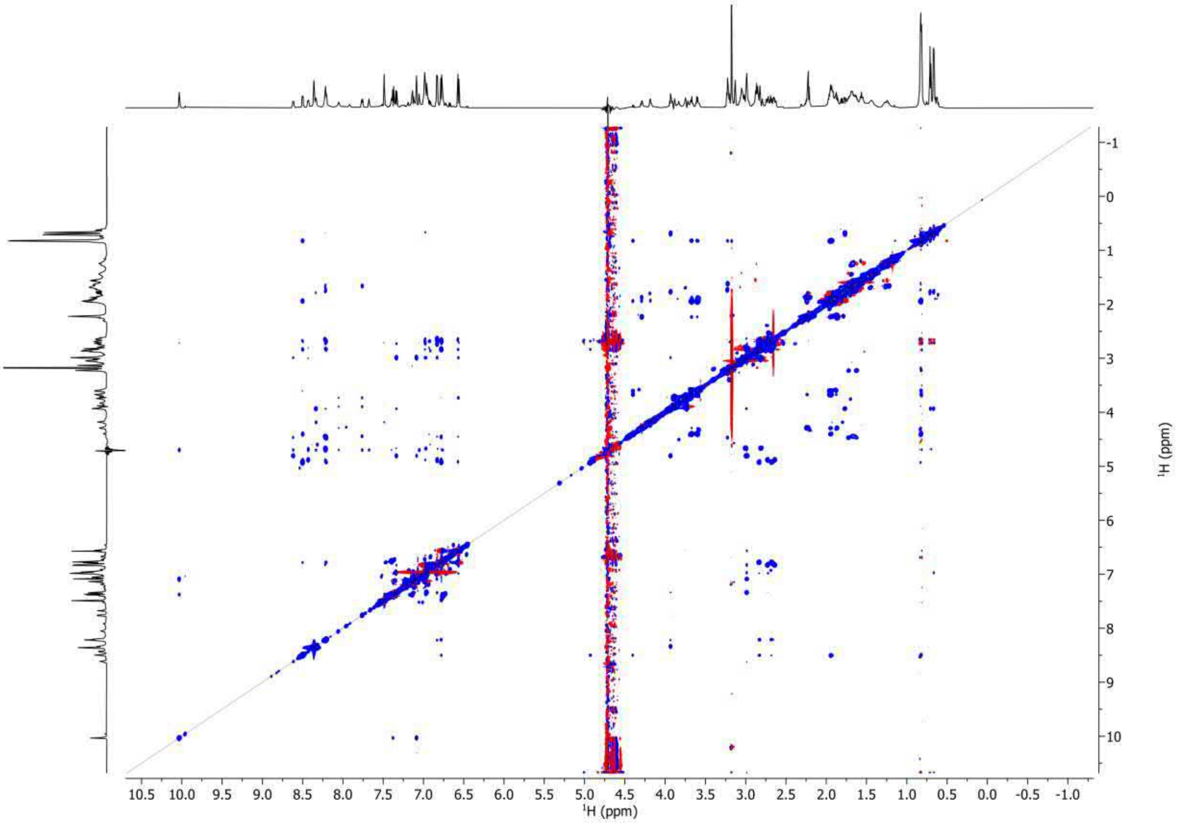

Figure S36. NOESY spectrum of compound **2** (800 MHz, H_2O:D_2O 9:1, 25 °C, t_{mix} = 700 ms).

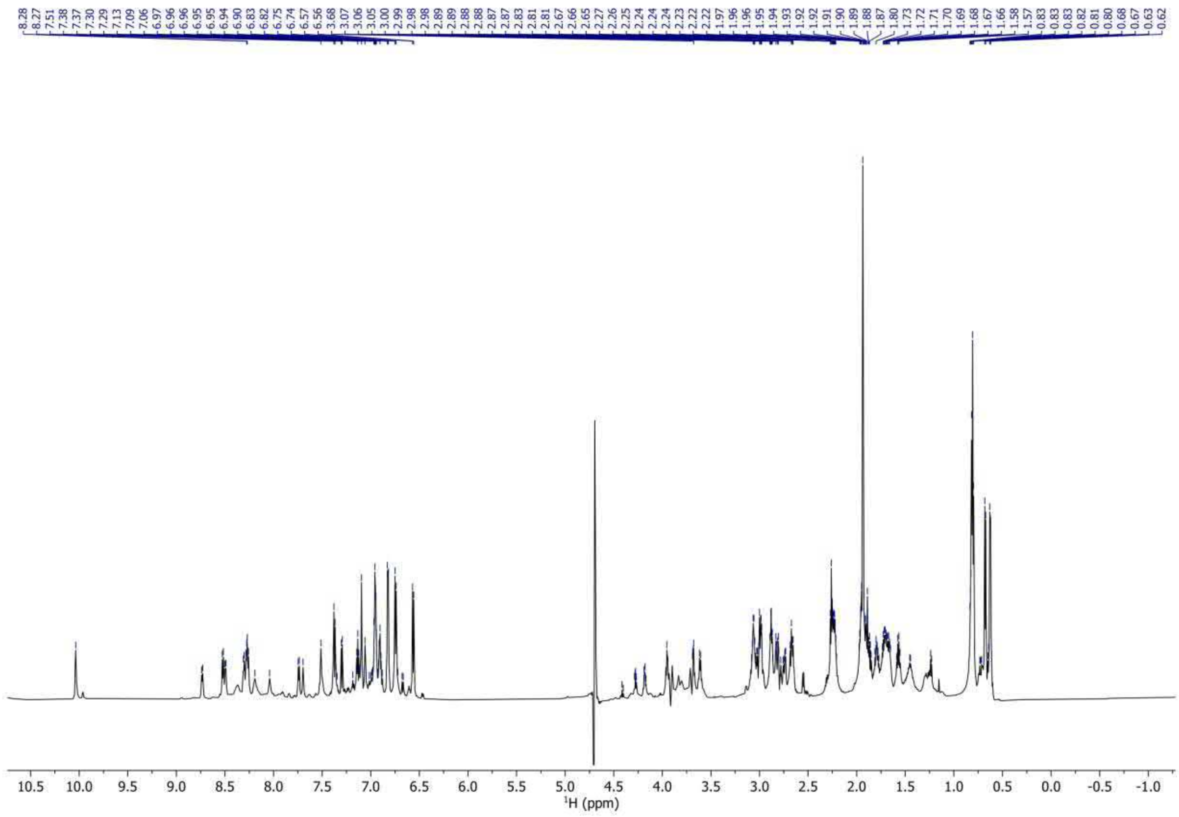

Figure S37. ^{1}H NMR spectrum of compound **3** (800 MHz, H_2O:D_2O 9:1, 25 °C).

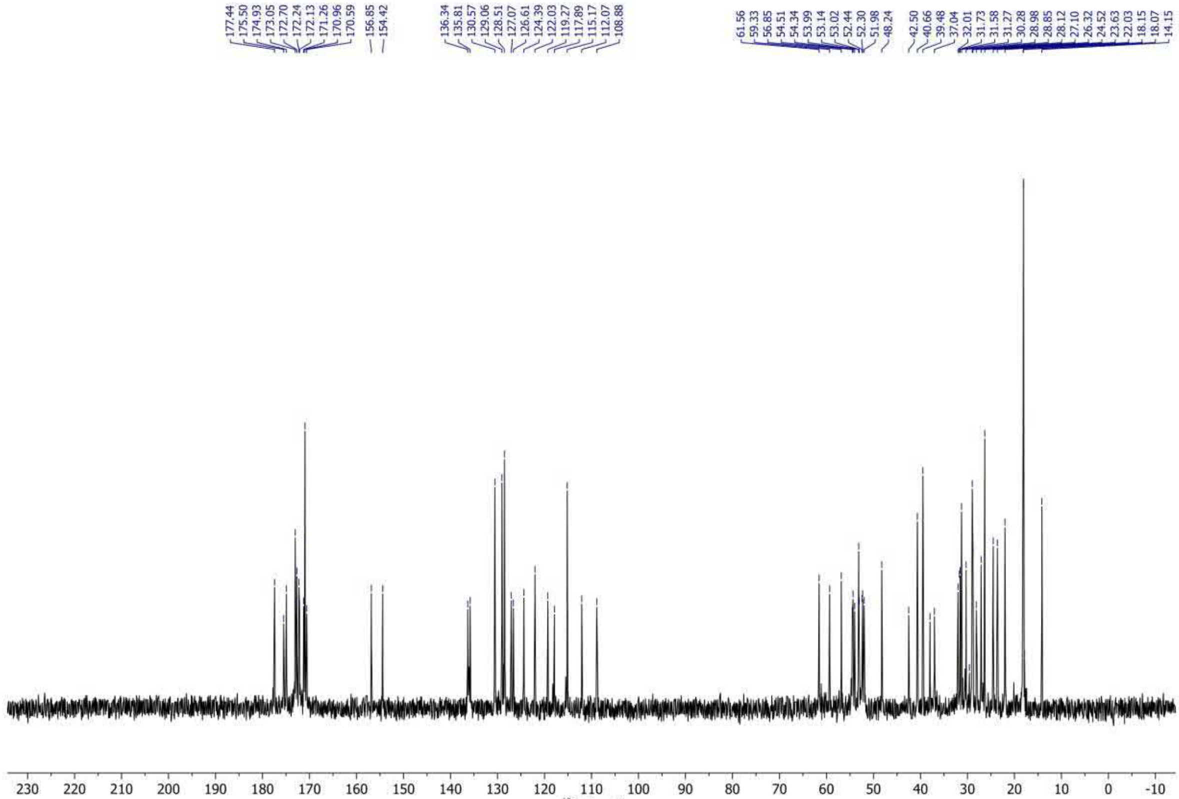

Figure S38. ^{13}C NMR spectrum of compound **3** (201 MHz, H_2O:D_2O 9:1, 25 °C).

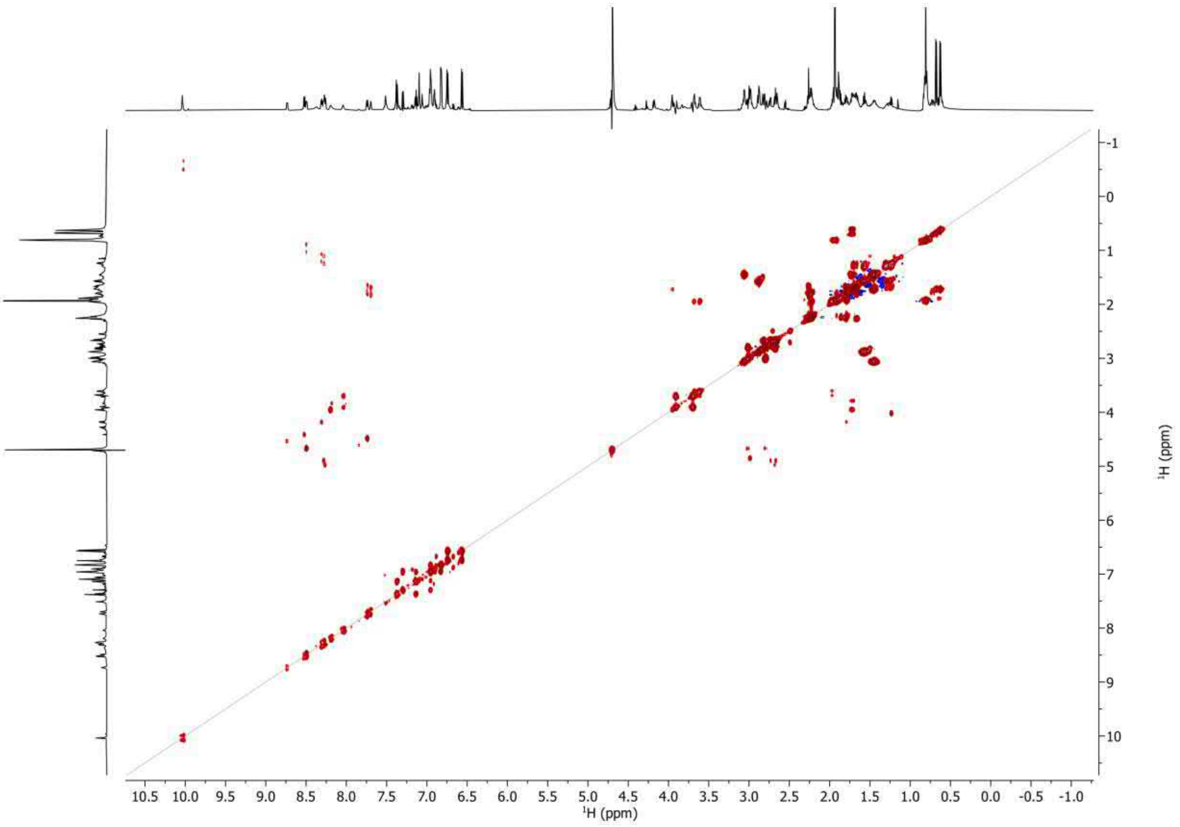

Figure S39. COSY spectrum of compound **3** (800 MHz, H_2O:D_2O 9:1, 25 °C).

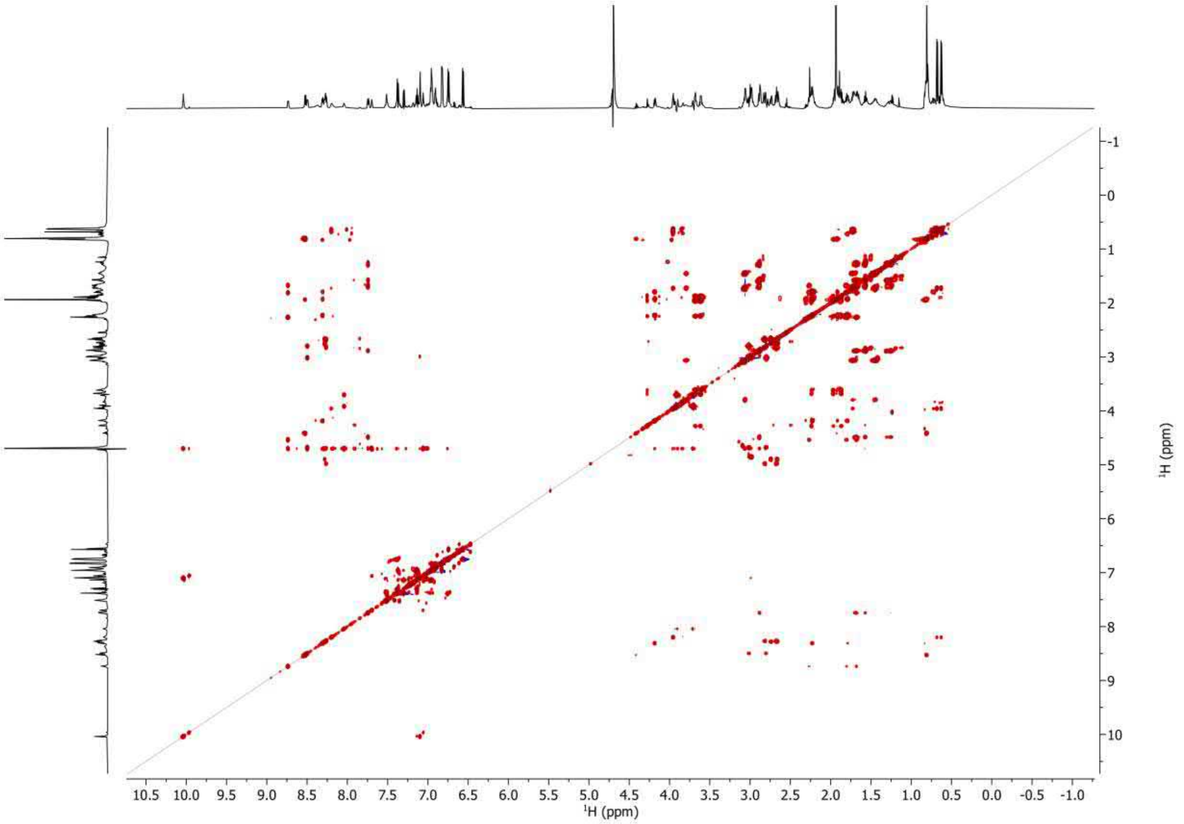

Figure S40. TOCSY spectrum of compound **3** (800 MHz, H_2O:D_2O 9:1, 25 °C).

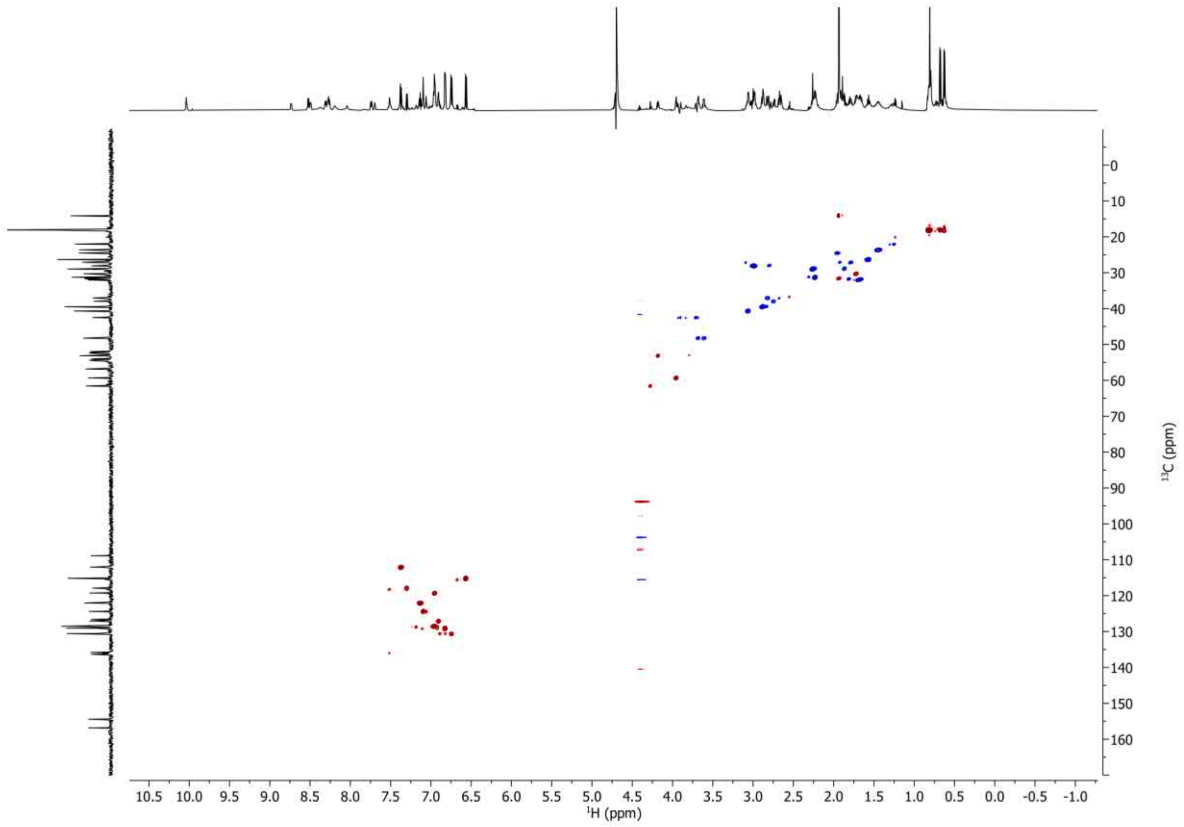

Figure S41. HSQC spectrum of compound **3** (800 MHz, H_2O:D_2O 9:1, 25 °C).

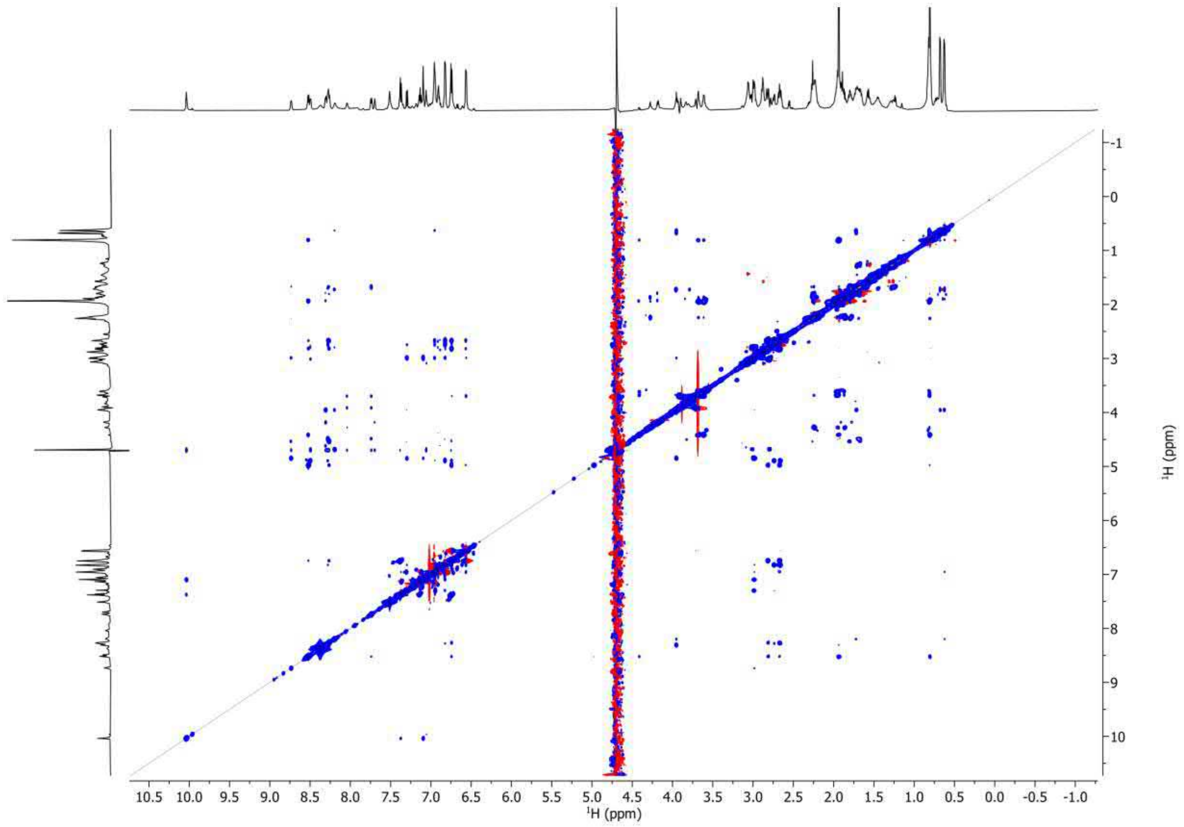

Figure S42. NOESY spectrum of compound **3** (800 MHz, H_2O:D_2O 9:1, 25 °C, t_{mix} = 700 ms).

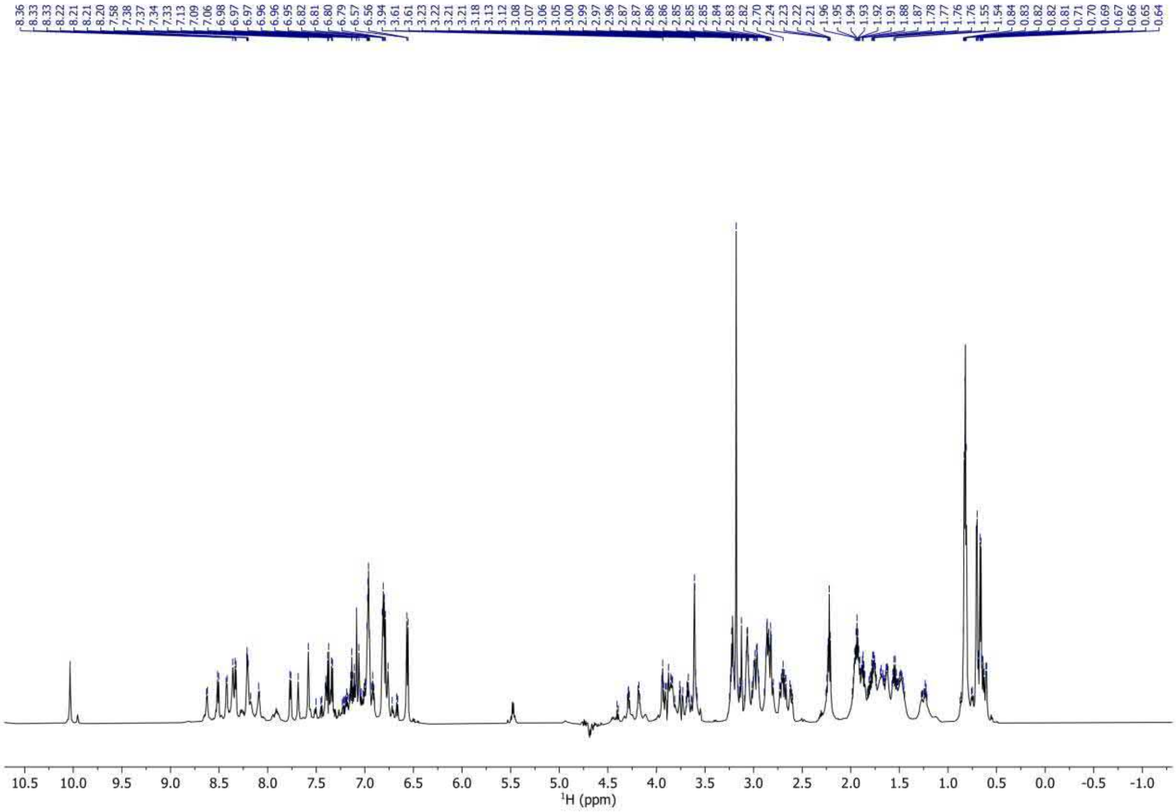

Figure S43. ^{1}H NMR spectrum of compound **4** (800 MHz, H_2O:D_2O 9:1, 25 °C).

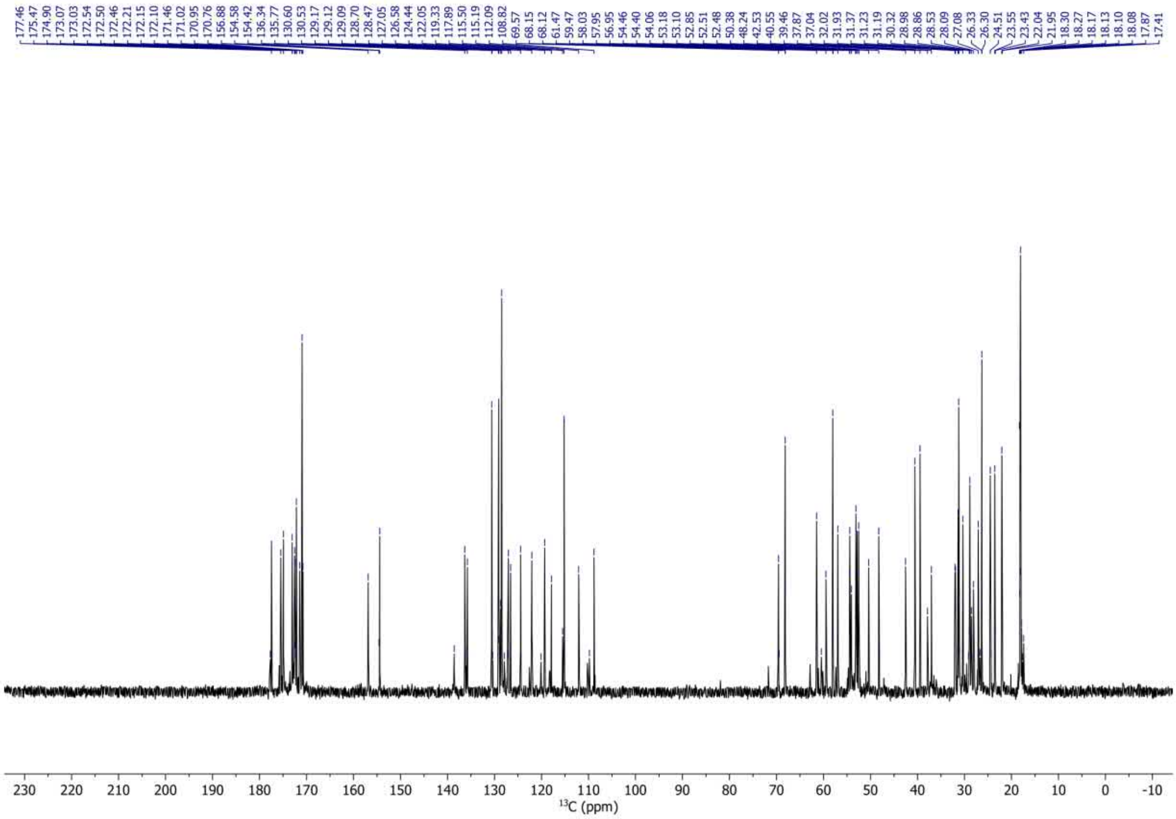

Figure S44. ^{13}C NMR spectrum of compound **4** (201 MHz, H_2O:D_2O 9:1, 25 °C).

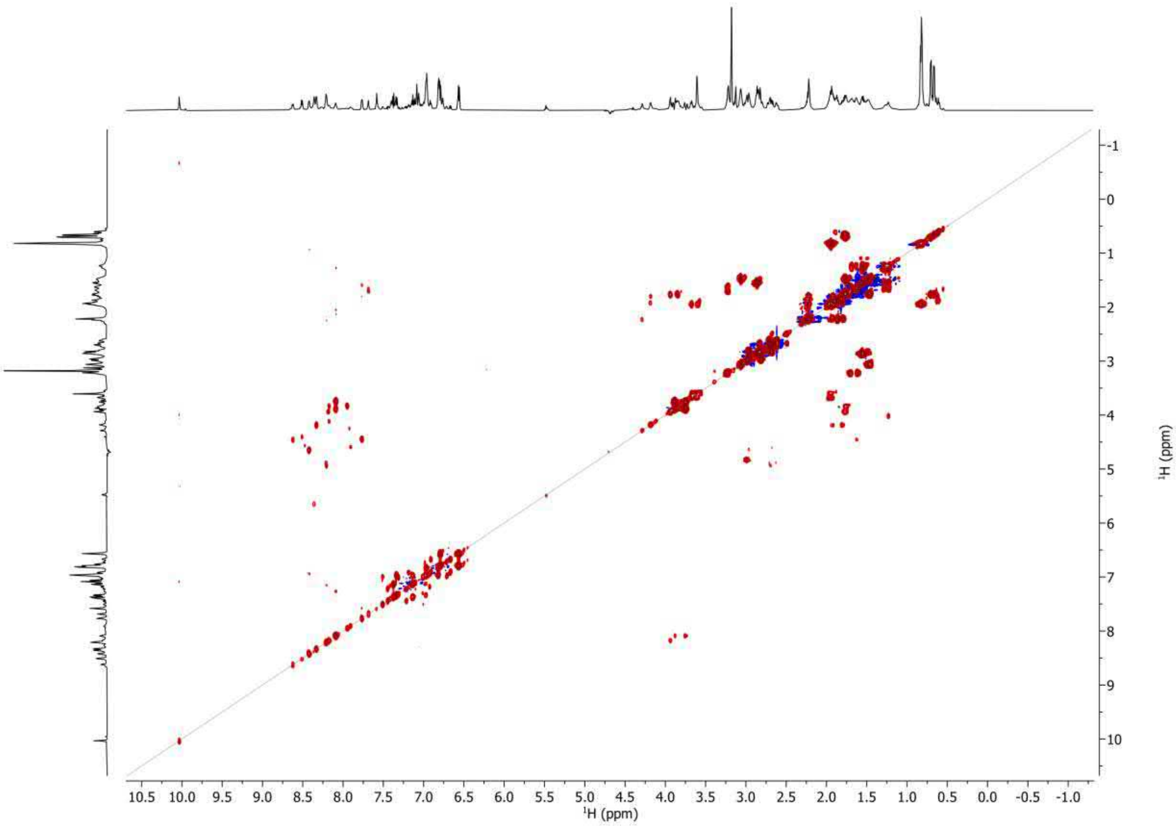

Figure S45. COSY spectrum of compound **4** (800 MHz, H_2O:D_2O 9:1, 25 °C).

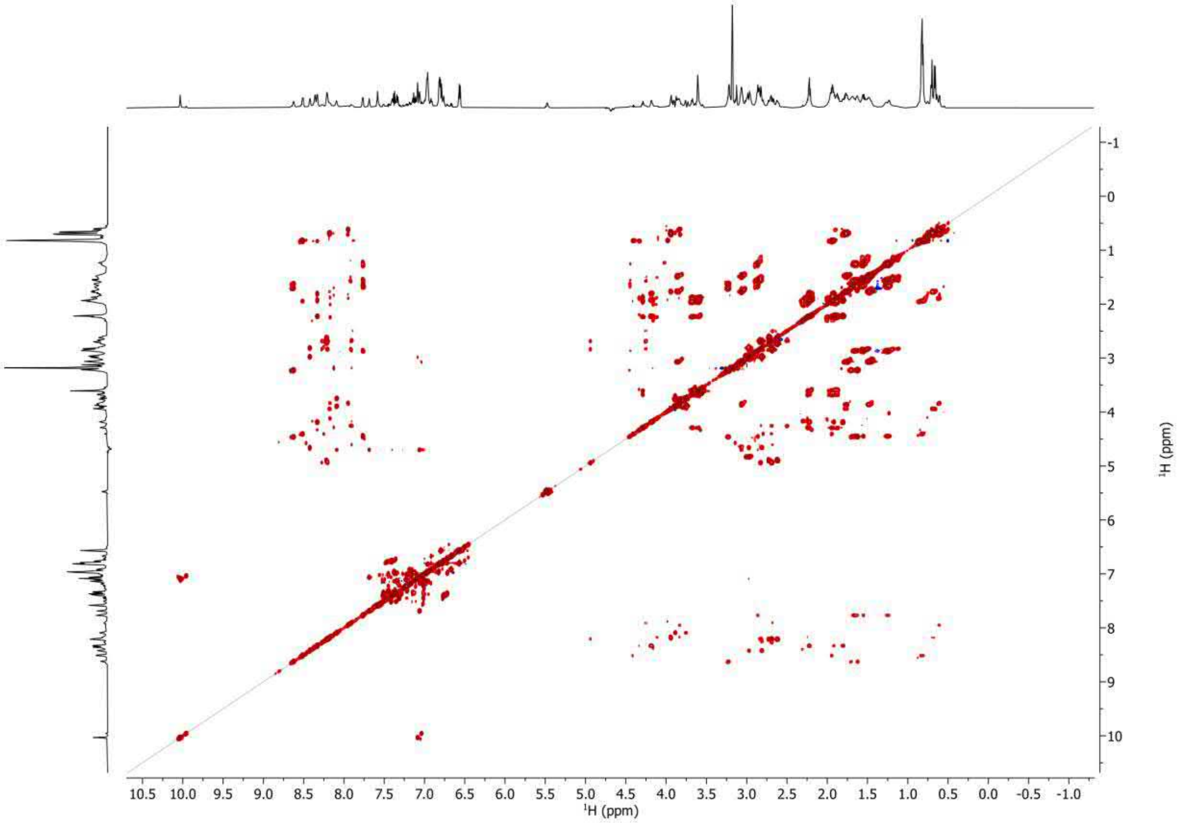

Figure S46. TOCSY spectrum of compound **4** (800 MHz, H_2O:D_2O 9:1, 25 °C).

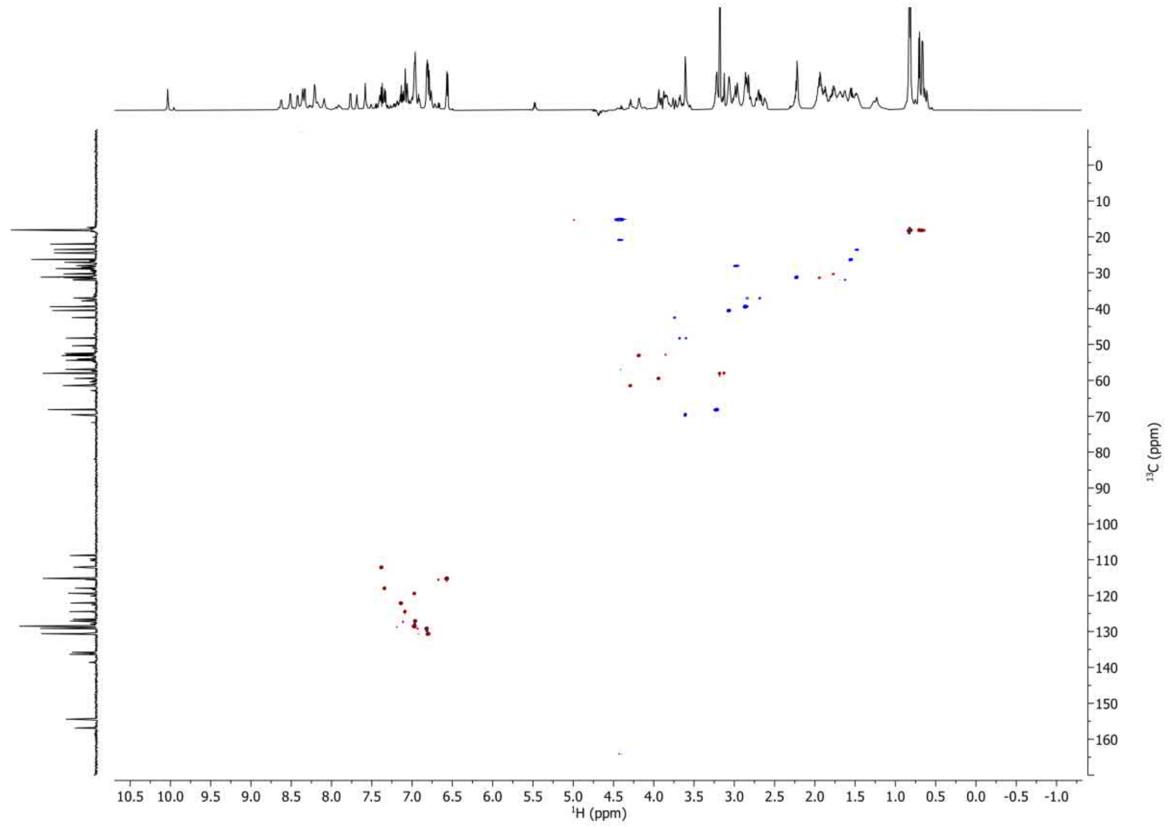

Figure S47. HSQC spectrum of compound **4** (800 MHz, H_2O:D_2O 9:1, 25 °C).

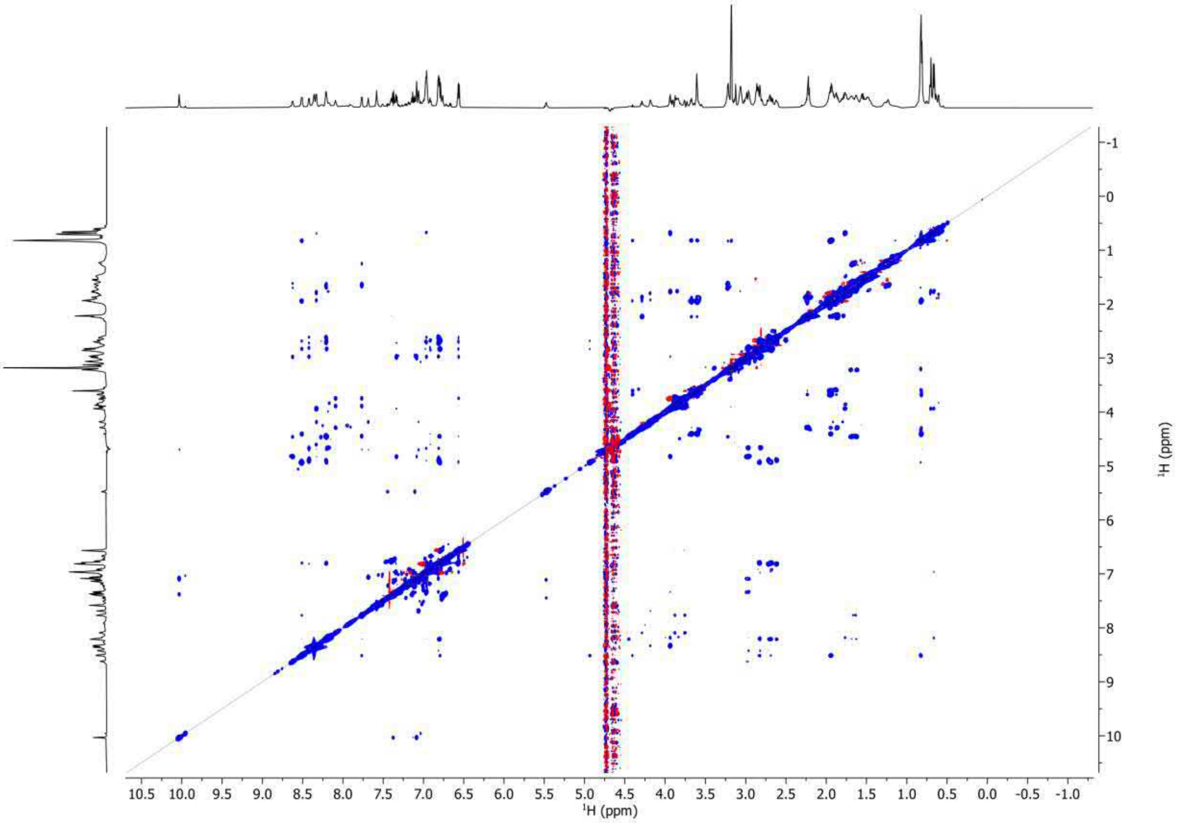

Figure S48. NOESY spectrum of compound **4** (800 MHz, H_2O:D_2O 9:1, 25 °C, t_{mix} = 700 ms).

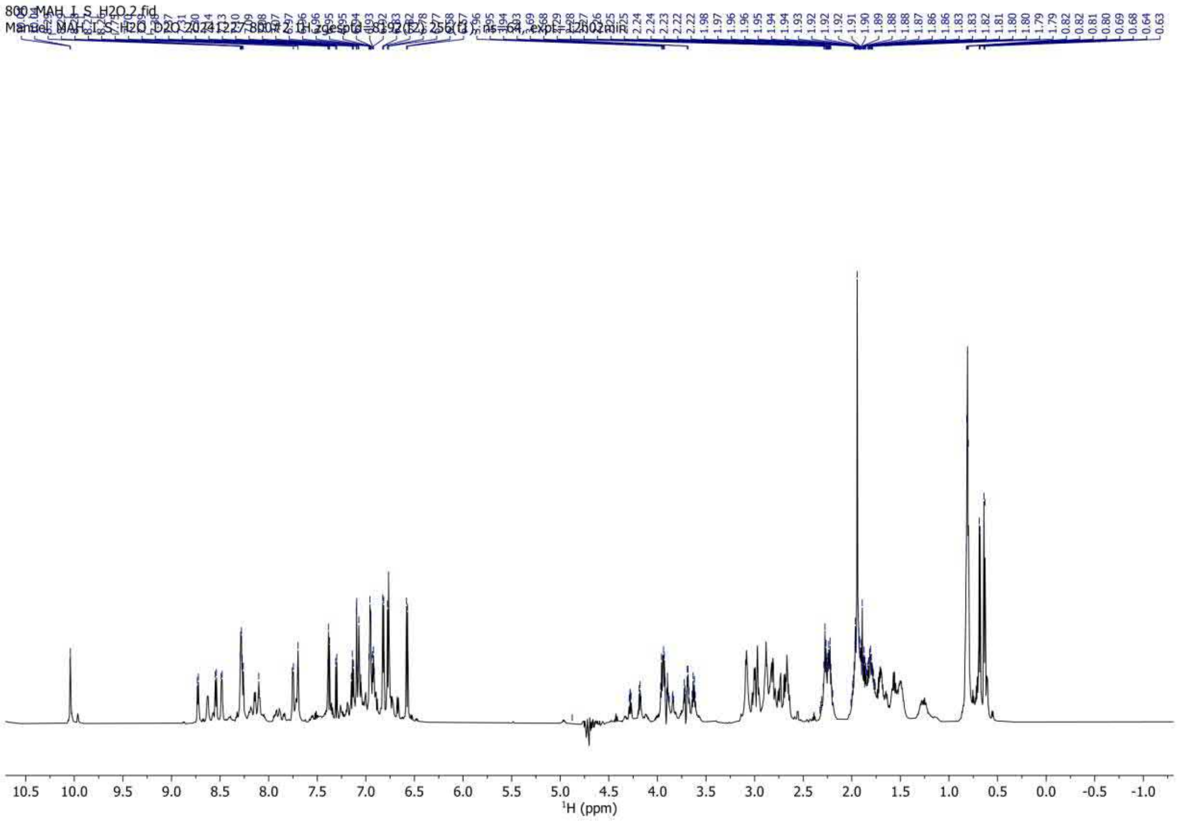

Figure S49. ^{1}H NMR spectrum of compound **5** (800 MHz, H_2O:D_2O 9:1, 25 °C).

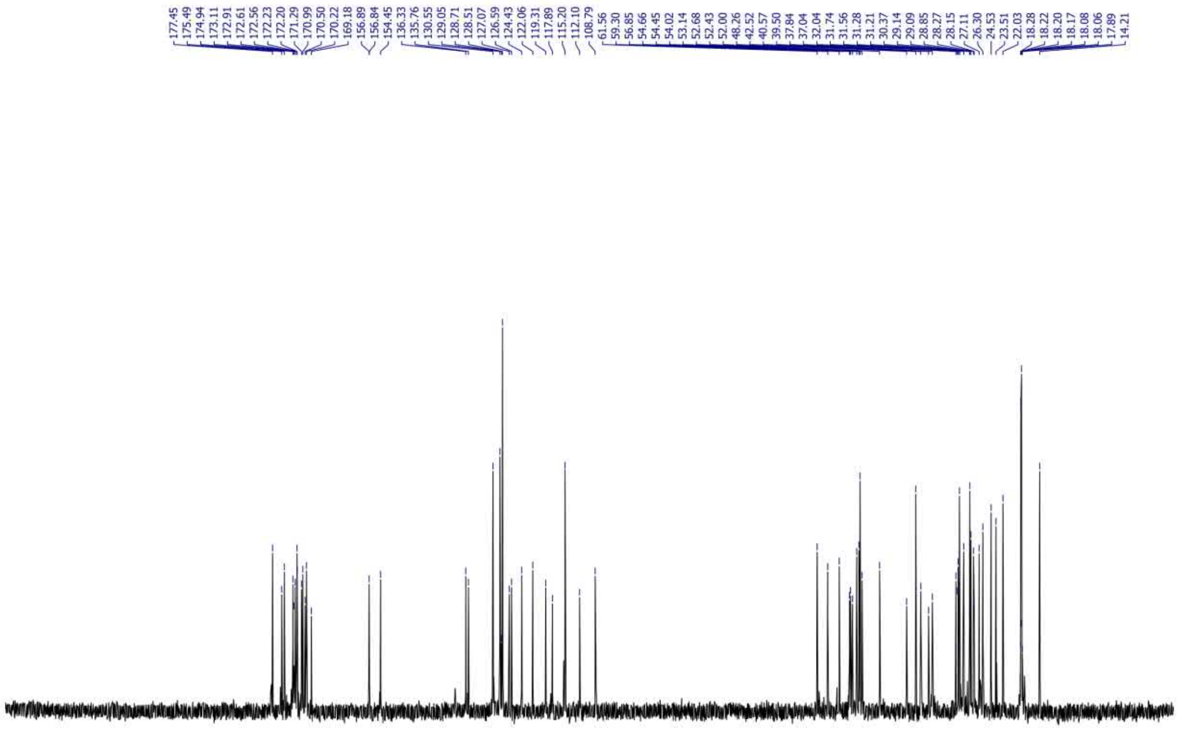

Figure S50. ^{13}C NMR spectrum of compound **5** (201 MHz, H_2O:D_2O 9:1, 25 °C).

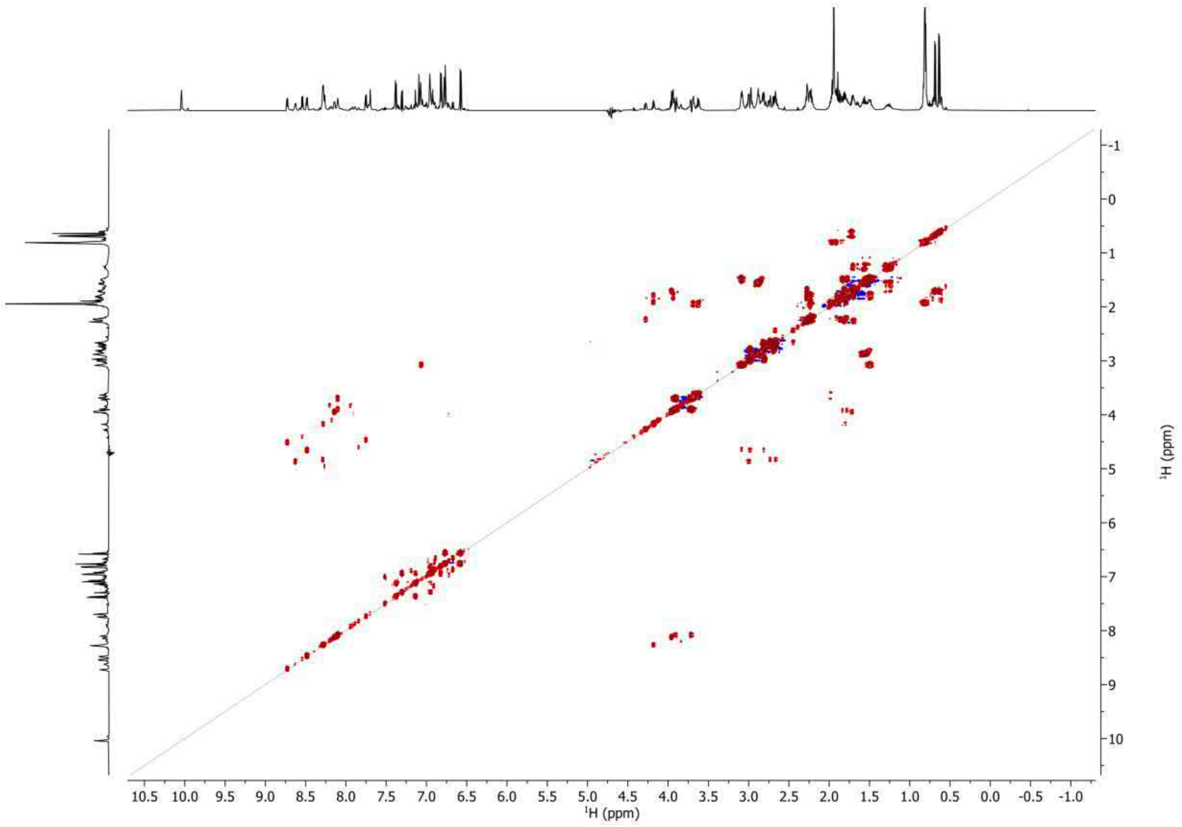

Figure S51. COSY spectrum of compound **5** (800 MHz, H_2O:D_2O 9:1, 25 °C).

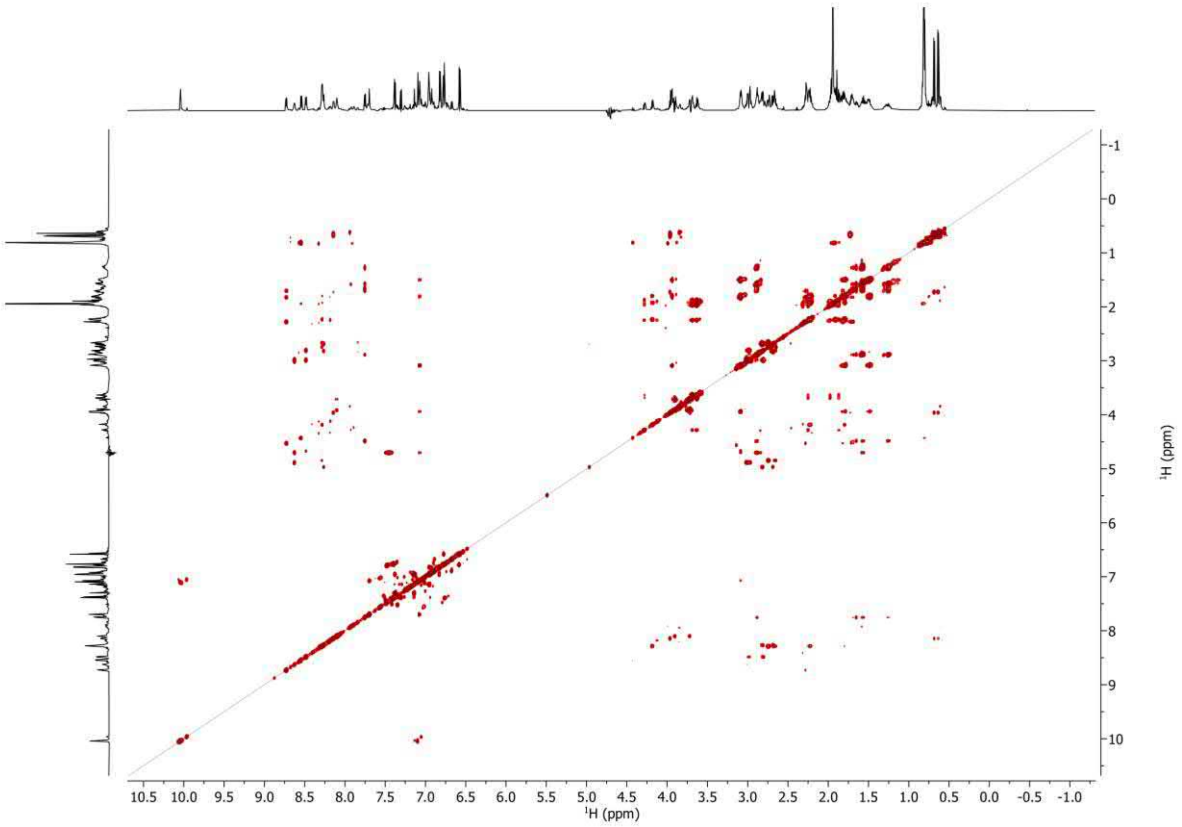

Figure S52. TOCSY spectrum of compound **5** (800 MHz, H_2O:D_2O 9:1, 25 °C).

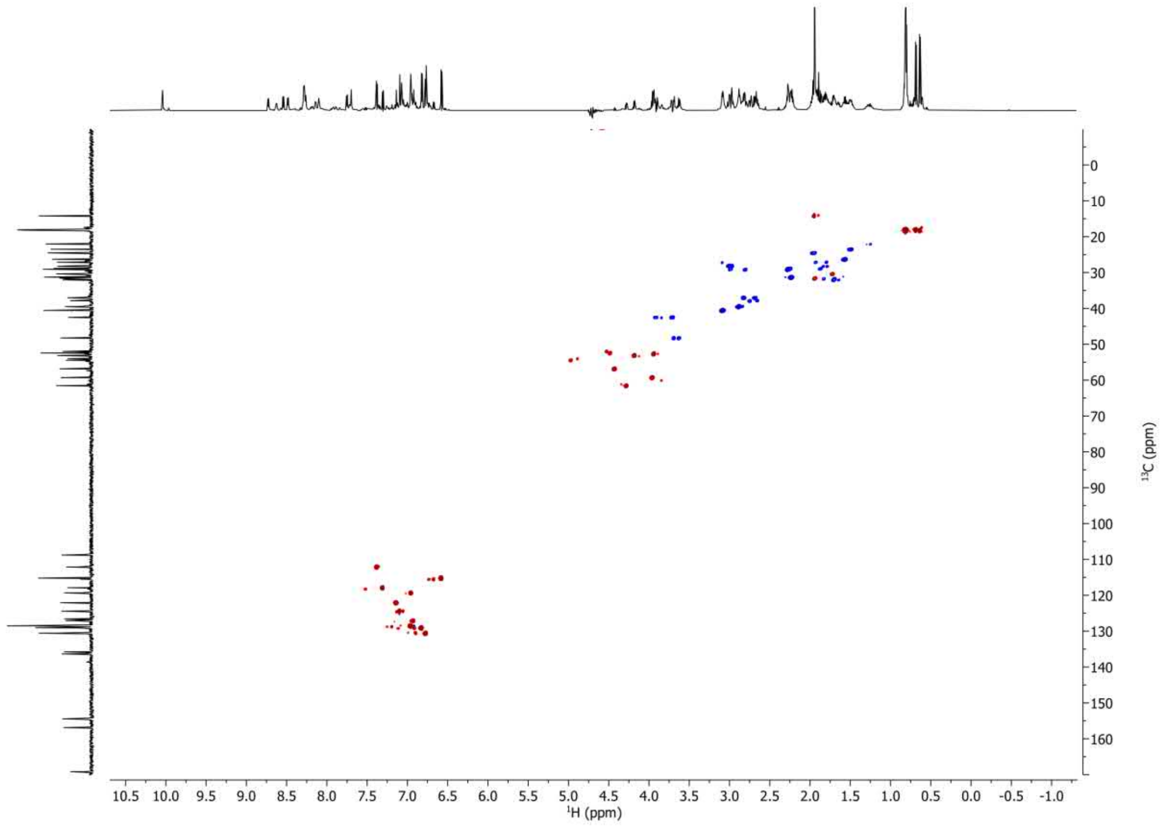

Figure S53. HSQC spectrum of compound **5** (800 MHz, H_2O:D_2O 9:1, 25 °C).

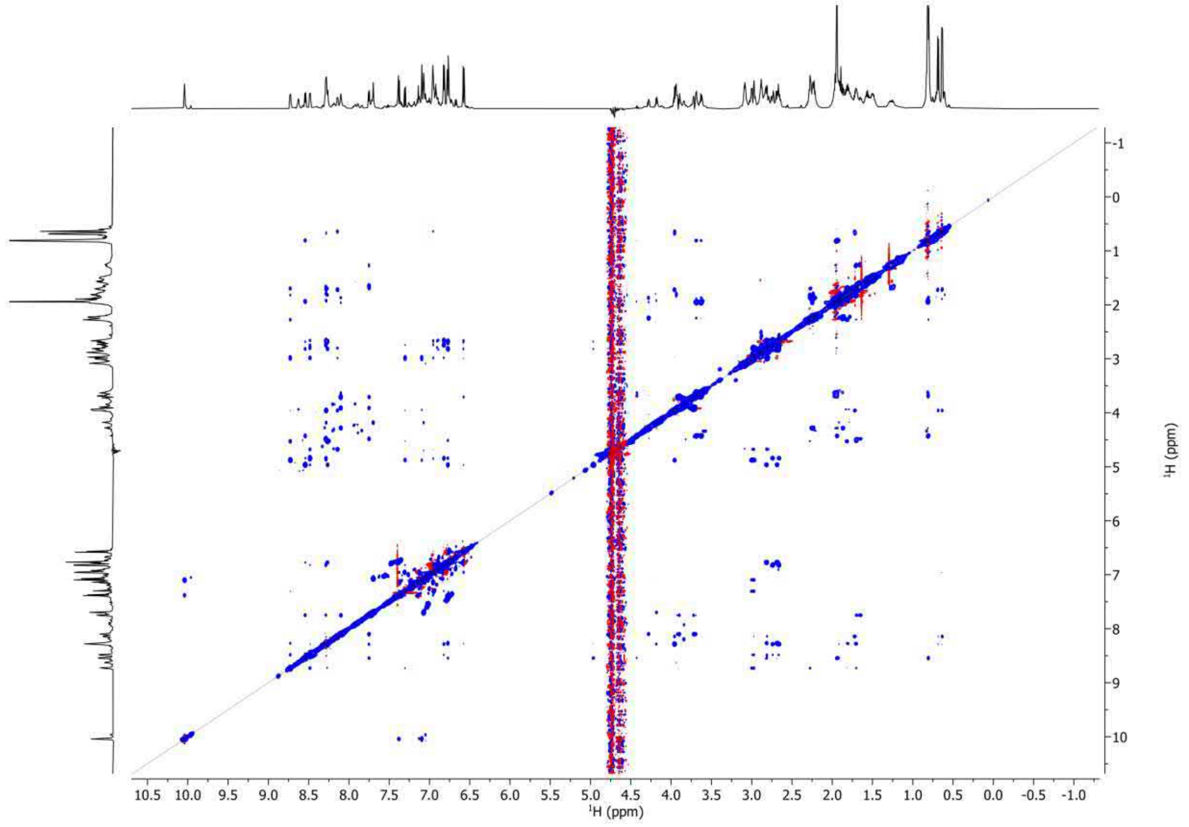

Figure S54. NOESY spectrum of compound **5** (800 MHz, H_2O:D_2O 9:1, 25 °C, t_{mix} = 700 ms).

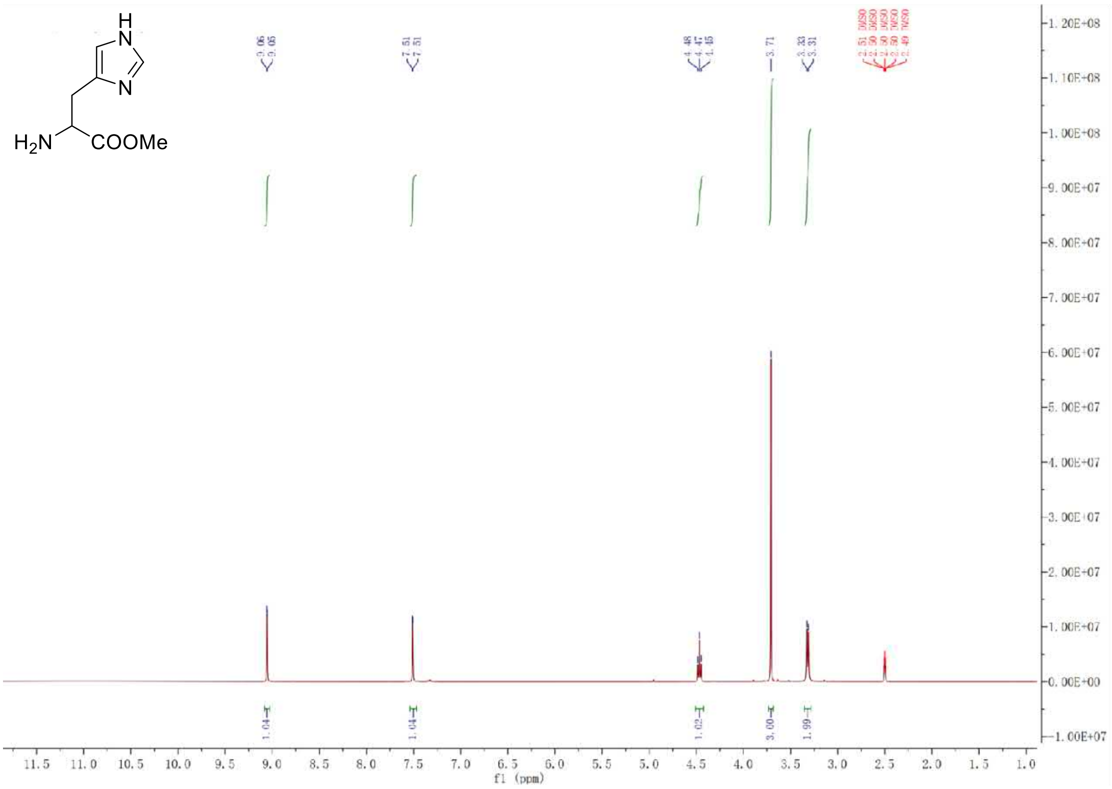

Figure S55. ^{1}H NMR spectrum of methyl L-histidinate (**6**)(400 MHz, CD_3OD, 25 °C)

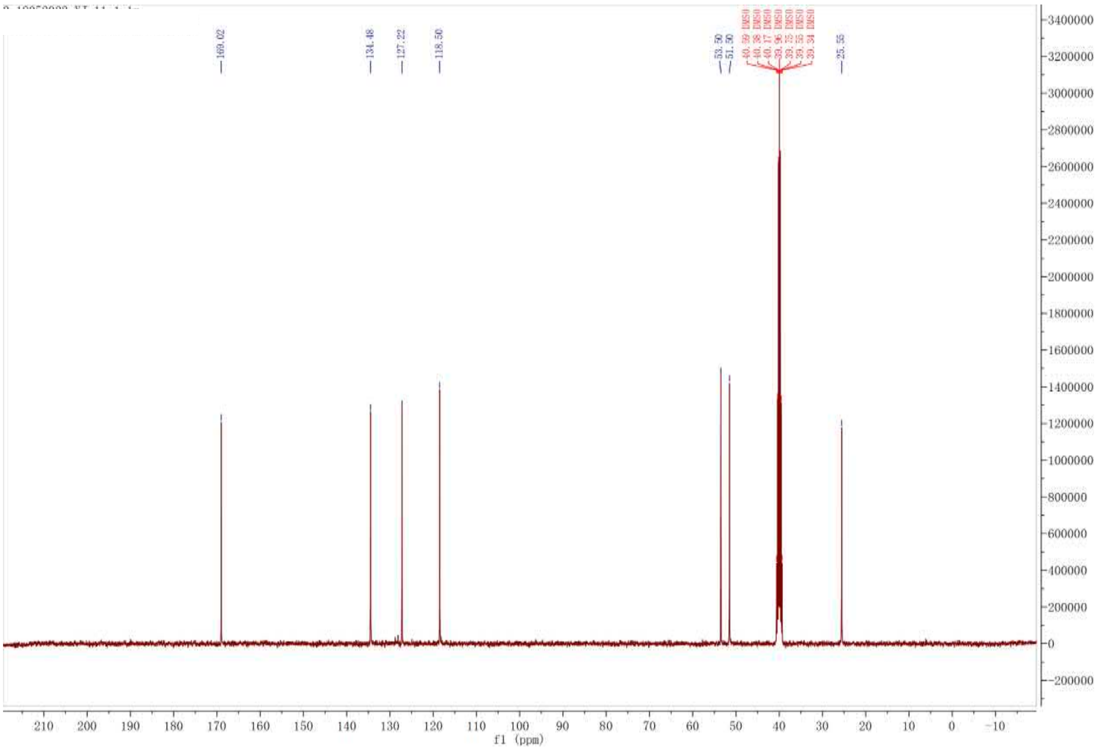

Figure S56. ^{13}C NMR spectrum of methyl L-histidinate (**6**)(100 MHz, CD_3OD, 25 °C)

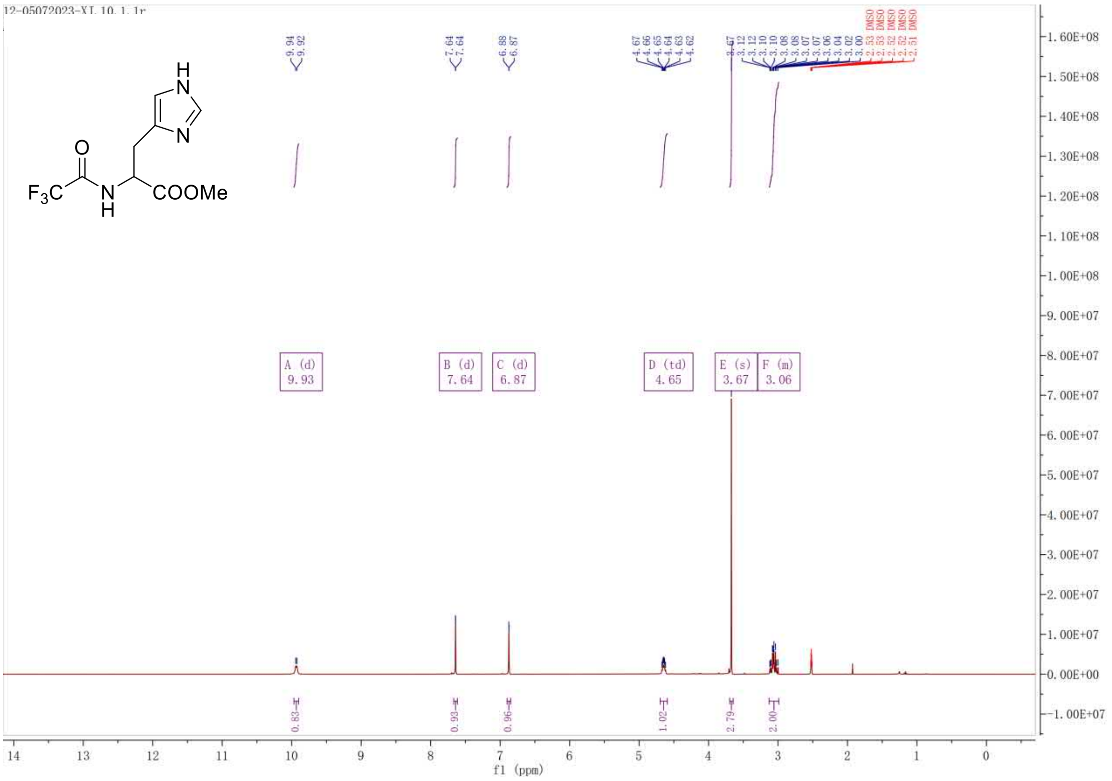

Figure S57. ^{1}H NMR spectrum of methyl (2,2,2-trifluoroacetyl)-L-histidinate (**7**) (400 MHz, CD_3OD, 25 °C).

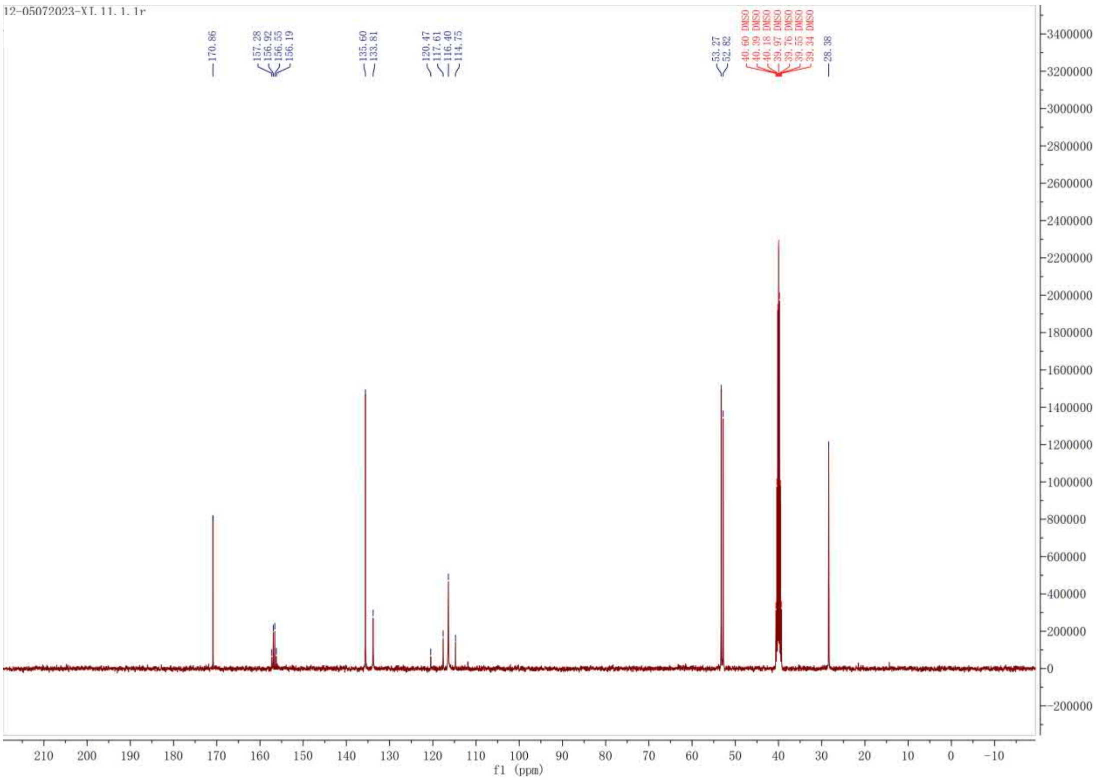

Figure S58. ^{13}C NMR spectrum of compound methyl (2,2,2-trifluoroacetyl)-L-histidinate (**7**) (100 MHz, CD_3OD, 25 °C)

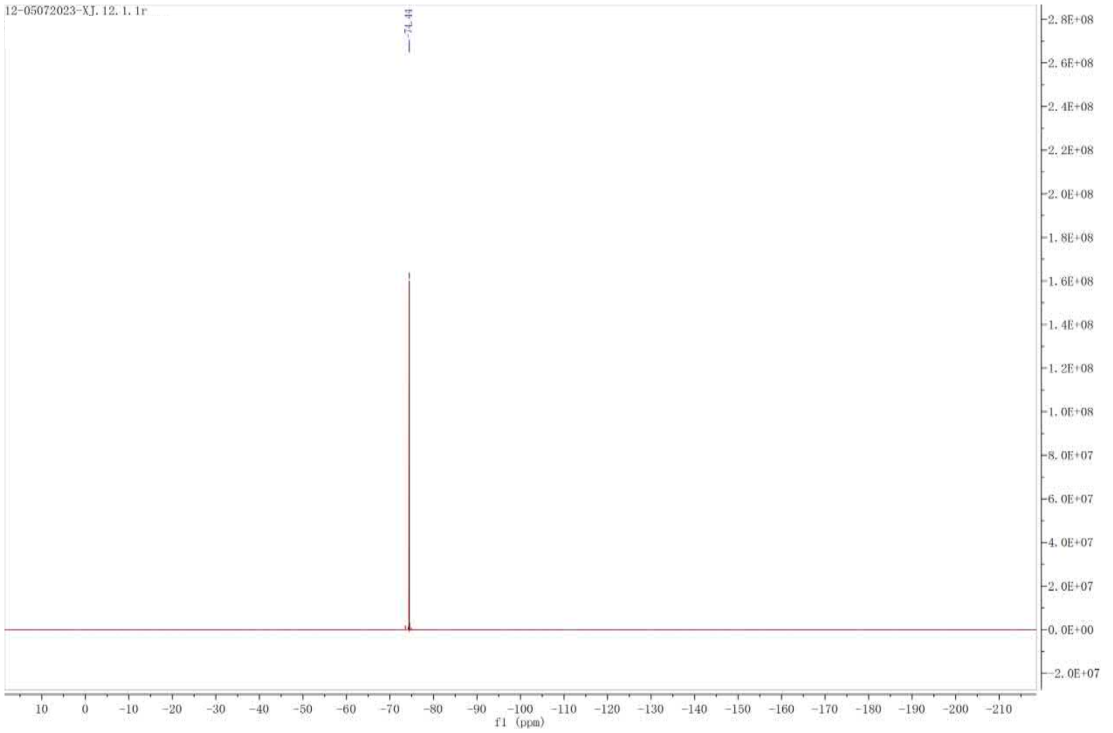

Figure S59. ^{19}F NMR spectrum of methyl (2,2,2-trifluoroacetyl)-L-histidinate (**7**) (376 MHz, CD_3OD, 25 °C).

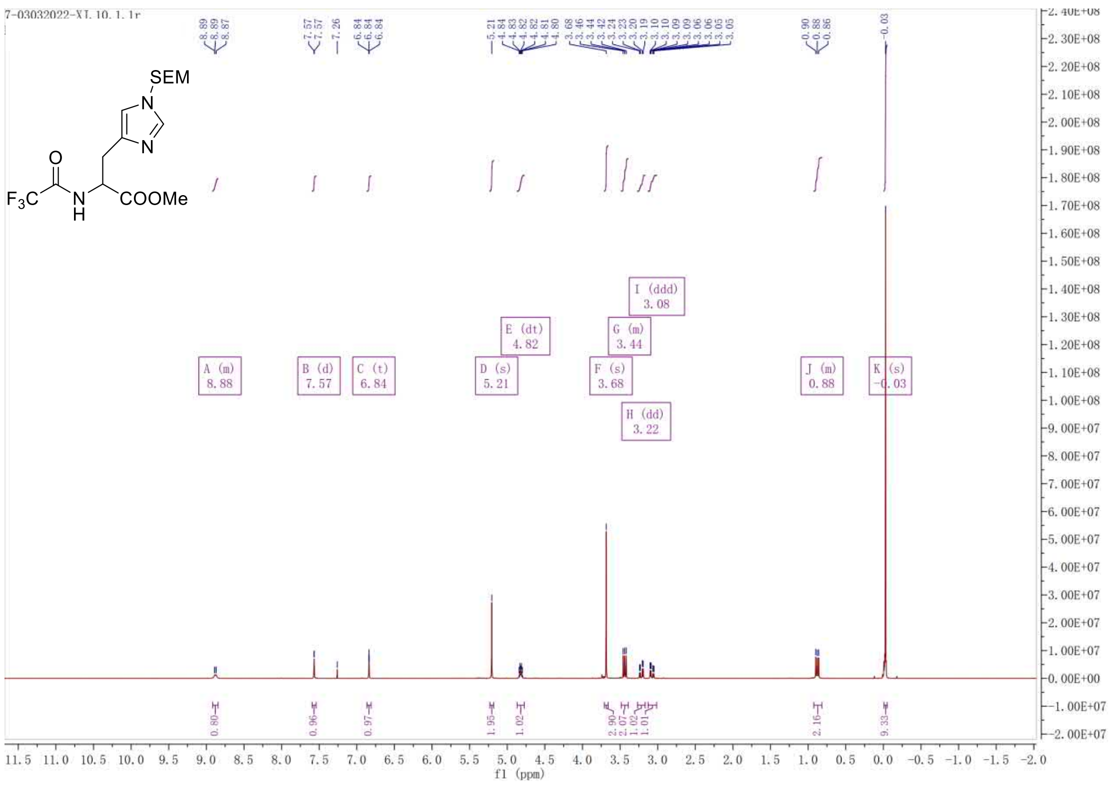

Figure S60. ^{1}H NMR spectrum of methyl N^{α}-(2,2,2-trifluoroacetyl)-N^{τ}-((2-(trimethylsilyl)ethoxy)-methyl)-L-histidinate (**8**) (400 MHz, $CDCl_3$, 25 °C).

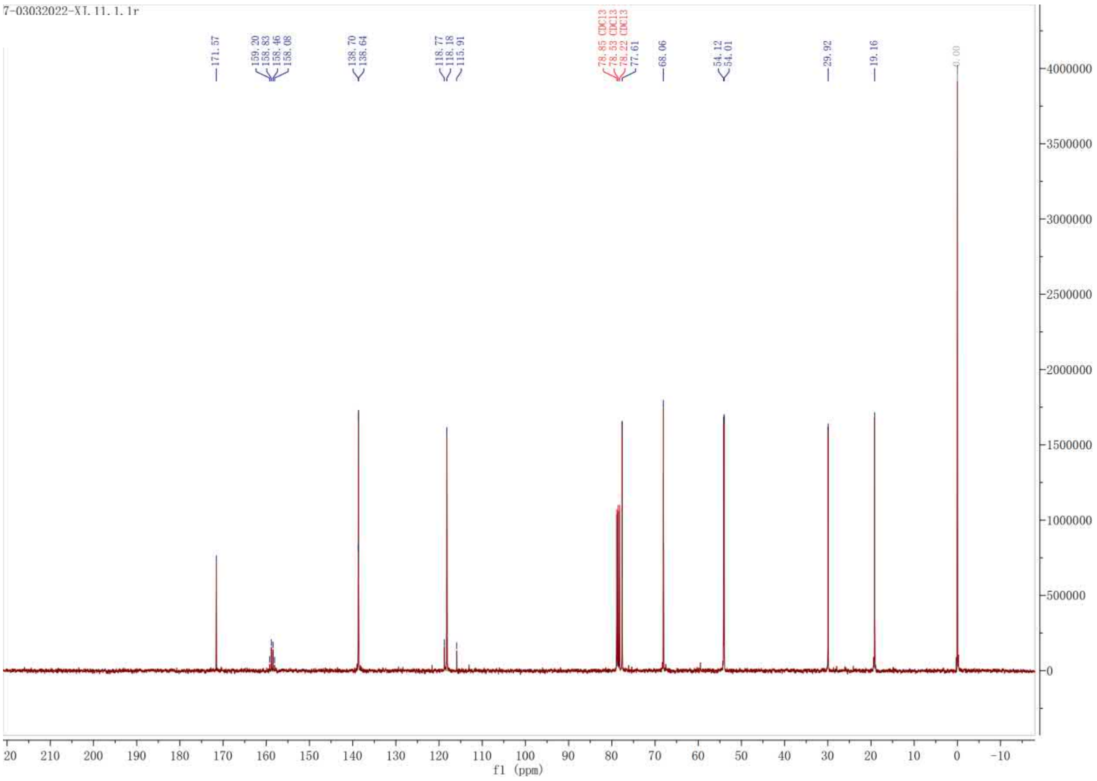

Figure S61. ^{13}C NMR spectrum of methyl N^{α}-(2,2,2-trifluoroacetyl)-N^{τ}-((2-(trimethylsilyl)ethoxy)-methyl)-L-histidinate (**8**) (100 MHz, CDCl₃, 25 °C).

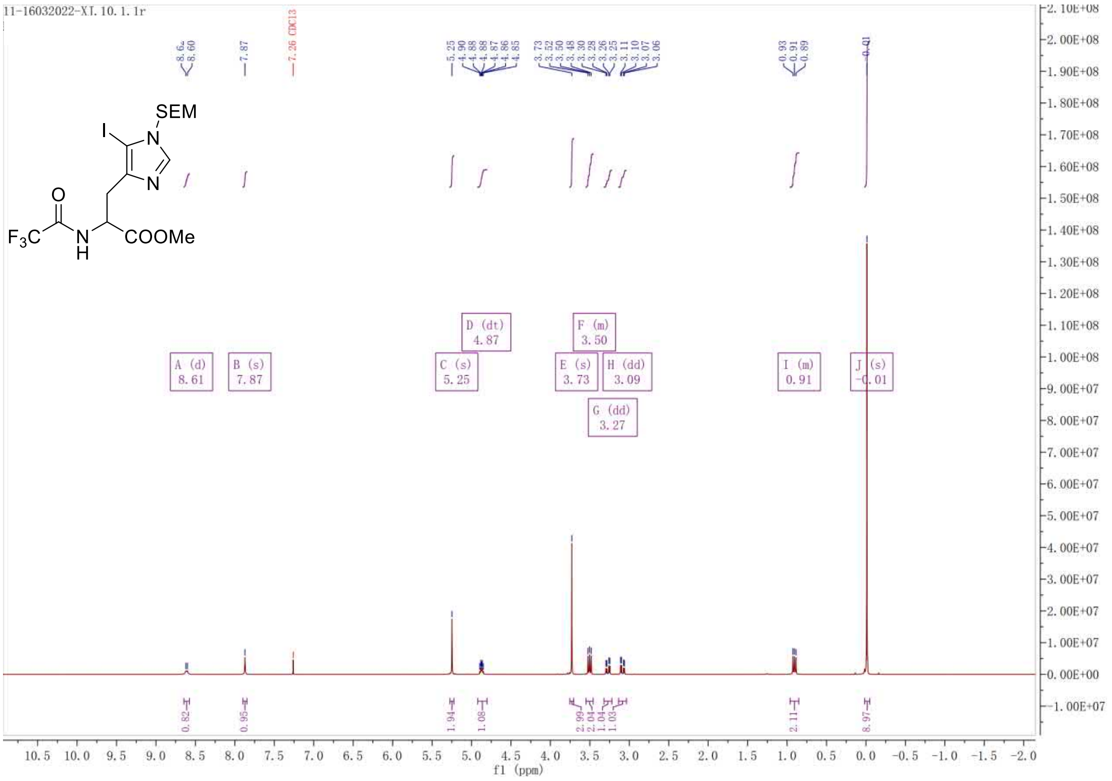

Figure S62. ^{1}H NMR spectrum of methyl (S)-3-(5-iodo-1-((2-(trimethylsilyl)ethoxy)methyl)-1H-imidazol-4-yl)-2-(2,2,2-trifluoroacetamido) propanoate (**9**) (400 MHz, CDCl₃, 25 °C).

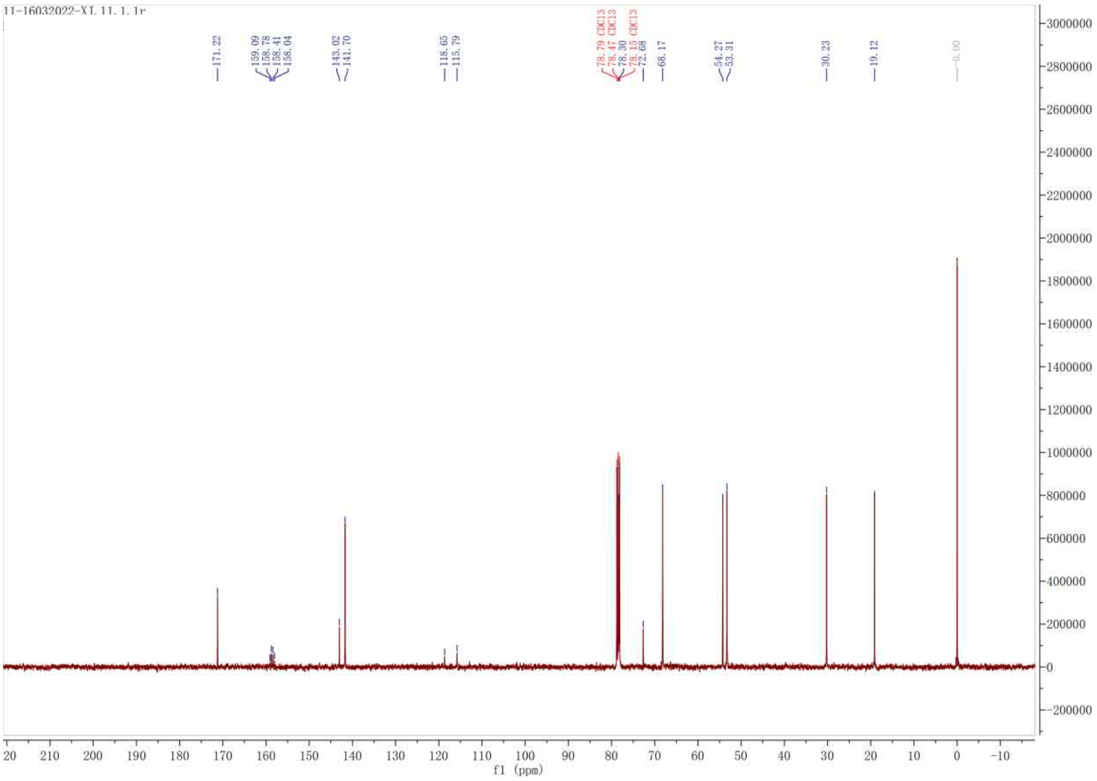

Figure S63. ^{13}C NMR spectrum of methyl (*S*)-3-(5-iodo-1-((2-(trimethylsilyl)ethoxy)methyl)-1*H*-imidazol-4-yl)-2-(2,2,2-trifluoroacetamido) propanoate (**9**) (100 MHz, $CDCl_3$, 25 °C).

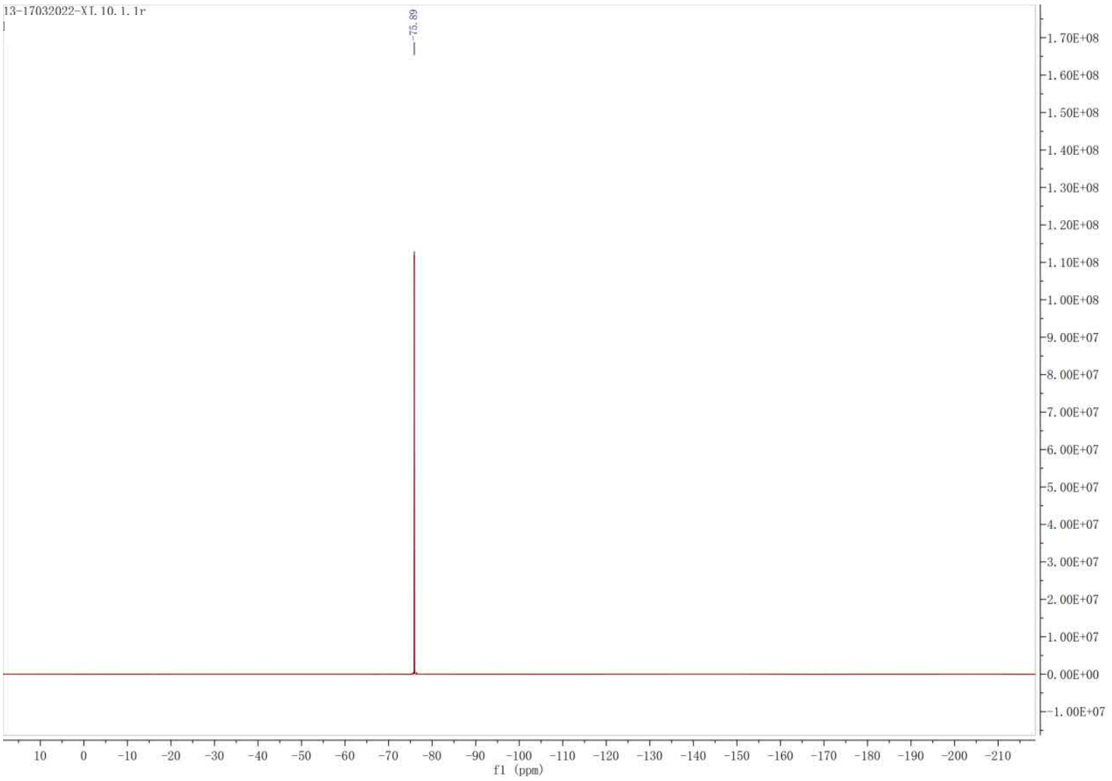

Figure S64. ^{19}F NMR spectrum of methyl (*S*)-3-(5-iodo-1-((2-(trimethylsilyl)ethoxy)methyl)-1*H*-imidazol-4-yl)-2-(2,2,2-trifluoroacetamido) propanoate (**9**) (376 MHz, $CDCl_3$, 25 °C).

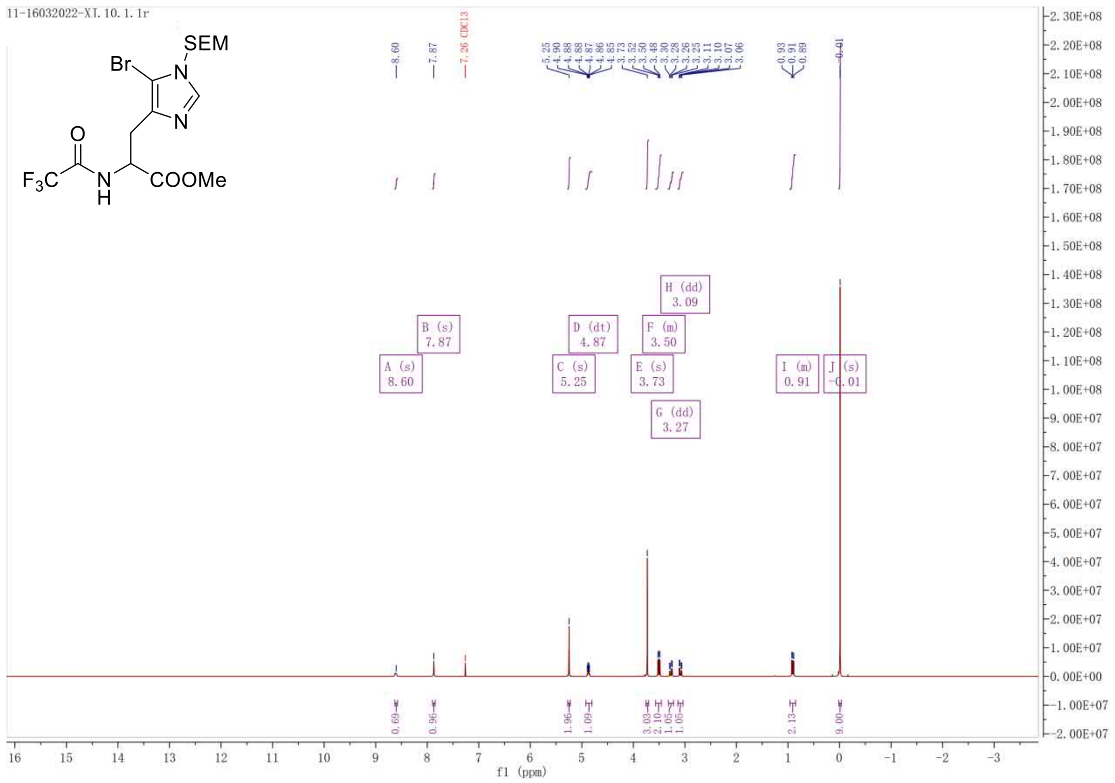

Figure S65. ^{1}H NMR spectrum of methyl (*S*)-3-(5-bromo-1-((2-(trimethylsilyl)ethoxy)methyl)-1*H*-imidazol-4-yl)-2-(2,2,2-trifluoroacetamido) propanoate (**10**) (400 MHz, $CDCl_3$, 25 °C).

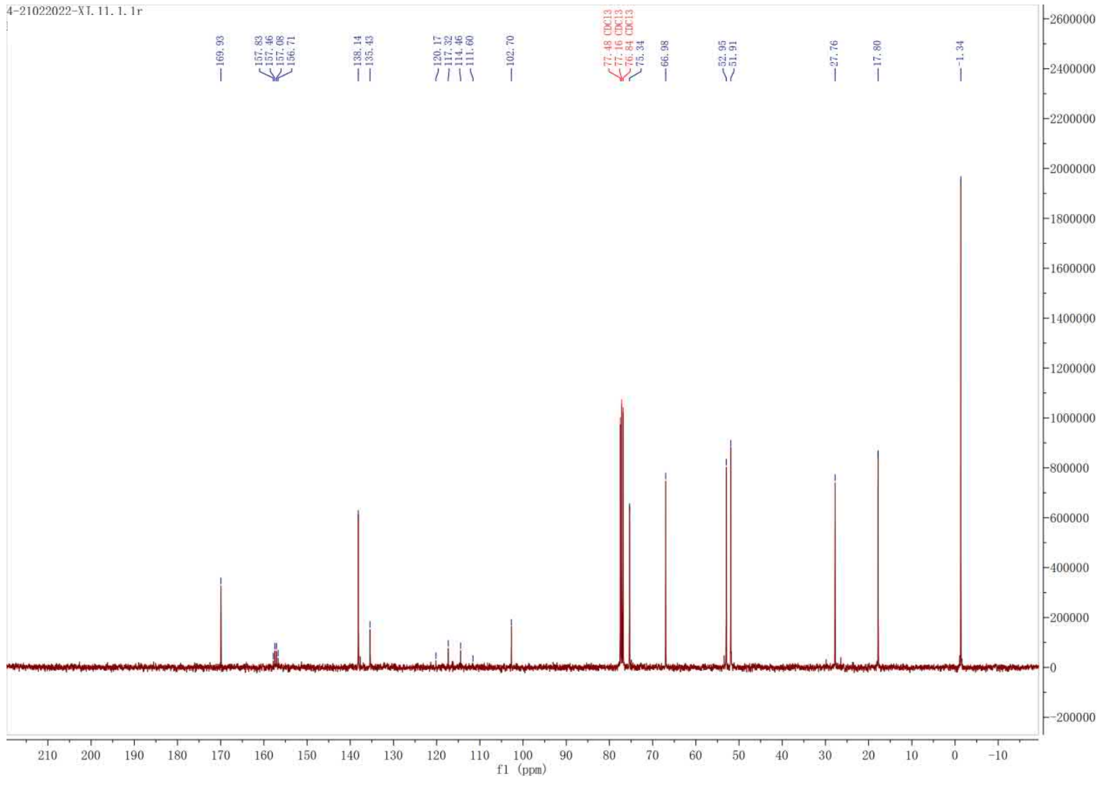

Figure S66. ^{13}C NMR spectrum of methyl (*S*)-3-(5-bromo-1-((2-(trimethylsilyl)ethoxy)methyl)-1*H*-imidazol-4-yl)-2-(2,2,2-trifluoroacetamido) propanoate (**10**) (100 MHz, $CDCl_3$, 25 °C).

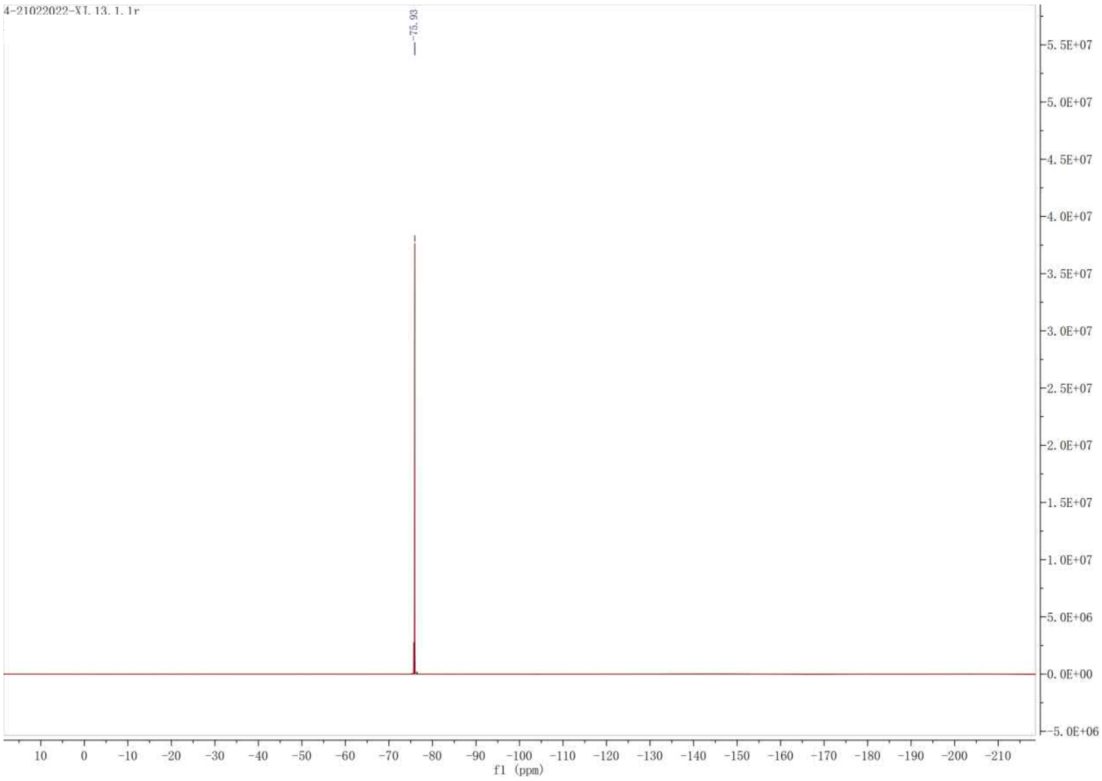

Figure S67. ^{19}F NMR spectrum of methyl (*S*)-3-(5-bromo-1-((2-(trimethylsilyl)ethoxy)methyl)-1*H*-imidazol-4-yl)-2-(2,2,2-trifluoroacetamido) propanoate (**10**) (376 MHz, $CDCl_3$, 25 °C).

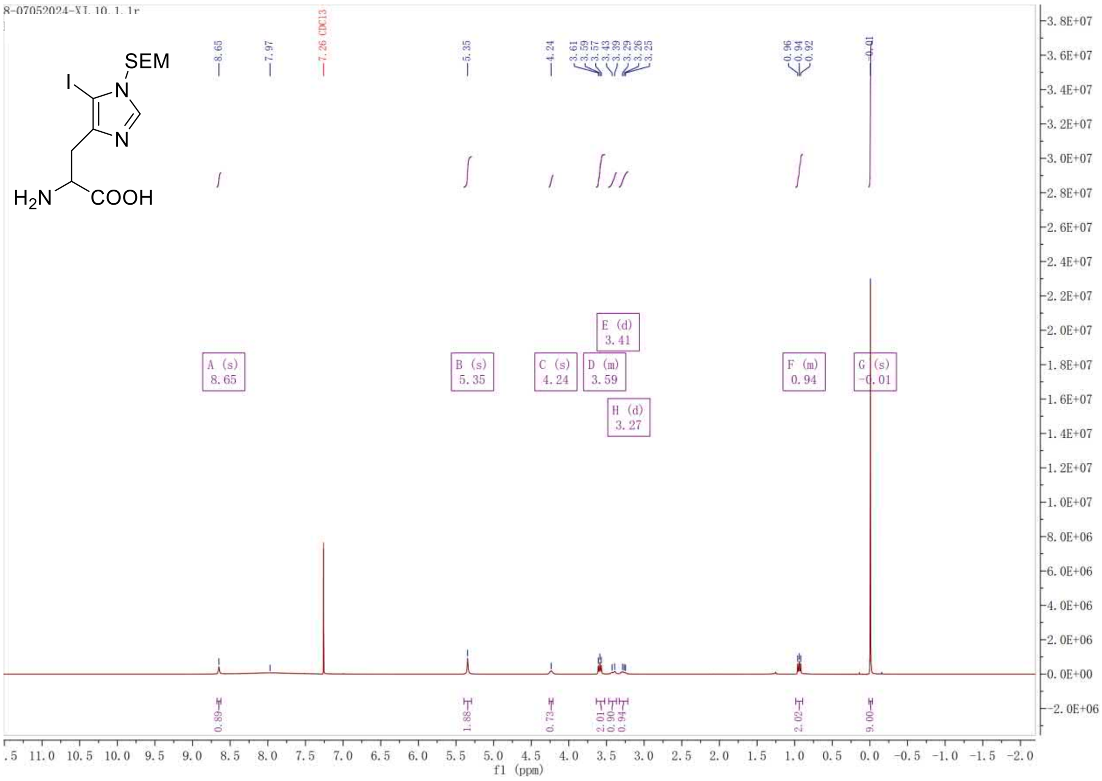

Figure S68. ^{1}H NMR spectrum of (*S*)-2-amino-3-(5-iodo-1-((2-(trimethylsilyl)ethoxy)methyl)-1*H*-imidazol-4-yl) propanoic acid (**11**) (400 MHz, $CDCl_3$, 25 °C).

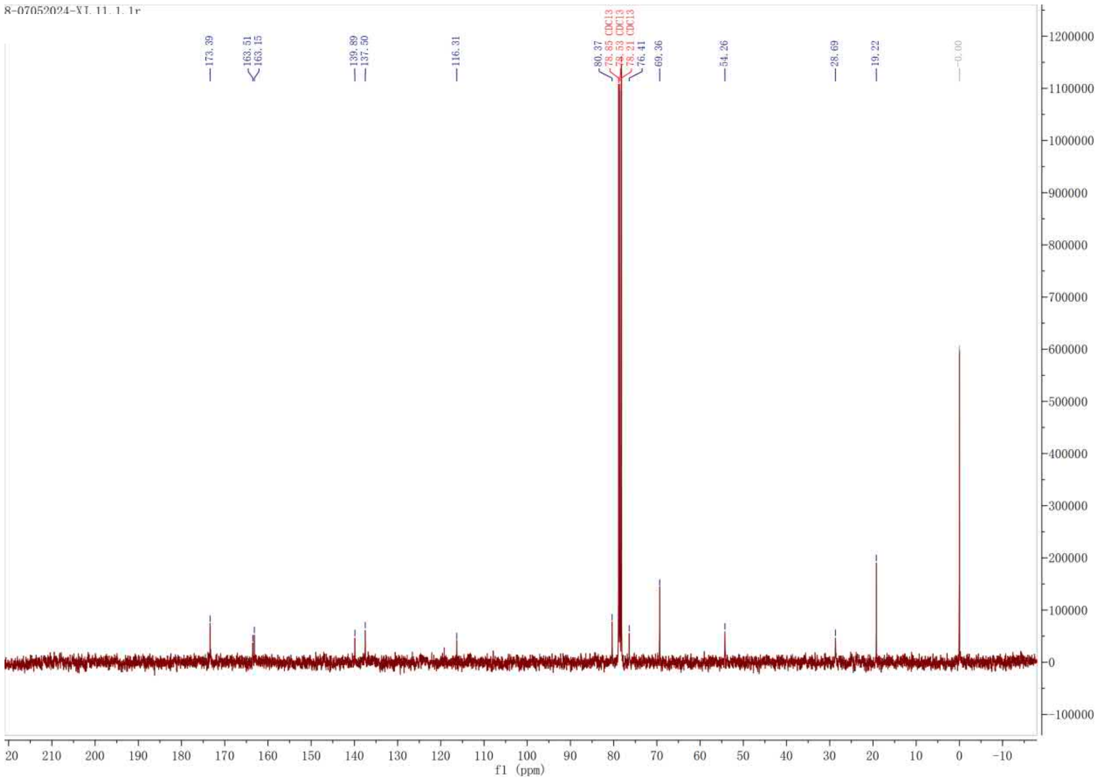

Figure S69. ^{13}C NMR spectrum of (*S*)-2-amino-3-(5-iodo-1-((2-(trimethylsilyl)ethoxy)methyl)-1*H*-imidazol-4-yl) propanoic acid (**11**) (100 MHz, $CDCl_3$, 25 °C).

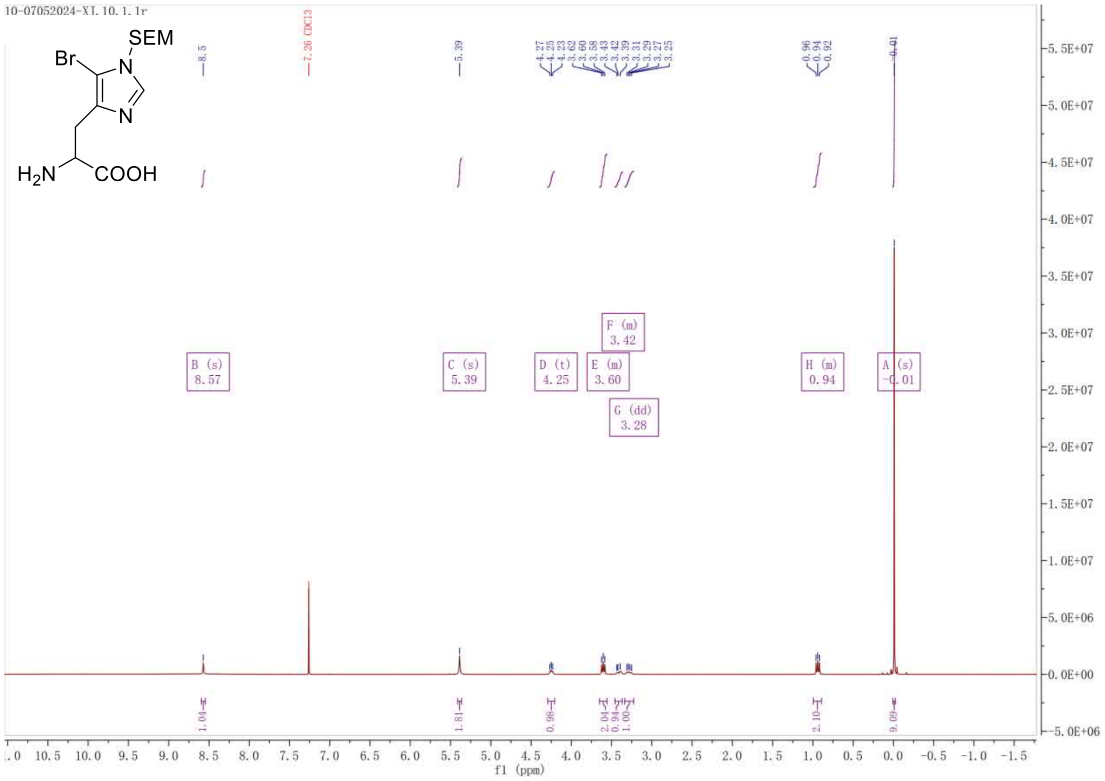

Figure S70. ^{1}H NMR spectrum of (*S*)-2-amino-3-(5-bromo-1-((2-(trimethylsilyl)ethoxy)methyl)-1*H*-imidazol-4-yl)propanoic acid (**12**) (400 MHz, $CDCl_3$, 25 °C).

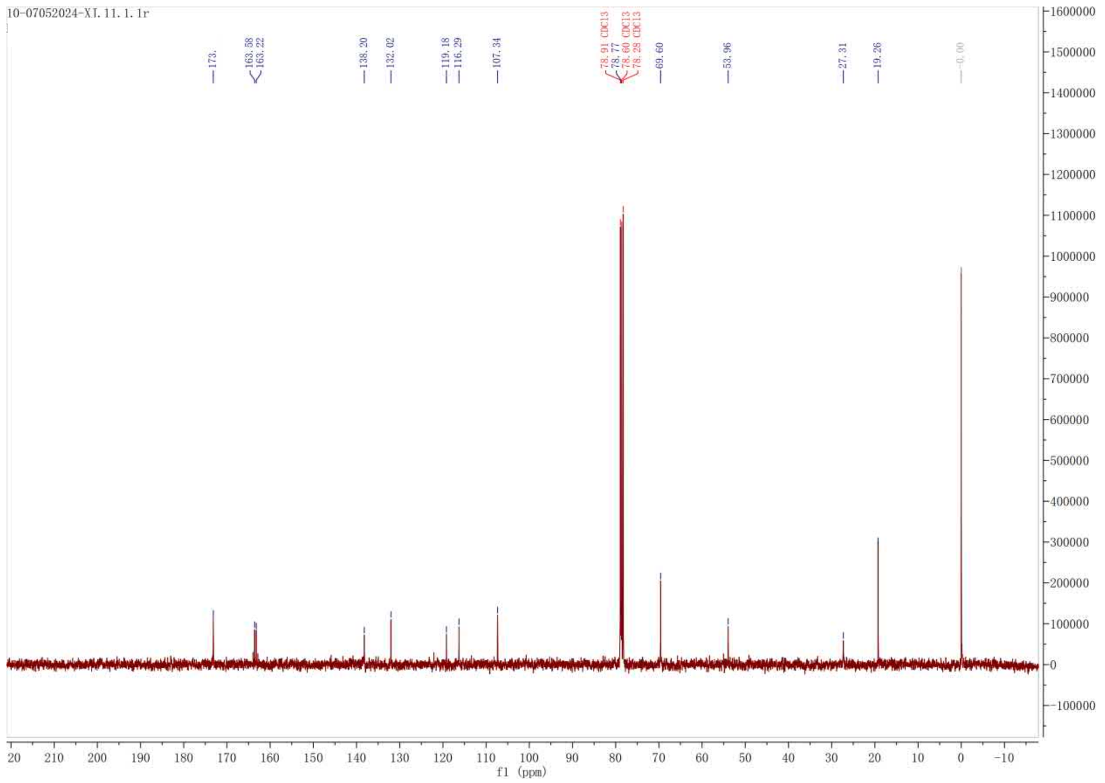

Figure S71. ^{13}C NMR spectrum of (*S*)-2-amino-3-(5-bromo-1-((2-(trimethylsilyl)ethoxy)methyl)-1*H*-imidazol-4-yl)propanoic acid (**12**) (100 MHz, $CDCl_3$, 25 °C).

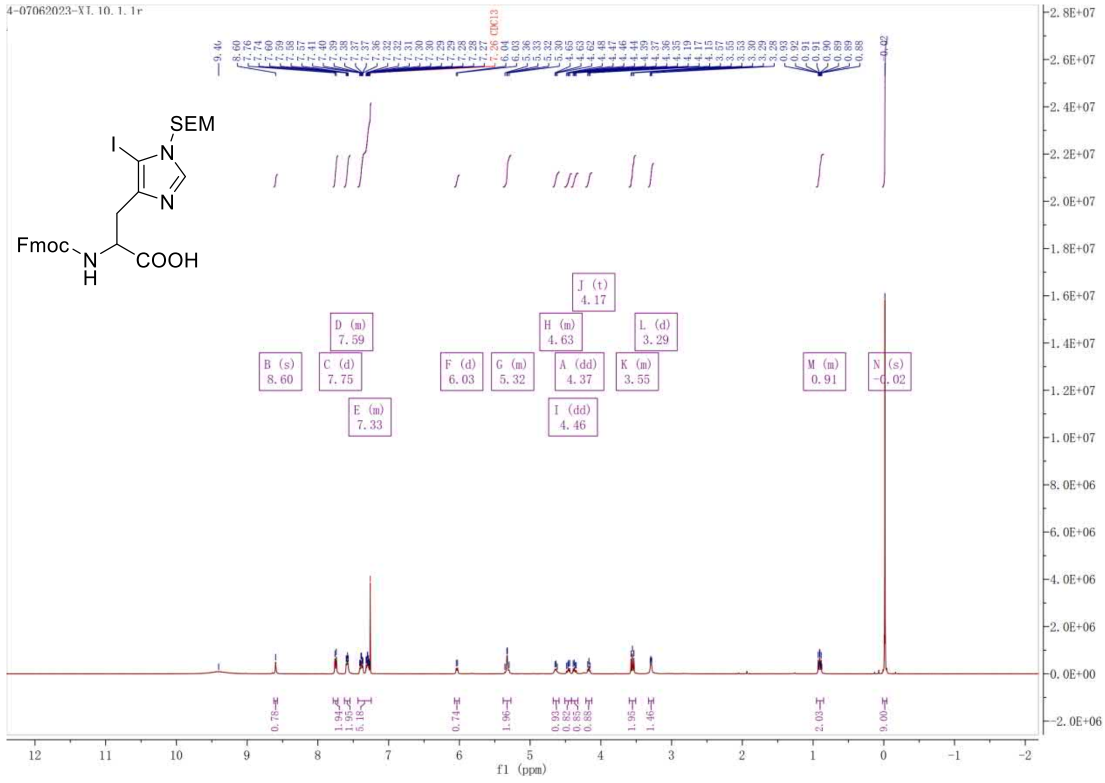

Figure S72. ^{1}H NMR spectrum of (*S*)-2-((((9*H*-fluoren-9-yl)methoxy)carbonyl)amino)-3-(5-iodo-1-((2-(trimethylsilyl)ethoxy)methyl)-1*H*-imidazol-4-yl)propanoic acid (**13**). (400 MHz, $CDCl_3$, 25 °C).

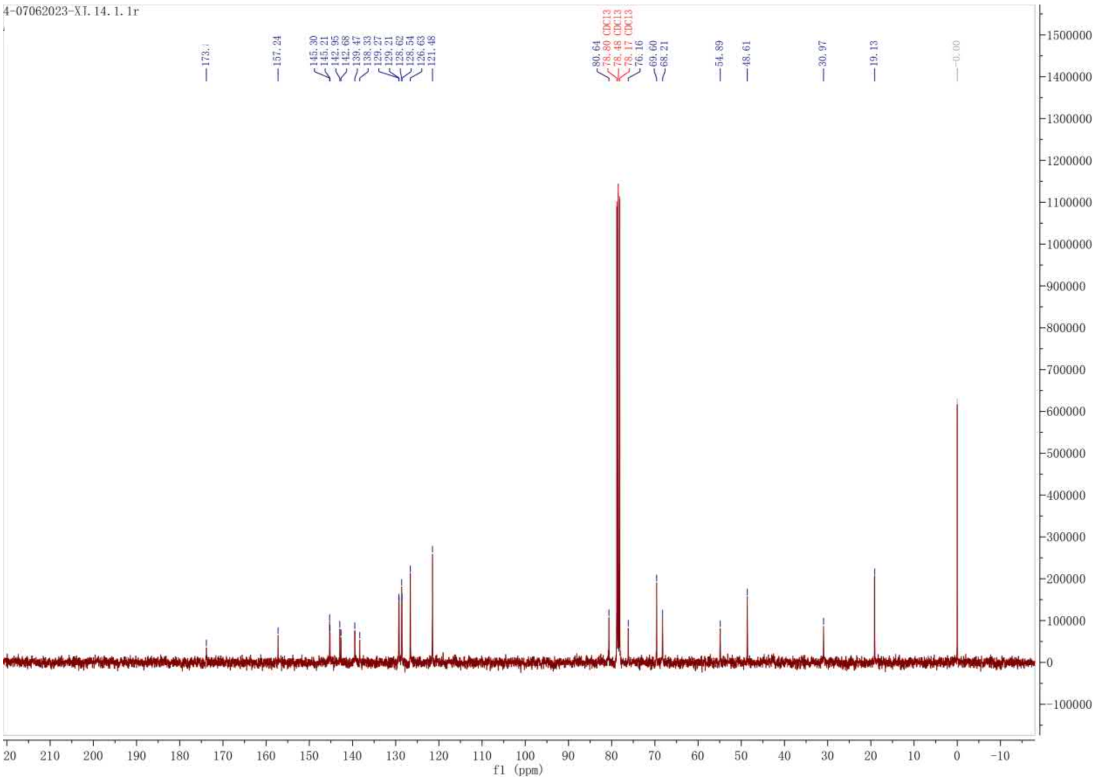

Figure S73. ^{13}C NMR spectrum of (*S*)-2-((((9*H*-fluoren-9-yl)methoxy)carbonyl)amino)-3-(5-iodo-1-((2-(trimethylsilyl)ethoxy)methyl)-1*H*-imidazol-4-yl)propanoic acid (**13**). (100 MHz, $CDCl_3$, 25 °C).

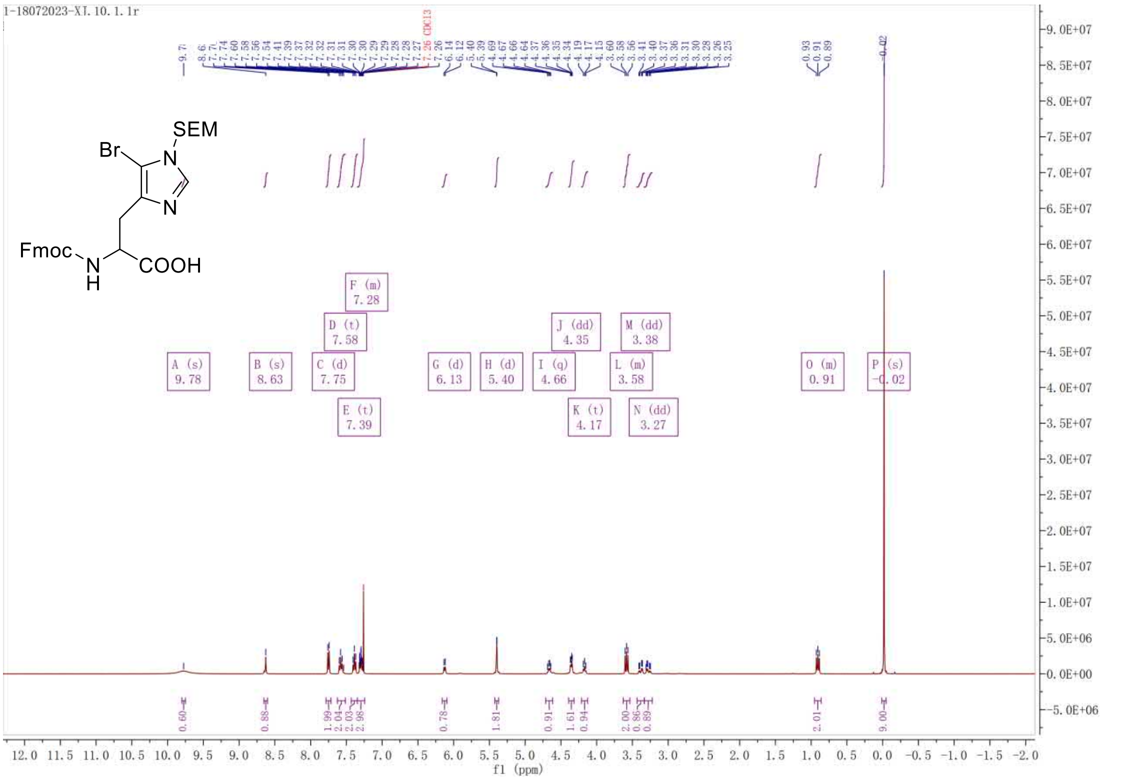

Figure S74. ^{1}H NMR spectrum of *(S)*-2-((((9*H*-fluoren-9-yl)methoxy)carbonyl)amino)-3-(5-bromo-1-((2-(trimethylsilyl)ethoxy)methyl)-1*H*-imidazol-4-yl)propanoic acid (**14**) (400 MHz, $CDCl_3$, 25 °C).

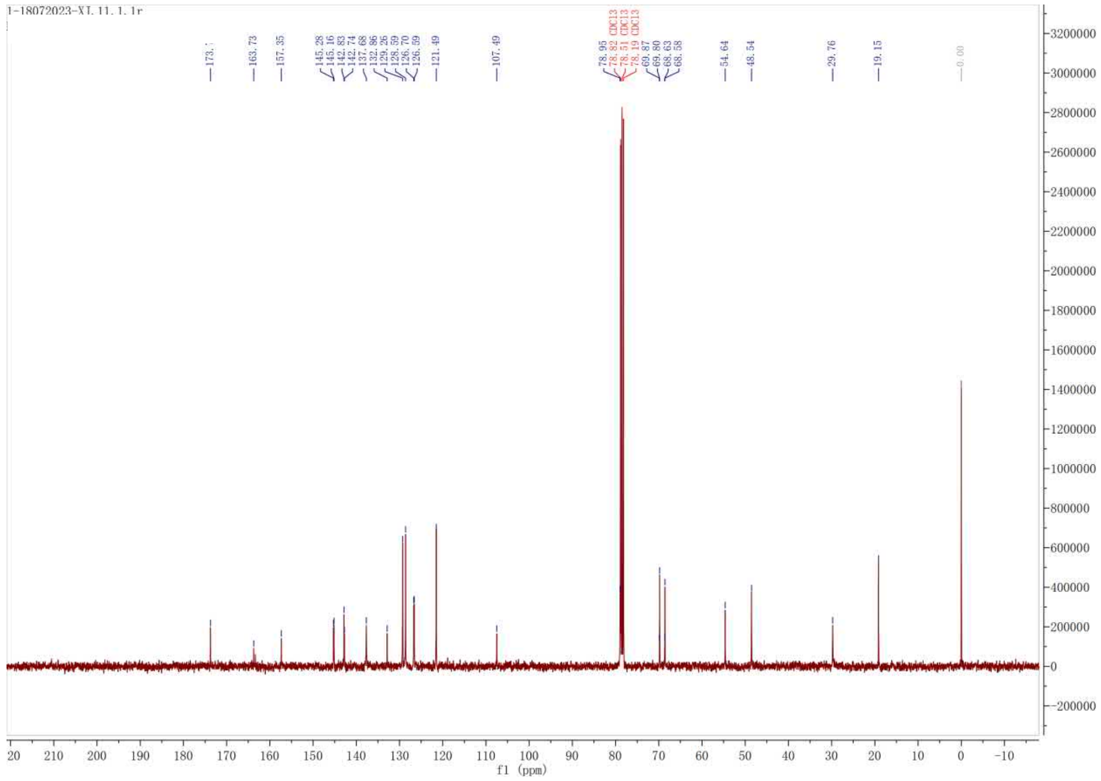

Figure S75. ^{13}C NMR spectrum of *(S)*-2-((((9*H*-fluoren-9-yl)methoxy)carbonyl)amino)-3-(5-bromo-1-((2-(trimethylsilyl)ethoxy)methyl)-1*H*-imidazol-4-yl)propanoic acid (**14**) (100 MHz, $CDCl_3$, 25 °C).

8 HRMS spectra

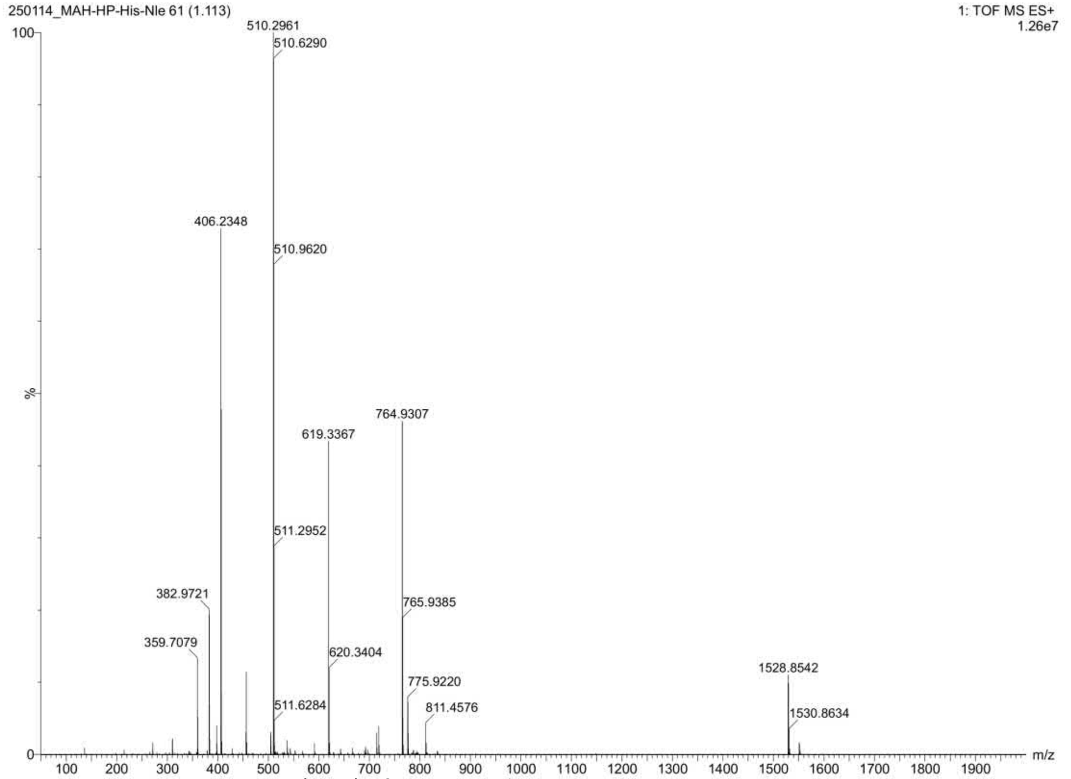

Figure S76. HRMS spectrum (ESI+) of compound **1**.

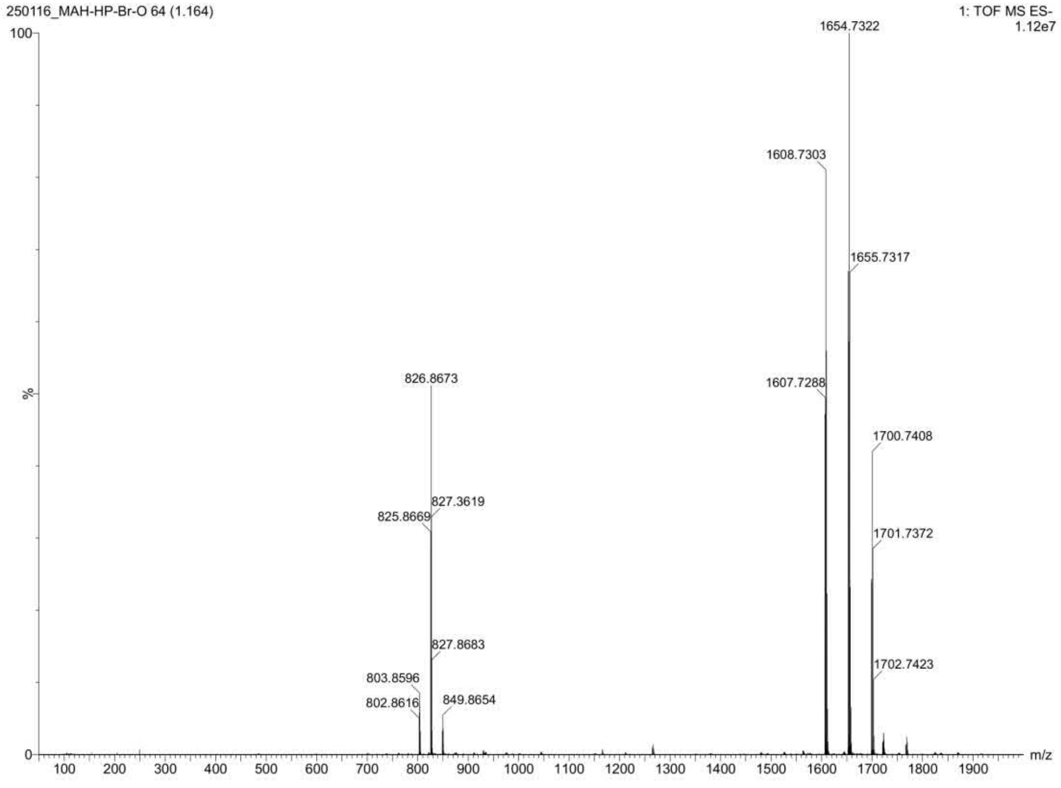

Figure S77. HRMS spectrum (ESI-) of compound **2**.

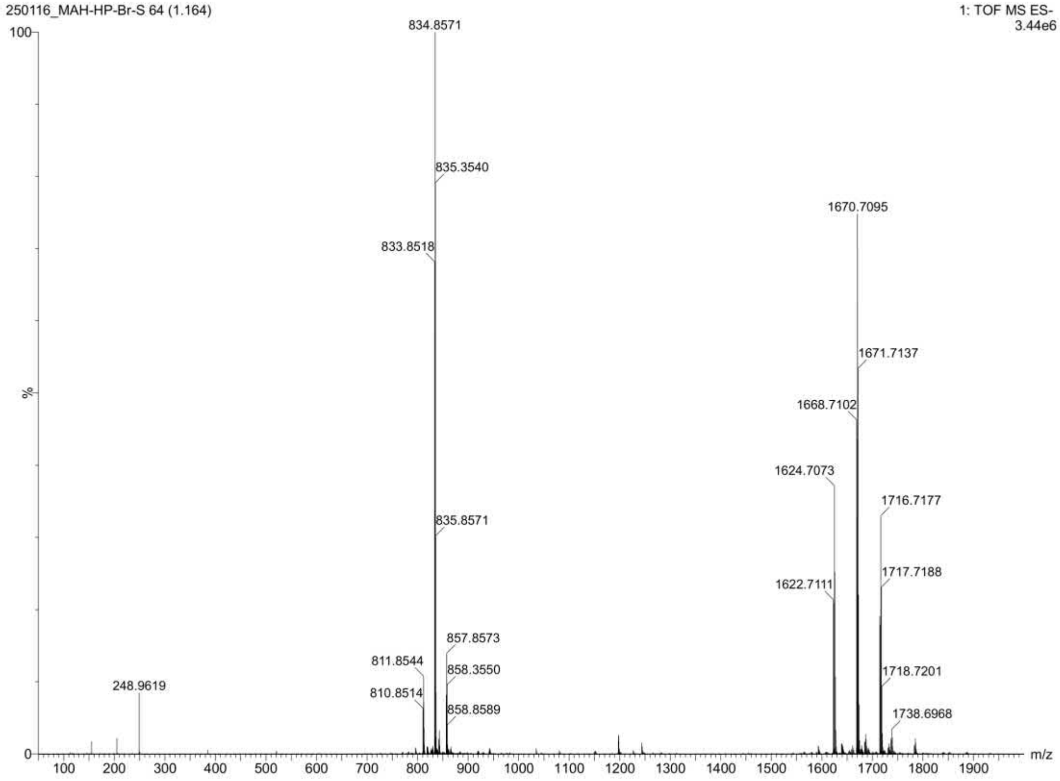

Figure S78. HRMS spectrum (ESI-) of compound **3**.

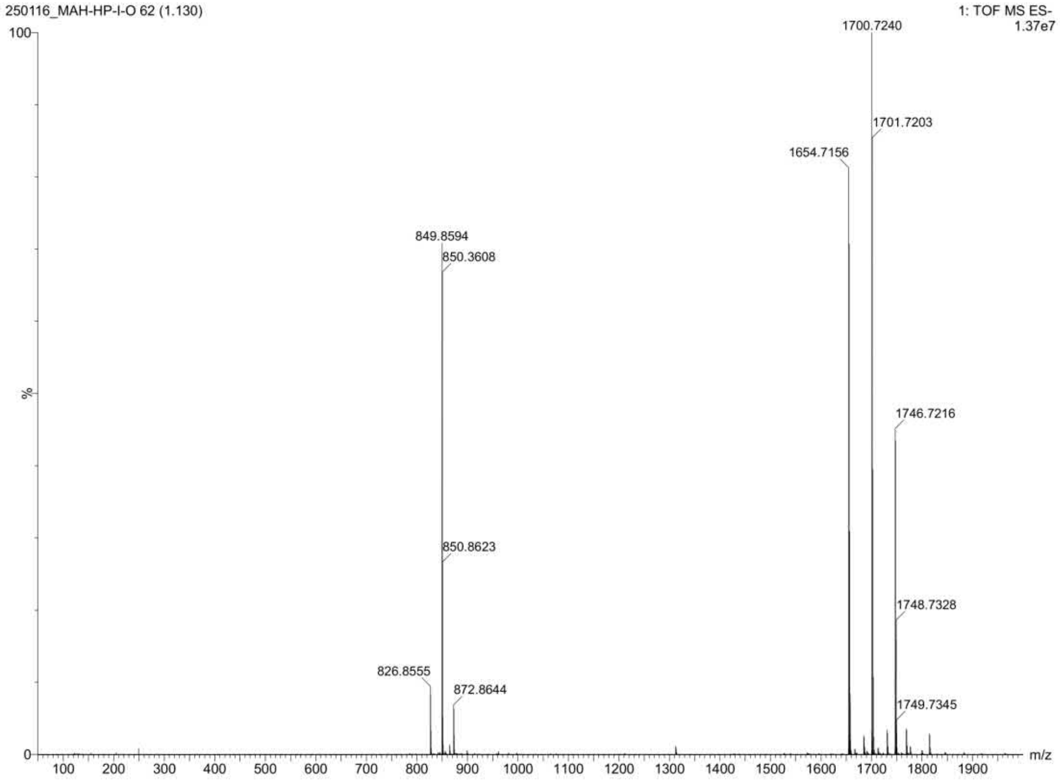

Figure S79. HRMS spectrum (ESI-) of compound **4**.

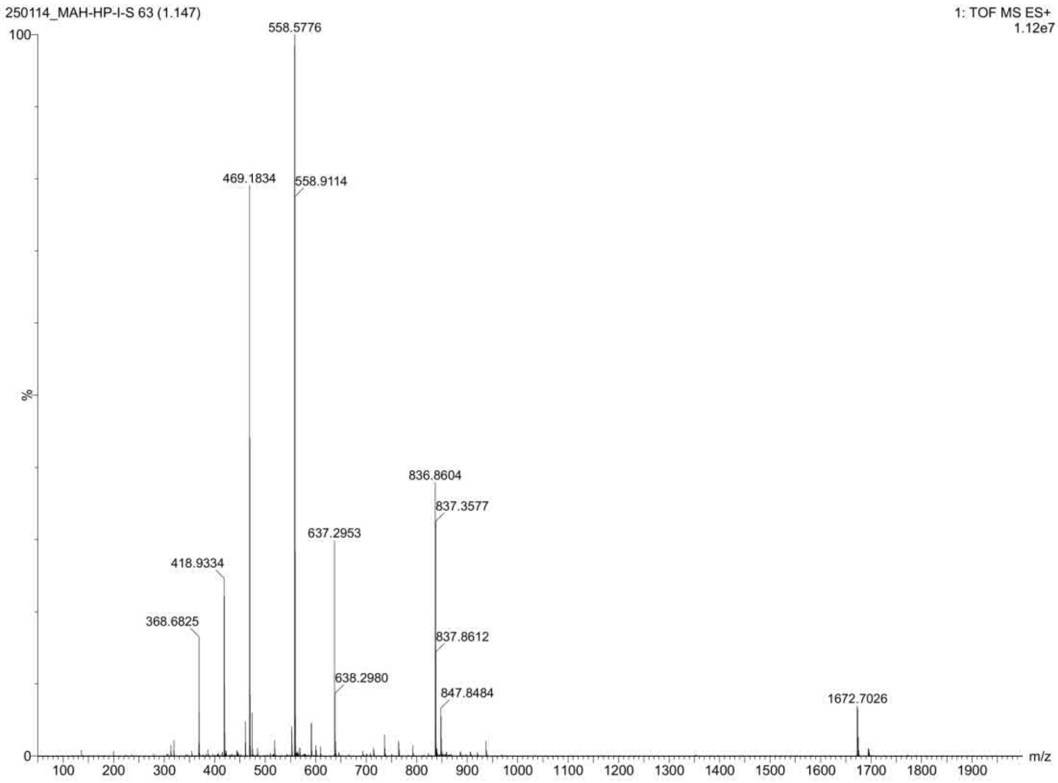

Figure S80. HRMS spectrum (ESI+) of compound **5**.

9 References

1. Mori, M.; Deodato, D.; Kasula, M.; Ferraris, D. M.; Sanna, A.; De Logu, A.; Rizzi, M.; Botta, M., Design, synthesis, SAR and biological investigation of 3-(carboxymethyl)rhodanine and aminothiazole inhibitors of Mycobacterium tuberculosis Zmp1. *Bioorg. Med. Chem. Let.* **2018,** *28* (4), 637-641.
2. Zhang, M. Preparation Method of L-Valine Methyl Ester Hydrochloride. CN101898973A.
3. Adams, H.; Bawa, R. A.; Jones, S., *N*-Alkyl oxazolidines as stereocontrol elements in asymmetric Diels–Alder cycloadditions of 9-substituted anthracene derivatives. *Org. Biomol. Chem.* **2006,** *4* (22), 4206-4213.
4. Yang, H.; Li, T.; Rong, S.; Fu, Y.; Li, S. Synthesis Method of D-threonine. CN103450040A.
5. Farley, K. A.; Koos, M. R. M.; Che, Y.; Horst, R.; Limberakis, C.; Bellenger, J.; Lira, R.; Gil-Silva, L. F.; Gil, R. R., Cross-Linked Poly-4-Acrylomorpholine: A Flexible and Reversibly Compressible Aligning Gel for Anisotropic NMR Analysis of Peptides and Small Molecules in Water. *Angew. Chem. Int. Ed.* **2021,** *60* (50), 26314-26319.
6. Castañar, L.; Sistaré, E.; Virgili, A.; Williamson, R. T.; Parella, T., Suppression of phase and amplitude J(HH) modulations in HSQC experiments. *Magn. Reson. Chem.* **2015,** *53* (2), 115-119.
7. Hu, H.; Krishnamurthy, K., Revisiting the initial rate approximation in kinetic NOE measurements. *J. Magn. Reson.* **2006,** *182* (1), 173-177.
8. Cicero, D. O.; Barbato, G.; Bazzo, R., NMR Analysis of Molecular Flexibility in Solution: A New Method for the Study of Complex Distributions of Rapidly Exchanging Conformations. Application to a 13-Residue Peptide with an 8-Residue Loop. *J. Am. Chem. Soc.* **1995,** *117* (3), 1027-1033.
9. Nevins, N.; Cicero, D.; Snyder, J. P., A Test of the Single-Conformation Hypothesis in the Analysis of NMR Data for Small Polar Molecules: A Force Field Comparison. *J. Org. Chem.* **1999,** *64* (11), 3979-3986.
10. Cross, B. P.; Schleich, T., Temperature dependence of the chemical shifts of commonly employed proton n.m.r. reference compounds. *Org. Magn. Reson.* **1977,** *10* (1), 82-85.
11. Jiménez, M. A., Protein Design. In *Protein Design: Methods and Applications*, Köhler, V., Ed. Springer NewYork: New York, 2014; pp 15-52.
12. Niebling, S.; Danelius, E.; Brath, U.; Westenhoff, S.; Erdélyi, M., The impact of interchain hydrogen bonding on β-hairpin stability is readily predicted by molecular dynamics simulation. *Pept. Sci.* **2015,** *104* (6), 703-706.
13. Pardi, A.; Billeter, M.; Wüthrich, K., Calibration of the angular dependence of the amide proton-Cα proton coupling constants, 3JHNα, in a globular protein: Use of 3JHNα for identification of helical secondary structure. *J. Mol. Biol.* **1984,** *180* (3), 741-751.
14. Losonczi, J. A.; Andrec, M.; Fischer, M. W. F.; Prestegard, J. H., Order Matrix Analysis of Residual Dipolar Couplings Using Singular Value Decomposition. *J. Magn. Reson.* **1999,** *138* (2), 334-342.
15. Navarro-Vázquez, A., MSpin-RDC. A program for the use of residual dipolar couplings for structure elucidation of small molecules. *Magn. Reson. Chem.* **2012,** *50* (S1), S73-S79.
16. Cornilescu, G.; Marquardt, J. L.; Ottiger, M.; Bax, A., Validation of Protein Structure from Anisotropic Carbonyl Chemical Shifts in a Dilute Liquid Crystalline Phase. *J. Am. Chem. Soc.* **1998,** *120*, 6836-6837.
17. Krishnan, R.; Binkley, J. S.; Seeger, R.; Pople, J. A., Self-consistent molecular orbital methods. XX. A basis set for correlated wave functions. *J. Chem. Phys.* **1980,** *72* (1), 650-654.
18. Igel-Mann, G.; Stoll, H.; Preuss, H., Pseudopotentials for main group elements (IIIa through VIIa). *Mol. Phys.* **1988,** *65* (6), 1321-1328.

19. Scalmani, G.; Frisch, M. J., Continuous surface charge polarizable continuum models of solvation. I. General formalism. *J. Chem. Phys.* **2010,** *132* (11), 114110.
20. Boys, S. F.; Bernardi, F., The calculation of small molecular interactions by the differences of separate total energies. Some procedures with reduced errors. *Mol. Phys.* **1970,** *19* (4), 553-566.
21. Frisch, M. J.; Trucks, G. W.; Schlegel, H. B.; Scuseria, G. E.; Robb, M. A.; Cheeseman, J. R.; Scalmani, G.; Barone, V.; Petersson, G. A.; Nakatsuji . , H.; et al. *Gaussian 16 Rev. C.01*, Gaussian, Inc.: Wallingford, CT, 2016.
